# Selective and stable base pairing by alkynylated nucleosides featuring a spatially-separated recognition interface

**DOI:** 10.1093/nar/gkac140

**Published:** 2022-03-02

**Authors:** Hidenori Okamura, Giang Hoang Trinh, Zhuoxin Dong, Yoshiaki Masaki, Kohji Seio, Fumi Nagatsugi

**Affiliations:** Institute of Multidisciplinary Research for Advanced Materials, Tohoku University, 2-1-1 Katahira, Aoba-ku, Sendai, Miyagi 980-8577, Japan; Department of Chemistry, Graduate School of Science, Tohoku University, 6-3 Aramaki Aza-Aoba, Aoba-ku, Sendai, Miyagi 980-8578, Japan; Institute of Multidisciplinary Research for Advanced Materials, Tohoku University, 2-1-1 Katahira, Aoba-ku, Sendai, Miyagi 980-8577, Japan; Department of Chemistry, Graduate School of Science, Tohoku University, 6-3 Aramaki Aza-Aoba, Aoba-ku, Sendai, Miyagi 980-8578, Japan; Institute of Multidisciplinary Research for Advanced Materials, Tohoku University, 2-1-1 Katahira, Aoba-ku, Sendai, Miyagi 980-8577, Japan; Department of Chemistry, Graduate School of Science, Tohoku University, 6-3 Aramaki Aza-Aoba, Aoba-ku, Sendai, Miyagi 980-8578, Japan; Department of Life Science and Technology, Tokyo Institute of Technology, 4259 Nagatsuta-cho, Midori-ku, Yokohama, Kanagawa 226-8501, Japan; JST, PRESTO, 4-1-8 Honcho, Kawaguchi, Saitama 332-0012, Japan; Department of Life Science and Technology, Tokyo Institute of Technology, 4259 Nagatsuta-cho, Midori-ku, Yokohama, Kanagawa 226-8501, Japan; Institute of Multidisciplinary Research for Advanced Materials, Tohoku University, 2-1-1 Katahira, Aoba-ku, Sendai, Miyagi 980-8577, Japan; Department of Chemistry, Graduate School of Science, Tohoku University, 6-3 Aramaki Aza-Aoba, Aoba-ku, Sendai, Miyagi 980-8578, Japan

## Abstract

Unnatural base pairs (UBPs) which exhibit a selectivity against pairing with canonical nucleobases provide a powerful tool for the development of nucleic acid-based technologies. As an alternative strategy to the conventional UBP designs, which involve utility of different recognition modes at the Watson–Crick interface, we now report that the exclusive base pairing can be achieved through the spatial separation of recognition units. The design concept was demonstrated with the alkynylated purine (^N^Pu, ^O^Pu) and pyridazine (^N^Pz, ^O^Pz) nucleosides endowed with nucleobase-like 2-aminopyrimidine or 2-pyridone (‘pseudo-nucleobases’) on their major groove side. These alkynylated purines and pyridazines exhibited exclusive and stable pairing properties by the formation of complementary hydrogen bonds between the pseudo-nucleobases in the DNA major groove as revealed by comprehensive *T*_m_ measurements, 2D-NMR analyses, and MD simulations. Moreover, the alkynylated purine-pyridazine pairs enabled dramatic stabilization of the DNA duplex upon consecutive incorporation while maintaining a high sequence-specificity. The present study showcases the separation of the recognition interface as a promising strategy for developing new types of UBPs.

## Graphical Abstract

**Graphical Abstract:**
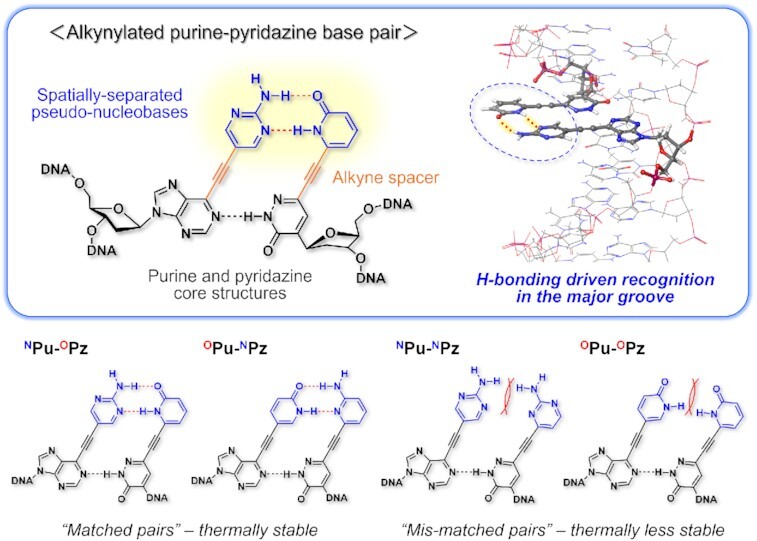
A new type of unnatural base pairing was demonstrated by the alkynylated purine (^N^Pu, ^O^Pu) and pyridazine (^N^Pz, ^O^Pz) nucleosides. These nucleosides exhibited selective and thermally stable base pairing via hydrogen-bonding in the DNA major groove, providing an alternative design basis for UBPs toward DNA-based biotechnologies.

## INTRODUCTION

Watson–Crick base pairing is a molecular interaction which underlies the unique properties of DNA including sequence-specific hybridization as well as the coding and replication of genetic information. The exclusive pairing ability of the A–T and G–C pairs has provided a functional basis for numerous biotechnologies, as represented by the construction of DNA nanostructures for applications in molecular assembly, diagnostics and drug delivery (1–3) along with the recently emerging DNA computing system (4,5) and DNA-encoded drug discovery (6,7). Furthermore, the encodable nature of DNA with the recent mechanistic unravelling of the genetic system enabled the reprogramming of the gene in living organisms, allowing the engineering of artificial proteins and manipulation of the cellular functions (8,9). The dimensions of such nucleic acid-based technologies can be dramatically augmented if the combination of encodable components in the DNA is expanded beyond the canonical Watson–Crick pairs, thus there is a growing attention toward the creation of unnatural base pairs (UBPs) (10–12).

Over the past decades, considerable efforts have been made by researchers to invent UBPs which exhibit a selectivity against natural nucleobases in terms of base pairing and enzymatic reactions. For example, Benner created a genetic system consisting of up to four sets of base pairs by adopting mutually exclusive hydrogen bond donor-acceptor patterns (13,14). Alternatively, Matsuda and Minakawa developed a series of four-hydrogen bonded base pairs with an expanded ring system and demonstrated stable pairing (15,16). Very recently, Okamoto reported the hydrogen-bonding type UBPs adopting an *anti*-*syn* orientation (17). In another approach, Kool, Hirao and Romesberg developed hydrophobic base pairs which exhibit highly orthogonal pairing during enzymatic DNA synthesis by shape-complementarity and packing forces (18–20). Furthermore, Shionoya and Carell reported the selective and stable base pairing by utilizing metal ion coordination in the molecular design (21–23). These UBPs have been shown to augment the functionalities of nucleic acids as exemplified by the generation of high-affinity DNA aptamers, application in DNA nanotechnologies, synthesis of unnatural proteins by genetic code expansion and creation of a semi-synthetic organism (24).

In the light of the promising utility of the UBPs in biotechnology and synthetic biology studies, expansion of the molecular repertoires of UBPs would be of great significance. In particular, creation of UBPs that exhibit both a high selectivity and thermal stability in the DNA duplex remains a challenging goal. Most of the conventional hydrogen-bond type UBPs show a high thermal stability in the DNA duplex, however, the chemical instability and tautomerization often hamper the selectivity against pairing with canonical nucleobases (25,26). The hydrophobic base pairs, on the other hand, exhibit a high fidelity in polymerase mediated amplification; however, their thermal stability in the DNA duplexes tends to be rather law except for the self-pairs (27,28).

**Figure 1.**
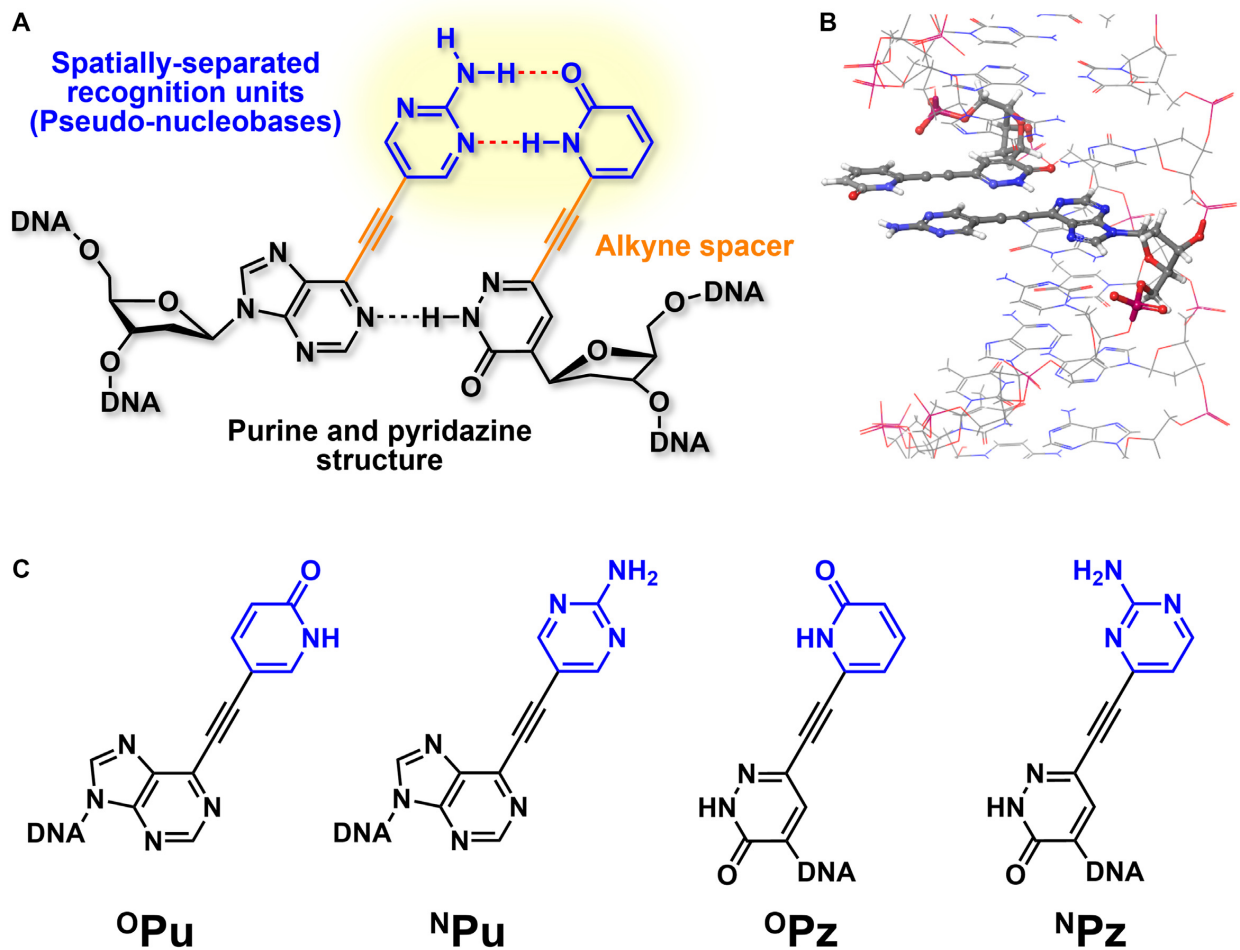
(**A**) Design concept of the alkynylated purine and pyridazine base pair featuring the spatially-separated pseudo-nucleobases. (**B**) The model structure depicting the formation of hydrogen bonds between the pseudo-nucleobases in the major groove of the DNA duplex. The model was built with MacroModel. (**C**) The structures of the alkynylated purine (^O^Pu, ^N^Pu) and pyridazine (^O^Pz, ^N^Pz) nucleosides synthesized in this study.

While the previously reported UBPs exhibit exclusive pairing properties by adopting different types of intermolecular interactions at the Watson–Crick position, spatial separation of the recognition modules is a potential design strategy which has not been reported to date. Introduction of the mutually interactive moieties at a distant position from the canonical base pairs would provide a high selectivity against undesired pairing with the canonical nucleobases. To explore the validity of such design concept toward development of highly selective and thermally stable UBP, in this study, we designed alkynylated purine and pyridazine nucleosides (Figure 1A). Both the purine and pyridazine derivatives consist of the three following functional moieties: (i) the purine and pyridazine core structures for maintaining the stacking continuity within the double helix, (ii) nucleobase-like units termed ‘pseudo-nucleobases’ as the recognition modules and (iii) the alkyne spacer to dislocate and align the respective pseudo-nucleobases for hydrogen bond-based recognition in the major groove. We envisioned that such molecular design would enable high thermal stability while assuring the selectivity against pairing with the canonical nucleobases (Figure 1B).

The critical chemical question associated with the present molecular design is whether such hydrogen bond-driven recognition can be established in the hydrophilic environment of the DNA major groove. To address this point, 2-pyridone and 2-aminopyrimidine, which are known to function as nucleobase surrogates (29–32) and are less susceptible to protonation/deprotonation under neutral pH (33,34), were introduced as the pseudo-nucleobases of alkynylated purines (^O^Pu, ^N^Pu) and pyridazines (^O^Pz, ^N^Pz), respectively (Figure 1C). The hypothesis was that these alkynylated purine and pyridazine nucleosides would exhibit exclusive base-pairing according to the hydrogen bond complementarity of the pseudo-nucleobases (e.g. ^N^Pu–^O^Pz and ^O^Pu–^N^Pz) if the formation of the hydrogen-bond based recognition takes place in the major groove.

## MATERIALS AND METHODS

### General

All the chemicals and solvents were purchased from Sigma-Aldrich, the Tokyo Chemical Industry, FUJIFILM Wako Pure Chemical, Kanto Chemical, and used without further purification. The reactions were conducted under an argon atmosphere in oven-dried glassware unless otherwise specified. The NMR spectra were recorded with a Bruker AVANCE III 400 spectrometer and a Bruker AVANCE III 600 spectrometer. The chemical shifts were calibrated to the residual solvents as follows: ^1^H NMR (400 MHz)—CDCl_3_ (7.26 ppm), CD_3_OD (4.87 ppm); ^13^C NMR (151 MHz)—CDCl_3_ (77.16 ppm), CD_3_OD (49.00 ppm). All the NMR spectra were analyzed using Bruker TopSpin 3.6.2. The high-resolution electrospray ionization mass spectrometry measurement was performed using a Bruker MicrOTOF-QII. The oligodeoxynucleotides (ODNs) used in this study were synthesized on a 1 μmol scale using a DNA automated synthesizer (Applied Biosystems 392 DNA/RNA Synthesizer). The phosphoramidites of the canonical deoxyribonucleosides were purchased from Glen Research and Sigma-Aldrich. The ODNs composed of all-canonical nucleosides were purchased from Japan Bio Services Co., Ltd. (Japan). The HPLC purification and analysis were performed using a JASCO HPLC system (PU-2089 plus, UV-2075 plus, CO-2067 plus). The structural integrity of the synthesized ODNs was analyzed by a MALDI-TOF mass measurement using a Bruker Daltonics Autoflex Speed instrument with a mixture of 3-hydroxypicolinic acid and diammonium hydrogen citrate as a matrix. The UV absorbance and melting curves were measured using a Beckman Coulter DU800 spectrophotometer equipped with a High Performance Temperature Controller and 10 mm quartz cells and a JASCO V-730 UV–visible Spectrophotometer equipped with a PAC-743R Automatic 6/8-Position Peltier Cell Changer. The CD spectra were measured by a JASCO J-720WI spectropolarimeter equipped with 10 mm cylindrical micro cells.

### Synthesis and characterization of the alkynylated purine and pyridazine derivatives

The syntheses and spectroscopic characterization of the phosphoramidite building blocks and fully-deprotected nucleosides of ^N^Pu, ^O^Pu, ^N^Pz and ^O^Pz are detailed in the Supporting Information. The molar extinction coefficients of ^N^Pu, ^O^Pu, ^N^Pz, ^O^Pz at 260 nm (E260; M^–1^ cm^–1^) were determined by measuring the UV absorption of an aqueous solution of each nucleoside; a solution of the nucleoside in DMSO (250 μM; concentration determined by NMR quantification) was titrated into 250 μl of ddH_2_O or 10 mM sodium phosphate buffer at pH 7.0, and the UV absorption was measured at 240–400 nm. The E260 values were calculated from the average of three individual titrations as follows: E260 in ddH_2_O—^N^Pu: 13730, ^O^Pu: 10020, ^N^Pz: 24970, ^O^Pz: 14940; E260 in 10 mM sodium phosphate buffer (pH 7.0)—^N^Pu: 11440, ^O^Pu: 8520, ^N^Pz: 23590, ^O^Pz: 14030.

### Preparation of the oligodeoxynucleotides

The ODNs containing ^N^Pu, ^O^Pu, ^N^Pz and ^O^Pz were synthesized in the DMT-OFF mode using ultra-mild phosphoramidites (Pac-dA, Tac-dG, Ac-dC, T) with BTT in CH_3_CN as an activator, 3% DCA in CH_2_Cl_2_ as a deblocking solution, phenoxyacetic anhydride in THF-pyridine as a capping reagent and 0.02 M iodine in THF-pyridine-H_2_O as an oxidizer. After the solid-phase DNA syntheses, removal of the 2-trimethylsilylethyl groups on ^O^Pu, ^O^Pz and ^N^Pz were performed by treating the CPG with a solution of zinc bromide in *i*PrOH-CH_3_NO_2_ (500 μl; prepared by dissolving 2.5 g of ZnBr_2_ in 3 ml of *i*PrOH-CH_3_NO_2_ (1:1) in a shaker for 6 h at room temperature. The supernatant was discarded, and the CPG was washed with EtOH followed by distilled water. The deprotection of the ODNs was achieved by treating the CPG with NH_4_OH (28%, 1 ml) overnight at room temperature. The CPG was filtered off using a membrane filter, and the filtrate was evaporated to dryness using a centrifugal evaporator. The crude ODNs were purified by preparative reverse-phase HPLC using a Nacalai Tesque COSMOSIL 5C_18_-MS-II column (10 × 250 mm) with 0.1 M triethylammonium acetate buffer at pH 7.0 (buffer A) and acetonitrile (buffer B). A gradient of 5 to 20% of buffer B in 20 min was applied at the flow rate of 3 ml/min at 35°C, and the peaks were detected at 254 nm. Analytical reverse-phase HPLC was performed using a Nacalai Tesque COSMOSIL 5C_18_-MS-II column (4.6 × 250 mm) with the same conditions except for the flow rate (1 ml/min).

### UV melting temperature measurement

A solution (300 μl) containing equimolar amounts of the ODNs (2 μM each as final concentration), 10 mM sodium phosphate buffer and 150 mM NaCl at pH 7.0 was heated at 80°C (single incorporation) or 90°C (multiple incorporation) for 5 min, then gradually cooled to room temperature prior to the measurement. The UV melting temperature profiles were recorded with a ramping and scanning rate of 1°C/min at 260 nm. All samples were measured at least three times. The *T*_m_ values from each measurement were calculated using the ‘first derivative’ method and presented as an average of three independent measurements. Thermodynamic data were obtained from van’t Hoff plots with five data points derived from a range of ODN concentrations (1−3 μM each).

### CD spectroscopy measurement

A solution (100 μl) containing equimolar amounts of ODNs (2 μM each), 10 mM sodium phosphate buffer and 150 mM NaCl at pH 7.0 was annealed as already described. The CD spectra were recorded at wavelengths between 220 and 400 nm at 20°C. All samples were measured at least three times.

### 2D-NMR analyses of the DNA duplex

The self-complementary ODN used for the NMR structural analysis (5′-TG^O^PzGGCC^N^PuCA) was prepared as already described. The counter ion of the phosphates was exchanged to Na^+^ by passing an aqueous solution of the ODN through the DOWEX HCR-S cation exchange resin (Na^+^ form) followed by dialysis of the eluent with the Cellu-Sep H1 membrane (MWCO: 2000) against ddH_2_O. The solution of ODN (1 mM as final concentration) in 10 mM sodium phosphate, 150 mM NaCl, 5% D_2_O, 0.05 mM DSS was filled into a D_2_O-matched 5 mm Shigemi NMR tube. The ^1^H 1D-NMR, NOESY and TOCSY spectra were measured by Bruker AVANCE III 600 MHz NMR spectrometer equipped with a 5 mm CryoProbe at 5°C. After a series of measurements, the samples were lyophilized and redissolved in D_2_O. The solution was then subjected to another set of ^1^H 1D-NMR, NOESY and TOCSY spectra measurements. The ^1^H–^1^H 2D NOESY spectra were measured with the pulse program ‘noesyesfpgpphrs’ for the H_2_O–D_2_O sample and ‘noesygpphpp’ for the D_2_O sample. The mixing time was 50 or 300 ms with the fid size of 4096 (F2) × 1024 (F1). The ^1^H–^1^H 2D TOCSY spectra were measured with the pulse program ‘mlevesgpph’ for the H_2_O–D_2_O sample and ‘mlevphpp’ for the D_2_O sample. The mixing time was 60 or 120 ms with the fid size of 4096 (F2) × 1024 (F1). All the spectra were processed and analyzed using Topspin 3.6.2.

### Molecular dynamics calculations

The simulations were carried out using the AMBER 18 program package (35). The initial structures of the modified DNA duplexes were built to be an Arnott B-DNA canonical structure using the AMBER NAB tool with the self-complementary sequence 5′-TGTGGCCACA. The underlined T and A were converted to ^O^Pz/^N^Pz and ^N^Pu/^O^Pu, respectively. The charges of the non-canonical modified residues were determined by the RESP charge fitting (HF/6–31G(d), iop(6/33 = 2)). For the AMBER force field, leaprc.DNA.OL15 and leaprc.water.tip3p were used. The additional force field parameters were taken from GAFF (gaff.dat) using the parmchk2 module (36). The duplexes were neutralized by Na^+^ and solvated in a periodic octahedral box with a 12 Å buffer of water molecules explicitly described by the TIP3P model. The initial minimizations were followed by heating to 300 K (Berendsen algorithm (37)) at a constant volume over a period of 100 ps using harmonic restraints of 25 kcal·mol^−1^·Å^−2^ on the atoms of the duplex. These restraints were gradually reduced (5, 4, 3, 2, 1 and 0.5 kcal·mol^−1^·Å^−2^) with equilibration (50 ps) under a constant temperature and pressure. Lastly, unrestrained simulations (1 ns) were performed for equilibration. The production simulations (100 ns) were then performed. During the MD simulations, hydrogen vibrations were removed using the SHAKE bond constraints, allowing a longer time step of 2 fs (38). Long range electrostatic interactions were treated using the Particle Mesh Ewald approach (39) and a 9 Å cutoff. A postprocessing analysis of the trajectories was carried out by the cpptraj module (v18.00) (40). Base pair and base step parameters were calculated using the nastruct command in the Cpptraj module with the calcnohb option and new reference files for each unnatural nucleoside. The coordination of reference positions of the purine derivatives (^N^Pu, ^O^Pu) and pyridazine derivatives (^O^Pz, ^N^Pz) were taken from the standard reference frames of adenine and thymine (41), respectively. See Supporting Information for the detailed calculation parameters.

## RESULTS AND DISCUSSION

### Synthesis of the alkynylated purine and pyridazine nucleosides

**Scheme 1.**
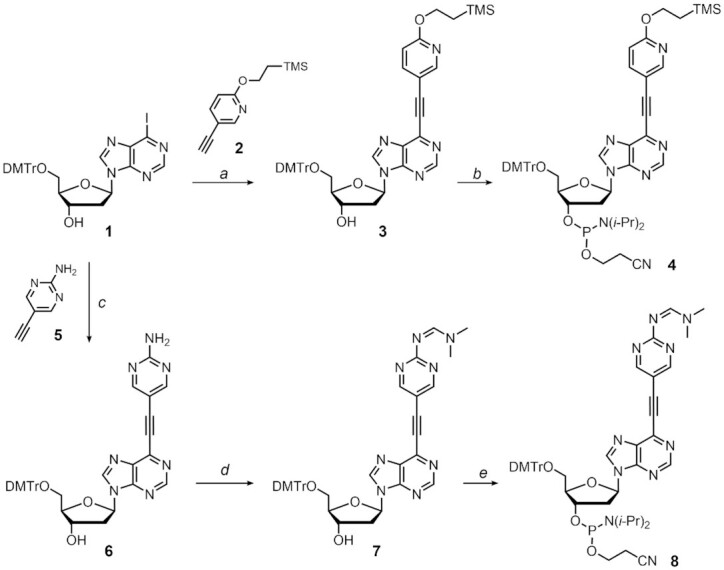
Synthesis of the phosphoramidites of ^O^Pu and ^N^Pu. Conditions: (a) **2**, Pd(PPh_3_)_2_Cl_2_, CuI, TEA, CH_3_CN, r.t., overnight, 77%; (b) Bis(2-cyanoethyl)-*N*,*N*-diisopropylphosphoramidite, *N*,*N*-diisopropylamino-tetrazolide, CH_3_CN, r.t., overnight, 68%; (c) **5**, Pd(PPh_3_)_2_Cl_2_, CuI, TEA, DMF, 40°C, 2.5 h, 72%; (d) *N*,*N*-Dimethylformamide dimethylacetal, MeOH, 40°C, 2 h, 72%; (e) Bis(2-cyanoethyl)-*N*,*N*-diisopropylphosphoramidite, *N*,*N*-diisopropylaminotetrazolide, CH_3_CN, r.t., overnight, 68%.

To investigate the base pairing properties of the alkynylated purine and pyridazine nucleosides, we started with the chemical syntheses of the phosphoramidite monomers of each nucleoside analogue for the solid-phase DNA synthesis. The syntheses of the alkynylated purine nucleosides are shown in Scheme 1 (see Supplementary Scheme S1 for further details). The Sonogashira coupling reaction of DMTr-protected iodopurine 2′-deoxyriboside **1** with 2-trimethylsilylethyl-protected ethynylpyridone **2** provided the 2-trimethylsilylethyl-protected ^O^Pu nucleoside **3**. This was subsequently converted into the phosphoramidite building block **4** for the DNA solid phase synthesis. Similarly, the coupling of compound **1** with ethynyl 2-aminopyrimidine **5** afforded the DMTr-protected ^N^Pu nucleoside **6**. Subsequent protection of the amino group as *N*,*N*-dimethylformamidine followed by phosphitylation of the 3′-OH group provided the corresponding phosphoramidite **8**.

**Scheme 2.**
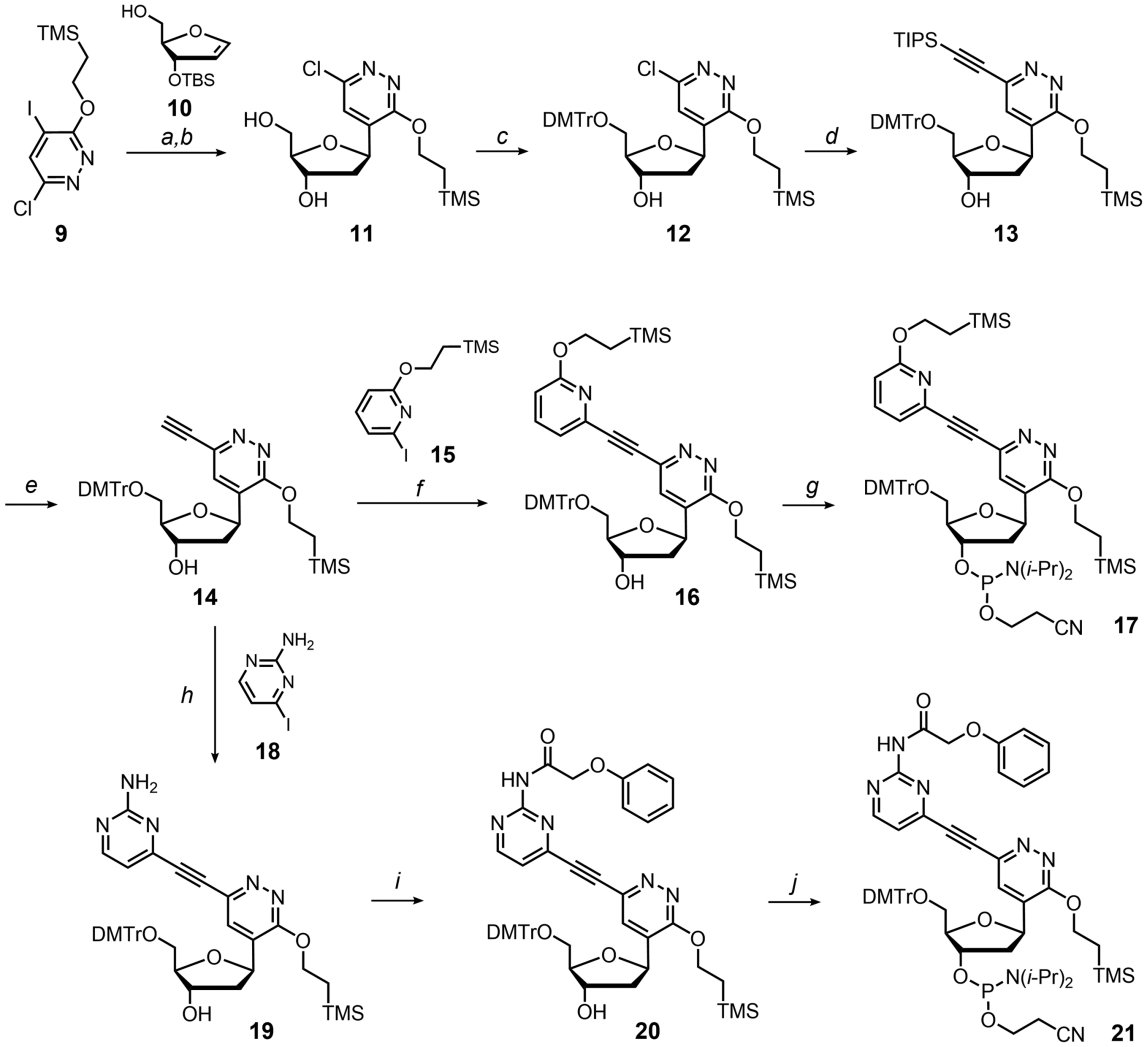
Synthesis of the phosphoramidites of ^O^Pz and ^N^Pz. Conditions: (a) **10**, Pd(OAc)_2_, Tris(pentafluorophenyl)-phosphine, Ag_2_CO_3_, CHCl_3_, 70°C, overnight; 3HF-TEA, THF, r.t., 1 h; (b) NaBH(OAc)_3_, AcOH, CH_3_CN, 0°C, 1 h, 66% over two steps; (c) DMTrCl, pyridine, 0°C to r.t., 1 h, 99%; (d) TIPS acetylene, XPhos, XPhos precatalyst^(43)^, Cs_2_CO_3_, CH_3_CN, 85°C, 4.5 h, 78%; (e) AgF, CH_3_CN, r.t., overnight, 78%; (f) **15**, Pd(PPh_3_)_2_Cl_2_, CuI, TEA, DMF, r.t., overnight, 83%; (g) bis(2-cyanoethyl)-*N*,*N*-diisopropylphosphor-amidite, *N*,*N*-diisopropylaminotetrazolide, CH_3_CN, r.t., overnight, 63%; h) **18**, Pd(PPh_3_)_2_Cl_2_, CuI, TEA, DMF, r.t., overnight, 84%; (i) TMSCl, pyridine, r.t., 40 min; pPhenoxyacetyl chloride, r.t., 2 h; 3HF-TEA, TEA, THF, r.t., 2 h, 58% over two steps; (j) Bis(2-cyanoethyl)-*N*,*N*-diisopropylphosphor-amidite, *N*,*N*-diisopropyl-aminotetrazolide, CH_3_CN, r.t., overnight, 74%.

The alkynylated pyridazine nucleosides were synthesized by the construction of the *C*-nucleoside skeleton as a key step (Scheme 2; see Supplementary Scheme S2 for further details). The 2-trimethylsilylethyl-protected 2-chloro-5-iodo-pyridazinone **9** was subjected to Mizoroki-Heck coupling with the TBS-protected glycal **10** under the conditions reported by Hocek *et al.* (42). Subsequent desilylation by triethylamine trihydrofluoride followed by the stereo-selective reduction of the carbonyl group with NaBH(OAc)_3_ afforded the 2-chloro-5-(2-trimethylsilyl)ethoxypyridazine nucleoside **11**. After protection of the 5′-OH group with a DMTr group, the protected nucleoside **12** was coupled with TIPS acetylene using the modified Sonogashira reaction (43). Notably, the conventional Sonogashira coupling conditions utilizing a palladium catalyst and copper (I) iodide provided only a trace amount of compound (13), presumably due to the low reactivity of the chloro-pyridazine. Subsequent desilylation with silver (I) fluoride provided the ethynyl pyridazine nucleoside **14** as a key intermediate. The compound **14** was then subjected to Sonogashira coupling with the 2-trimethylsilylethyl-protected iodopyridone **15** to provide the protected ^O^Pz nucleoside **16**, which was subsequently phosphitylated to afford the corresponding phosphoramidite **17**. Similarly, the coupling of the nucleoside **14** with 2-amino-4-iodopyrimidine **18** followed by phenoxyacetylation provided the protected ^N^Pz nucleoside **20**. The phenoxyacetyl was utilized instead of dimethylformamidine for protection of the NH_2_ group because the reaction of compound **19** with *N*,*N*-dimethylformamide dimethylacetal provided an unidentified polar compound. Finally, phosphitylation of the appropriately protected nucleoside **20** provided the phosphoramidite of ^N^Pz **21**.

In addition to the phosphoramidite monomers of each nucleoside, we also synthesized the fully-deprotected 2′-deoxyriboside of ^O^Pu, ^N^Pu, ^O^Pz and ^N^Pz (Supplementary Scheme S3). The UV absorption spectra of each nucleoside were then measured in H_2_O or buffered solutions at pH 7.0 (Supplementary Figures S1, S2). All the alkynylated purine and pyridazine nucleosides exhibited an extended UV absorption ranging to 380 nm, possibly due to the extended π system. Finally, the molar extinction coefficients at 260 nm were determined from the UV absorption spectra.

### Solid-phase synthesis of the ODNs containing the alkynylated purine and pyridazine nucleosides

**Figure 2.**
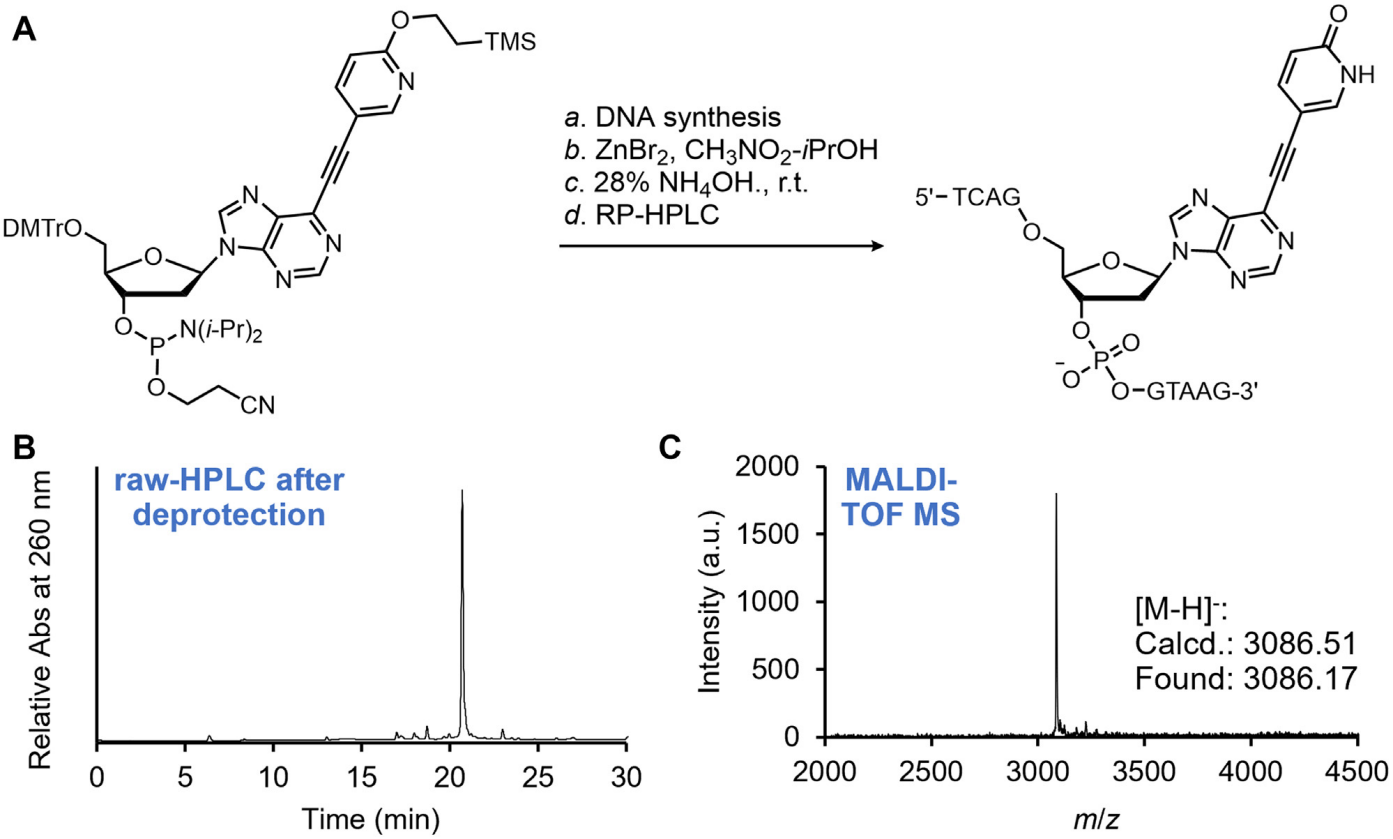
Synthesis and purification of the ^O^Pu-containing ODN. (**A**) A scheme of the solid phase DNA synthesis and deprotection. (**B**) RP-HPLC chart of the deprotection mixture. (**C**) MALDI-TOF MS spectrum of the isolated ^O^Pu-containing ODN.

All four alkynylated nucleosides were incorporated into the ODNs using a solid-phase DNA synthesizer. Preparation of the ^O^Pu-containing ODN is shown in Figure 2 as an example. After the DNA synthesis, the 2-trimethylsilylethyl group was deprotected by on-column treatment of the ODN with a solution of ZnBr_2_ in *i*PrOH-CH_3_NO_2_ (44). Subsequent deprotection of the other protecting groups and cleavage from the CPG resin were accomplished with 28% NH_4_OH at room temperature, and HPLC purification of the crude material provided the desired ODN. All the other ODNs containing the alkynylated nucleosides were prepared in the same manner, and their structural integrity and purity were confirmed by MALDI-TOF MS and HPLC analyses, respectively (Supplementary Table S1, Supplementary Figure S3).

During the deprotection, we noticed that the ^N^Pu- and ^N^Pz-containing ODNs provide an additional peak in the HPLC chart when the standard deprotection conditions of NH_4_OH at 55°C were utilized (Supplementary Figure S4). We speculated that this is due to the Michael addition of the NH_3_ molecule to the alkyne spacer of the ^N^Pu and ^N^Pz nucleosides. Fortunately, we found that such a side reaction can be suppressed to a negligible degree by conducting the deprotection with NH_4_OH at room temperature, and hence, all the ODNs were synthesized using the ‘ultra-mild’ conditions to assure compatibility with the deprotection conditions.

### The base pairing properties of the alkynylated purine and pyridazine nucleosides in DNA duplex

**Figure 3.**
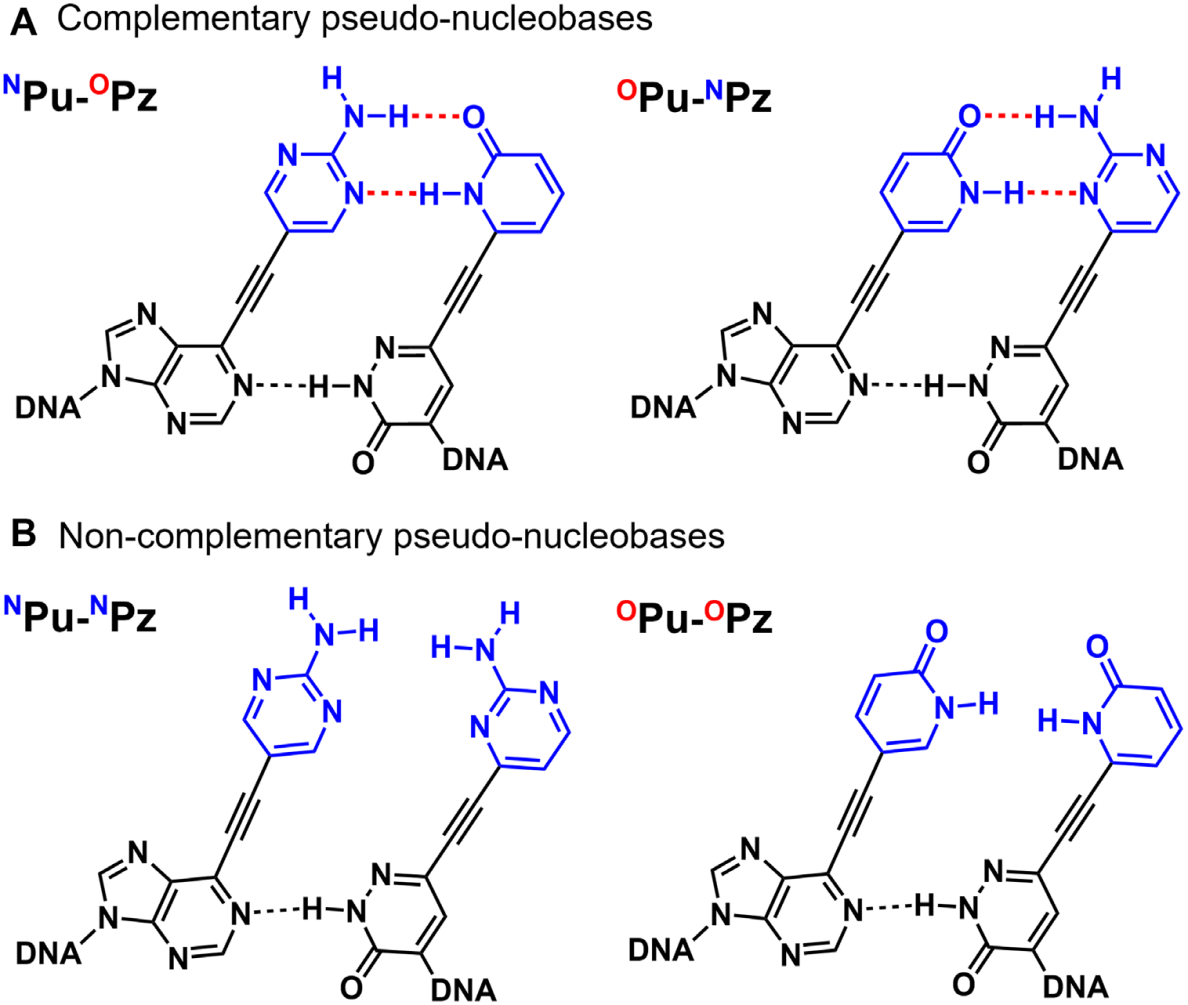
Plausible interaction modes between the alkynylated purine and pyridazine nucleosides in DNA duplex. (**A**) Interaction modes of ^N^Pu–^O^Pz and ^O^Pu–^N^Pz with complementary combination of pseudo-nucleobases. (**B**) Interaction modes of ^N^Pu–^N^Pz and ^O^Pu–^O^Pz with non-complementary combination of pseudo-nucleobases.

With the ODNs containing all four alkynylated purine and pyridazine nucleosides in hands, we evaluated their base pairing properties by comparing UV melting temperatures (*T*_m_) of DNA duplexes. The *T*_m_ values obtained from the 10-bp DNA duplexes featuring different combinations of the X–Y pairs are summarized in Table 1 (see Supplementary Figure S5 for the UV melting curves). The fully natural DNA duplexes with G–C and A–T pair at the position X–Y showed *T*_m_ values of 36.6°C and 31.8°C, respectively (Table 1, entries 1 and 2). In contrast, the *T*_m_ values of the duplexes featuring a ^N^Pu–^O^Pz and a ^O^Pu–^N^Pz pairs exhibited comparable or even higher thermal stabilities than the canonical pairs (Table 1, entries 3 and 4; *T*_m_ = 37.8°C and 36.1°C). On the other hand, ^N^Pu–^N^Pz and ^O^Pu–^O^Pz formed less stable base pairs in comparison to the ^N^Pu–^O^Pz and ^O^Pu–^N^Pz pairs (Table 1, entries 5 and 6; *T*_m_ = 30.4°C and 31.8°C). Similar pairing properties were reproduced with the DNA duplexes containing the inverted X–Y bases (Supplementary Table S2, entries 1–6). Since more stable pairing is observed when the combination of the pseudo-nucleobases is the complementary 2-aminopyrimidine and 2-pyridone (^N^Pu–^O^Pz and ^O^Pu–^N^Pz) than with the non-complementary sets (^N^Pu–^N^Pz and ^O^Pu–^O^Pz), the results indicated that the hydrogen-bonding complementarity between the pseudo-nucleobases could be one of the key factors for the selectivity and thermal stability of these unnatural base pairs (Figure 3).

**Table 1.** *T*_m_ values of the DNA duplexes featuring different combinations of ^N^Pu, ^O^Pu, ^N^Pz, ^O^Pz and canonical bases

Entry	Duplex sequences	Base pair	*T*_m_ (°C)^a^	Δ*T*_m_ (°C)
1	5′-TCAG **G** GTAAG 3′-AGTC **C** CATTC	G–C	36.6 ± 0.3	-
2	5′-TCAG **A** GTAAG 3′-AGTC **T** CATTC	A–T	31.8 ± 0.3	-
3	5′-TCAG **^N^Pu** GTAAG 3′-AGTC **^O^Pz** CATTC	^N^Pu–^O^Pz	37.8 ± 0.1	-
4	5′-TCAG **^O^Pu** GTAAG 3′-AGTC **^N^Pz** CATTC	^O^Pu–^N^Pz	36.1 ± 0.6	-
5	5′-TCAG **^N^Pu** GTAAG 3′-AGTC **^N^Pz** CATTC	^N^Pu–^N^Pz	30.4 ± 0.7	-
6	5′-TCAG **^O^Pu** GTAAG 3′-AGTC **^O^Pz** CATTC	^O^Pu–^O^Pz	31.8 ± 0.3	-
7	5′-TCAG **^N^Pu** GTAAG 3′-AGTC **A** CATTC	^N^Pu–A	21.7 ± 0.6	–16.1^b^
8	5′-TCAG **^N^Pu** GTAAG 3′-AGTC **G** CATTC	^N^Pu–G	23.0 ± 0.6	–14.8^b^
9	5′-TCAG **^N^Pu** GTAAG 3′-AGTC **T** CATTC	^N^Pu–T	22.0 ± 0.4	–15.8^b^
10	5′-TCAG **^N^Pu** GTAAG 3′-AGTC **C** CATTC	^N^Pu–C	24.7 ± 0.5	–13.1^b^
11	5′-TCAG **^O^Pu** GTAAG 3′-AGTC **A** CATTC	^O^Pu–A	22.9 ± 0.1	–13.2^c^
12	5′-TCAG **^O^Pu** GTAAG 3′-AGTC **G** CATTC	^O^Pu–G	23.6 ± 0.3	–12.5^c^
13	5′-TCAG **^O^Pu** GTAAG 3′-AGTC **T** CATTC	^O^Pu–T	22.6 ± 0.3	–13.5^c^
14	5′-TCAG **^O^Pu** GTAAG 3′-AGTC **C** CATTC	^O^Pu–C	22.6 ± 0.4	–13.5^c^
15	5′-TCAG **A** GTAAG 3′-AGTC **^N^Pz** CATTC	A–^N^Pz	28.2 ± 0.6	–7.9^c^
16	5′-TCAG **G** GTAAG 3′-AGTC **^N^Pz** CATTC	G–^N^Pz	22.7 ± 0.4	–13.4^c^
17	5′-TCAG **T** GTAAG 3′-AGTC **^N^Pz** CATTC	T–^N^Pz	17.3 ± 0.8	–18.8^c^
18	5′-TCAG **C** GTAAG 3′-AGTC **^N^Pz** CATTC	C–^N^Pz	21.1 ± 0.1	–15.0^c^
19	5′-TCAG **A** GTAAG 3′-AGTC **^O^Pz** CATTC	A–^O^Pz	29.5 ± 0.2	–8.3^b^
20	5′-TCAG **G** GTAAG 3′-AGTC **^O^Pz** CATTC	G–^O^Pz	21.9 ± 0.6	–15.9^b^
21	5′-TCAG **T** GTAAG 3′-AGTC **^O^Pz** CATTC	T–^O^Pz	17.7 ± 1.2	–20.1^b^
22	5′-TCAG **C** GTAAG 3′-AGTC **^O^Pz** CATTC	C–^O^Pz	18.5 ± 1.3	–19.3^b^

^a^Conditions: 2 μM of each ODN, 150 mM NaCl, 10 mM sodium phosphate buffer, pH 7.0. Each *T*_m_ value is average of three measurements. ^b^To the duplex with a ^N^Pu–^O^Pz pair (entry 2). ^c^To the duplex with a ^O^Pu–^N^Pz pair (entry 3).

To better understand the base pairing properties, the thermodynamic parameters were determined for the DNA duplexes containing G–C, ^N^Pu–^O^Pz, ^O^Pu–^N^Pz, ^N^Pu–^N^Pz and ^O^Pu–^O^Pz (Supplementary Figure S6, Supplementary Table S3). In comparison to the entropy change associated with formation of a G–C pair, a significantly smaller unfavorable entropy change was observed for the ^N^Pu–^O^Pz and ^O^Pu–^N^Pz pairs, whereas the free energy changes of these base pairs were comparable (Supplementary Table S3, entries 1–3). This could be attributed to the local preorganization of the single-stranded ODNs through the stacking interaction of the ethynyl-linked pseudo-nucleobases with adjacent bases. Such a trend was previously observed with 5-propynylated pyrimidine nucleoside analogs (45), which have been shown to increase the thermal stability of the duplex by a preorganizing effect. In line with this, the ethynyl moiety of the alkynylated purine and pyridazine nucleoside may also provide a thermal stability to the duplex through an enhanced stacking interaction with the adjacent base pairs. The data further demonstrated a more favorable enthalpy change of the complementary ^N^Pu–^O^Pz and ^O^Pu–^N^Pz pairs compared to the non-complementary ^N^Pu–^N^Pz and ^O^Pu–^O^Pz pairs (Supplementary Table S3, entries 2–4); this may be ascribed to the formation of stable hydrogen bonds between the complementary pseudo-nucleobases, suggesting the presence of a hydrogen-bond driven recognition in the DNA major groove.

We then turned our attention to the base pairing properties of the alkynylated purines and pyridazines against the natural nucleosides (Table 1, entries 7–22). Both ^N^Pu and ^O^Pu with the purine core structure proved to be highly selective against pairing with A, G, C and T as indicated by the significantly reduced *T*_m_ values (Table 1, entries 7–14) compared to the matched ^N^Pu–^O^Pz and ^O^Pu–^N^Pz pairs (Table 1, entries 3 and 4). Although ^N^Pu and ^O^Pu can potentially form a hydrogen bond with the N-H group of T at the N-1 position of the purine cores, a good selectivity against pairing with T was observed (Table 1, entries 9 and 13). This result can be explained by the steric repulsion of the carbonyl group of thymine with the pseudo-nucleobases of the alkynylated purine derivatives (Supplementary Figure S7a). ^N^Pz and ^O^Pz with the pyridazine core structures also exhibited a selectivity against the canonical nucleobases (Table 1, entries 15–22) though these nucleosides showed modest stability toward A as well (Table 1, entries 15 and 19). This is presumably due to the formation of single hydrogen bond between the NH group of the pyridazine core and N-1 of adenine base (Supplementary Figure S7b). Nevertheless, ^N^Pz and ^O^Pz exhibited the significantly higher stability toward pairing with complementary ^O^Pu and ^N^Pu, respectively. The results were also consistent in case the X-Y position was inverted (Supplementary Table S2, entries 7–22), thus confirming the selective pairing properties of the alkynylated purine and pyridazine nucleosides.

### Structural impact of the alkynylated purine-pyridazine pairs in DNA duplex

To assess the structural impact of the alkynylated purine-pyridazine base pairs, we performed the CD spectroscopy measurement of the 10-bp DNAs (Supplementary Figure S8). The CD spectra of the fully natural DNA duplexes were consistent with the formation of a typical B-type structure with a positive CD band around 270 nm and a negative band around 250 nm. In line with this, the duplex DNAs containing the ^N^Pu–^O^Pz and ^O^Pu–^N^Pz pairs exhibited similar CD spectra. As the shapes of the spectra obtained with inversed bases at the X–Y position (X–Y = ^O^Pz–^N^Pu and ^N^Pz–^O^Pu) showed a similarity, the flanking seems to have limited influence on the conformation of the DNA duplexes in the present sequence context. Overall, the results demonstrated that the alkynylated purine-pyridazine pairs are well-accommodated in the B-type DNA structure.

### 2D-NMR analyses of the DNA duplex containing ^N^Pu–^O^Pz pairs

**Figure 4.**
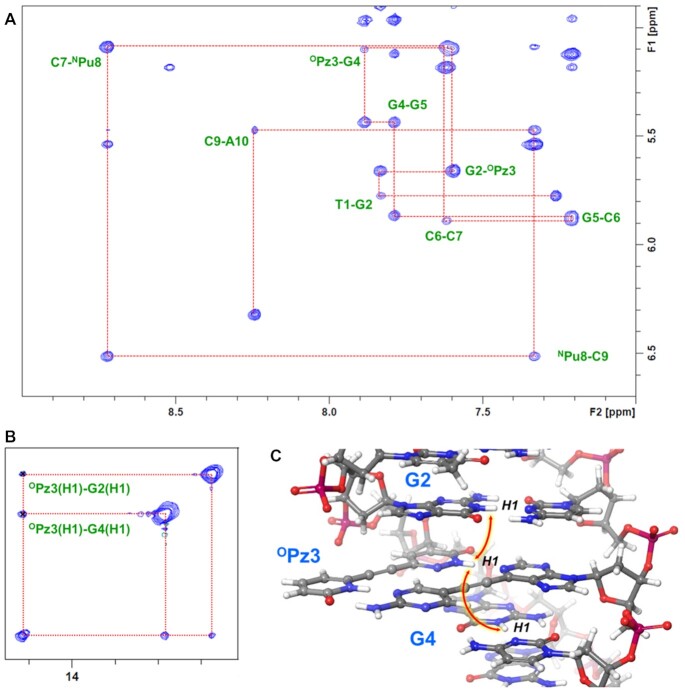
NMR analysis of ^N^Pu–^O^Pz pair in the 10-bp DNA duplex. (**A**) An excerpt of the NOESY spectrum showing the connectivity of H1′ and base protons through the strand. The spectrum was measured in D_2_O at 5°C. (**B**) An excerpt from the NOESY spectrum showing the inter-base cross-peaks between the H1 imino proton of ^O^Pz and the H1 protons of G2 and G4. The spectrum was measured at H_2_O–D_2_O at 5°C. (**C**) A depiction of the NOEs observed between the imino protons of G2, ^O^Pz3 and G4. The model structure was built using MacroModel.

To elucidate the recognition mode of the alkynylated purine-pyridazine base pair, we performed the 2D-NMR analysis of a self-complementary DNA duplex (10 bp; 5′-TG^O^PzGGCC^N^PuCA) within which is designed to form ^N^Pu–^O^Pz base pairs. The NOESY and TOCSY spectra were measured with a buffered solution in D_2_O and 5% D_2_O in H_2_O (Supplementary Figure S9–S12). In the NOESY spectrum, the evident NOE connectivity of the base protons to H1′ (Figure 4A) as well as to H2′/H2′ (Supplementary Figure S13) through the strand was confirmed, indicating the continuous alignment of the stacked nucleobases within the duplex structure. Furthermore, the NOE connectivity analysis of the imino-protons revealed the formation of a hydrogen bond between the purine and the pyridazine rings of the ^N^Pu–^O^Pz pair; a highly-deshielded proton at 14.57 ppm showed a significant NOE between the H1-protons of G2 and G4 (Figure 4B), which was assigned as the hydrogen-bonded H1 proton of the pyridazine ring. These data indicated that the purine and pyridazine core structures of the ^N^Pu–^O^Pz pairs maintain a stacking continuity with the adjacent base pairs in the double helix (Figure 4C).

**Figure 5.**
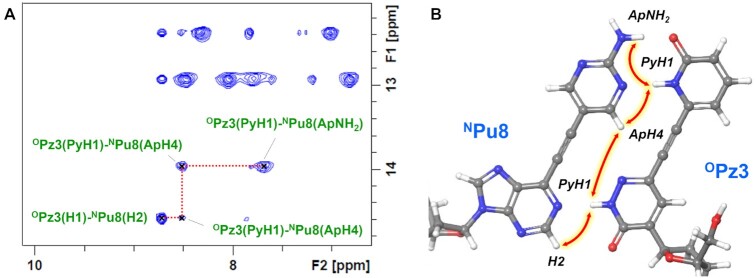
(**A**) An excerpt from NOESY spectrum showing the NOE connections between the protons located at the interface of ^N^Pu-–^O^Pz pair. (**B**) A depiction of the NOEs observed at the interface of the ^N^Pu–^O^Pz pair. The model structures were built using MacroModel.

We then analyzed the structure around the pseudo-nucleobases. Initially, the proton peaks of the pseudo-nucleobases were assigned. A careful observation of the structure led us to notice the structural vicinity of the 2-pyridone with a sugar moiety of G2 as well as proximal positioning of the 2-aminopyrimidine moiety and cytosine ring of the adjacent C7. Indeed, two proton signals in the aromatic region exhibited distinctive cross-peaks with G2(H2′,2′) and C7(H5), respectively, and each of them was assigned as ApH4 on the 2-aminopyrimidine moiety of ^N^Pu8 and PyH5 on the 2-pyridone ring of ^O^Pz3 (Supplementary Figure S14). Further NOESY and TOCSY spectra analyses enabled assignment of the remaining proton peaks from the 2-pyridone ring of ^O^Pz3 as well (Supplementary Figure S15). Subsequently, the hydrogen-bond formation between the pseudo-nucleobases was clarified by analyzing the NOESY spectra. Two significant cross-peaks, ^O^Pz3(PyH1)–^N^Pu8(ApH4) and ^O^Pz3(PyH1)–^N^Pu8(ApNH2), associated with the highly-deshielded PyH1 proton strongly suggested the formation of hydrogen bonds between the 2-pyridone and 2-aminopyrimidine moieties (Figure 5A). In addition, the evident extension of the NOE connection with PyH1 of ^O^Pz3 and H2 of ^N^Pu8 further supported the formation of a hydrogen bond between the purine and pyridazine core structure. Taken together, these results indicated that ^N^Pu–^O^Pz pair adopts the recognition structure as designed (Figure 5B).

### MD calculation of the DNA duplexes containing the alkynylated purine and pyridazine nucleosides

**Figure 6.**
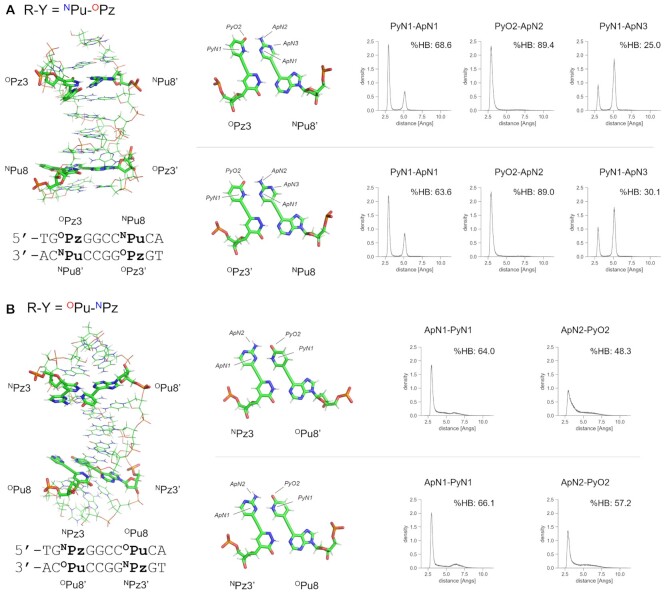
Energy minimized structures of the last snapshot from the MD calculations of the DNA duplexes featuring (**A**) ^N^Pu–^O^Pz and (**B**) ^O^Pu–^N^Pz. The MD calculation was performed using AMBER force field with TIP3P water model for 100 ns. The distances between the hydrogen-bonding heteroatoms on the pseudo-nucleobases are shown in histograms. %HB denotes the percentage of frames showing less than 3.5 Å between the heteroatoms in 100 ns simulation.

To gain further insight into the recognition mode as well as the structural impact of the alkynylated purine–pyridazine base pairs in the duplex structure, we performed an MD simulation of the self-complementary DNA duplexes featuring ^N^Pu–^O^Pz, ^O^Pu–^N^Pz, ^N^Pu–^N^Pz and ^O^Pu–^O^Pz. The calculation was performed using the AMBER force field with the TIP3P water model for 100 ns (see Supplementary Figures S16–S18 for the detailed parameters). All four DNAs containing different combinations of the alkynylated purine-pyridazine pairs retained the duplex structures throughout the calculation as indicated by the low deviation from the initial structures (Supplementary Figure S19). For the DNA duplex containing the ^N^Pu–^O^Pz pair, the simulation clearly indicated the formation of hydrogen bonds between the pseudo-nucleobases (Figure 6A); the distances between the respective heteroatoms of 2-aminopyrimidine and 2-pyridone were predominantly maintained within the range of a hydrogen bond during the 100 ns simulation. Similarly, the complementary ^O^Pu–^N^Pz pair in the DNA duplex also confirmed the steady formation of hydrogen bonds between the pseudo-nucleobases (Figure 6B). On the other hand, the results of ^N^Pu–^N^Pz and ^O^Pu–^O^Pz in the DNA duplex indicated that the formation of the hydrogen bonds is disfavored with the non-complementary combinations of the pseudo-nucleobases (Supplementary Figure S20a, b). Together with the structural data obtained from the 2D-NMR analysis, these calculation results support the validity of the design strategy based on the formation of the in-major-groove hydrogen bonds. It is interesting that such hydrogen-bonding takes place in the hydrophilic major groove. This could be attributed to the proximal positioning of the pseudo-nucleobases *via* the rigid alkyne spacer.

We also investigated the detailed structural features of the DNA duplexes containing the alkynylated purine-pyridazine pairs. To this end, the base pair and base step parameters were calculated and compared tp those of an A–T pair-containing duplex (Supplementary Figures S21 and S22, Supplementary Table S4). Although slight differences were observed, the calculated parameters suggested that the incorporation of the alkynylated purine-pyridazine pairs does not significantly alter the overall duplex structure.

### Duplex formation of the ODNs containing multiple alkynylated purine and pyridazine nucleosides

Finally, we investigated the thermal stability of the DNA duplexes containing multiple alkynylated purine–pyridazine pairs to assess the generality of our newly designed UBPs. When two or three non-contiguous positions were substituted with ^N^Pu–^O^Pz pair, these duplexes exhibited higher *T*_m_ values than the corresponding duplexes with the canonical A–T pairs (Table 2, Supplementary Figure S23). On the contrary, a significant decrease in the *T*_m_ values was observed for the corresponding duplexes containing ^O^Pu–^O^Pz or A–^O^Pz in which the hydrogen-bonding pattern of the pseudo-nucleobases was non-complementary or either the pseudo-nucleobase was absent. The results indicated that the in-major-groove recognition functions even in the case with the multiple substitutions in the DNA duplex, showcasing the robustness of the base pairing ability of the alkynylated purine-pyridazine base pairs. We further investigated the structural profiles of the duplexes containing multiple ^N^Pu–^O^Pz pairs by CD measurement (Supplementary Figure S24). The spectra showed additional signals in the 300–400 nm range, which indicated the presence of exciton couplings between the pairs of the pseudo-nucleobases in the major groove. At the same time, we noticed the change of the signal shape in the canonical nucleobase region (240–280 nm) upon increasing the number of ^N^Pu–^O^Pz pairs. Such a change in the spectra could be attributed to the different stacking mode or hydrophobic interaction exhibited by the ethynyl-linked pseudo-nucleobases in the major groove, as previously reported (46).

**Table 2.** *T*_m_ values of the DNA duplexes featuring multiple alkynylated purine and pyridazine nucleosides

	*T*_m_ (Δ*T*_m_) values for each X–Y combination^b^			
Duplex sequence^a^	A–T	^N^Pu–^O^Pz	^O^Pu–^O^Pz	A–^O^Pz
5′-TCA**X**G**X**TAAG-3′ 3′-AGT**Y**C**Y**ATTC-5′	26.0 ± 0.2	31.8 ± 0.3	14.9 ± 0.9	14.0 ± 1.1
5′-CTT**X**C**X**CTGA-3′ 3′-GAA**Y**G**Y**GACT-5′	30.6 ± 0.4	44.2 ± 0.5	31.3 ± 0.2	26.5 ± 0.4
5′-TCA**X**G**X**T**X**AG-3′ 3′-AGT**Y**C**Y**A**Y**TC-5′	26.0 ± 0.2	40.6 ± 0.5	16.0 ± 0.7	n.d.
5′-CTT**X**C**X**C**X**GA-3′ 3′-GAA**Y**G**Y**G**Y**CT-5′	32.7 ± 0.1	46.6 ± 0.2	31.8 ± 0.4	23.9 ± 0.9

^a^Conditions: 2 μM of each ODN, 150 mM NaCl, 10 mM sodium phosphate buffer, pH 7.0. Each *T*_m_ value is average of three measurements. n.d.: not determined.

We then performed the *T*_m_ measurement of the duplex DNAs featuring consecutive ^N^Pu–^O^Pz pairs in the middle of the sequence. The results revealed that the tandem incorporation of ^N^Pu–^O^Pz pairs dramatically stabilize the duplex DNA (Table 3, Supplementary Figure S25); in comparison with the duplex featuring the single ^N^Pu–^O^Pz pair, two and three contiguous substitutions boosted the *T*_m_ values by +7.9°C and +26.0°C, respectively. In contrast, a significant destabilization was observed for the duplexes containing the ^O^Pu–^O^Pz or A–^O^Pz pairs, in which the hydrogen-bonding pattern of the pseudo-nucleobases was non-complementary or either the pseudo-nucleobase was absent. The individual single stranded ODNs did not exhibit the typical sigmoidal hyperchromicity upon heating (Supplementary Figure S26), confirming that the transitions of the UV absorbance are indeed attributed to the duplex-to-single strand dissociation. Such stabilization was again reproduced with the duplex DNAs containing the inverted ^N^Pu–^O^Pz, ^O^Pu–^O^Pz and A–^O^Pz pairs at the same positions (Supplementary Figures S27 and S28, Supplementary Table S5). These results point to the enhancement of the thermal stability due to the cooperative stacking between the planar, hydrogen-bonded pseudo-nucleobases.

**Table 3.** *T*_m_ values of the DNA duplexes featuring consecutive alkynylated purine and pyridazine nucleosides

	*T*_m_ (Δ*T*_m_) values for each X–Y combination^a^		
Duplex sequence	^N^Pu–^O^Pz	^O^Pu–^O^Pz	A–^O^Pz
5′-TCAG**X**GTAAG-3′ 3′-AGTC**Y**CATTC-5′	37.8 ± 0.1	31.8 ± 0.3	29.5 ± 0.2
5′-TCAG**XX**TAAG-3′ 3′-AGTC**YY**ATTC-5′	45.7 ± 0.3 (+7.9)^b^	18.7 ± 0.3 (–13.1)^c^	20.1 ± 0.2 (–9.4)^d^
5′-TCA**XXX**TAAG-3′ 3′-AGT**YYY**ATTC-5′	63.8 ± 0.4 (+26.0)^b^	15.7 ± 0.6 (–16.1)^c^	n.d. (n.d.)

^a^Conditions: 2 μM of each ODN, 150 mM NaCl, 10 mM sodium phosphate buffer, pH 7.0. Each *T*_m_ value is average of three measurements. n.d.: not determined. Δ*T*_m_ values are shown in parentheses. ^b^To the duplex with single ^N^Pu–^O^Pz pair. ^c^To the duplex with single ^O^Pu–^O^Pz pair. ^d^To the duplex with single A–^O^Pz pair.

**Figure 7.**
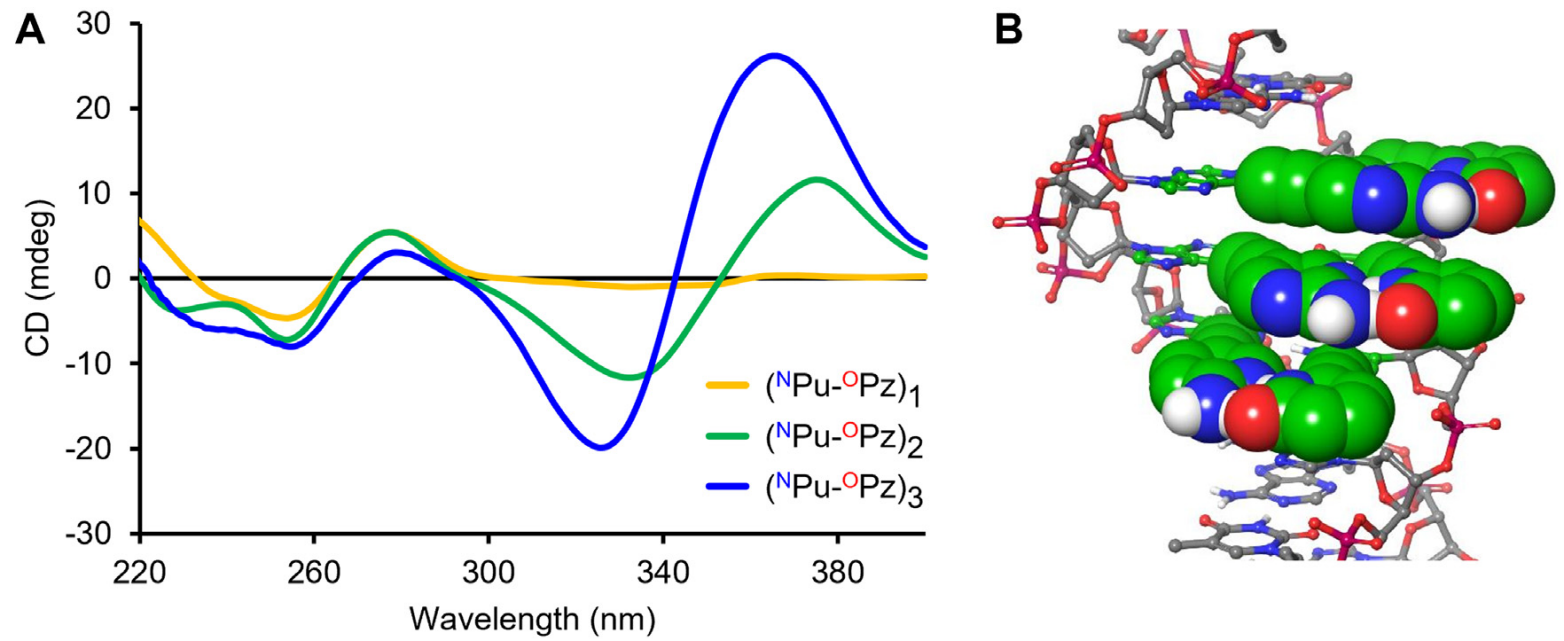
(**A**) CD spectra of the DNA duplexes featuring consecutive ^N^Pu-^O^Pz pairs. Conditions: 2 μM of each ODN, 150 mM NaCl, 10 mM sodium phosphate buffer, pH 7.0 at 20°C. (**B**) A model structure depicting the stacked pseudo-nucleobases (shown as CPK model) in the major groove of the DNA duplex. The model was built using MacroModel.

To elucidate the origin of the dramatic stabilization effect, the thermodynamic parameters of the duplex DNAs containing two or three consecutive ^N^Pu–^O^Pz pairs were derived from the van’t Hoff analysis of the UV melting curves (Supplementary Figure S29, Supplementary Table S6). The analysis revealed that the more stable duplex formation with two or three consecutive ^N^Pu–^O^Pz pairs is accompanied by significantly reduced entropy losses, suggesting the enhanced preorganization degree of the ODN in the single-stranded state. Furthermore, the CD spectra of the duplex DNAs containing two or three ^N^Pu–^O^Pz pairs exhibited a strong exciton coupling at 300–400 nm with their spectral shapes being similar to that of the canonical nucleobase region in 240–280 nm (Figure 7A, Supplementary Figure S30). This suggests that the pseudo-nucleobases are aligned along the helical geometry and thereby participating in the consecutive stacking interaction (Figure 7B) (47,48). Collectively, the results demonstrated that the consecutive incorporation of alkynylated purine-pyridazine base pairs can enhance the stability of the duplex DNA while maintaining the hybridization specificity.

## CONCLUSION

In conclusion, we have successfully demonstrated a selective and stable base pairing by the alkynylated purine-pyridazine nucleosides under the concept of isolating the recognition interface. The UV melting temperature study as well as the 2D-NMR analysis and MD simulation have shown that the selective pairing is driven by the formation of a base-pair-like structure in the major groove. In addition, we discovered that the consecutive incorporation of the hydrogen-bonded ^N^Pu–^O^Pz pair dramatically stabilized the DNA duplexes. To the best of our knowledge, our UBP is the first example for achieving the cooperative stabilization of the duplex structure while maintaining a high pairing selectivity against pairing with the natural nucleosides. Notably, our design concept can be, in principle, adopted with molecular interactions other than hydrogen bonding, e.g. a hydrophobic interaction and metal ion coordination. Thus, we expect that the present approach would pave a way for a development of new type of UBPs as well as functional modified nucleosides.

The high selectivity and thermal stability of the alkynylated purine-pyridazine pairs in the duplex structures make it promising for applications in hybridization-based DNA nanotechnologies with an enhanced fidelity (17) as well as construction of structurally diversified DNA architectures by expanding the pairing components (49). The utility of consecutive ^N^Pu–^O^Pz pairs may allow the thermal stabilization and shortening of the duplex structures found in the functional oligonucleotides without compromising their activity (50,51). Aside from the exploration of hybridization-based applications, the research is also ongoing toward the DNA polymerase-mediated replication of the alkynylated purine-pyridazine base pairs. Such an enzymatic replication study would provide not only a deeper insight into the structural basis for the selectivity of the alkynylated purine-pyridazine pairs against the canonical base pairs but would also create an important foundation toward the wider utility of our UBPs, as exemplified by development of functional DNA aptamers (52,53) as well as expansion of the genetic code (54,55).

## DATA AVAILABILITY

All data are available in the main article and in Supplementary Information.

## Supplementary Material

gkac140_Supplemental_File
