# Supplementary material for: Selective and stable base pairing by alkynylated nucleosides featuring a spatially-separated recognition interface
Source: Nucleic Acids Res. 2022 Mar 2;50(6):3042–55. doi: 10.1093/nar/gkac140 (PMC8989583; doi:10.1093/nar/gkac140)
Supplement: gkac140_Supplemental_File [file gkac140_supplemental_file.docx]

Supporting information

**Selective and stable base pairing by alkynylated nucleosides**

**featuring a spatially-separated recognition interface**

Hidenori Okamura,^a,b,^* Giang Hoang Trinh, ^a,b^ Zhuoxin Dong,^a,b^ Yoshiaki Masaki,^c^

Kohji Seio,^c^ Fumi Nagatsugi^a,b,^*

^a^Institute of Multidisciplinary Research for Advanced Materials, Tohoku University, 2-1-1 Katahira, Aoba-ku, Sendai, Miyagi 980-8577, Japan

^b^Department of Chemistry, Graduate School of Science, Tohoku University, Aoba-ku, Sendai, Miyagi 980-8578, Japan

^c^Department of Life Science and Technology, Tokyo Institute of Technology, 4259 Nagatsuta-cho, Midori-ku, Yokohama, Kanagawa 226 8501, Japan

E-mail: hidenori.okamura.b8@tohoku.ac.jp, fumi.nagatsugi.b8@tohoku.ac.jp

1. Synthesis of the nucleoside derivatives2

[2. UV absorption spectra of the alkynylated purine and pyridazine nucleosides](#_Toc347994870) 21

[3. Sequences and characterization of the synthesized oligonucleotides](#_Toc347994870) 23

[4. UV melting analyses of the duplexes containing the alkynylated purines and pyridazines at single position](#_Toc347994875) 28

[5. Thermodynamic analyses of the duplexes containing single alkynylated purine-pyridazine pair](#_Toc347994875) 32

[6. Speculated interaction modes of the alkynylated purines and pyridazines with canonical bases](#_Toc347994875) 33

[7. CD spectra of the duplexes containing single alkynylated purine-pyridazine pairs](#_Toc347994875) 34

[8. NMR structural studies of the duplex containing ^N^Pu-^O^Pz pairs](#_Toc347994875) 35

[9. MD simulations of the duplexes containing the alkynylated purine-pyridazine pairs](#_Toc347994875) 39

[10. UV melting analyses of the duplexes containing the alkynylated purines and pyridazines at multiple position](#_Toc347994875) 55

[11. CD spectra of the duplexes containing multiple alkynylated purine-pyridazine pairseferences](#_Toc347994875) 56

[12. UV melting analyses of the duplexes containing the alkynylated purines and pyridazines at consecutive positionrs](#_Toc347994875) 57

[13. Thermodynamic analyses of the duplexes containing consecutive alkynylated purine-pyridazine pair](#_Toc347994875) 60

[14. CD spectra of the duplexes containing consecutive alkynylated purine-pyridazine pairs](#_Toc347994875) 61

[15. NMR spectra of the synthesized compounds](#_Toc347994875) 62

[16. References](#_Toc347994875) 98

**1.** **Synthesis of the nucleoside derivatives**

**Scheme S1**. Full synthesis scheme for ^O^Pu and ^N^Pu phosphoramidites.

**2'-Deoxy-3',5'-di-*O*-acetyladenosine (S1)**


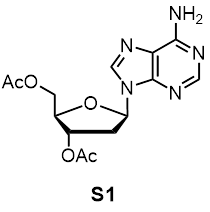
To a suspension of 2’-deoxyadenosine (4.0 g, 15.9 mmol) in CH_3_CN (100 ml) was added DMAP (156 mg, 1.28 mmol), triethylamine (6.0 ml, 10.5 mmol) and acetic anhydride (3.6 ml, 38.4 mmol), and the reaction mixture was stirred for 1 h at room temperature. The reaction was quenched by adding MeOH, and the mixture was concentrated under reduced pressure. The residue was suspended in CH_2_Cl_2_ and washed with water followed by brine. The organic layer was dried over Na_2_SO_4_ and evaporated under reduce pressure. The residue was re-dissolved in a small volume of CH_2_Cl_2_ and added dropwise to a mixture of hexane-Et_2_O (1:1, 400 ml). The white precipitate was filtered and washed with hexane to afford compound **S1** (94%, 4.9 g, 3.57 mmol) as a white powder.

^1^H NMR (400 MHz, CDCl_3_) δ 8.36 (1H, s), 7.98 (1H, s), 6.43 (1H, dd, *J* = 6.0, 8.0 Hz), 5.68 (2H, brs), 5.43 (dt, 1H, *J =* 2.4, 6.4 Hz), 4.44-4.33 (3H, m), 2.95 (1H, ddd, *J =* 6.4, 8.0, 14.0 Hz), 2.62 (1H, ddd, *J* = 2.4, 6.0, 14.0 Hz), 2.13 (3H, s), 2.09 (3H, s); ^13^C NMR (151 MHz, CDCl_3_) δ 170.6, 170.5, 155.6, 153.3, 149.8, 138.7, 120.3, 84.7, 82.7, 74.7, 63.9, 37.7, 21.1, 20.9; HRMS (ESI-TOF) calcd. for C_14_H_18_N_5_O_5_^+^ [M+H]^+^: 336.1302, found: 336.1308.

**2'-Deoxy-3',5'-di-*O*-acetyl-6-iodo-*β*-D-purineriboside (S2)**


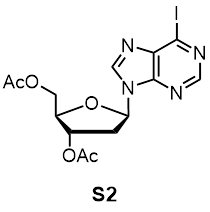
A suspension of compound **S1** (4.0 g, 11.9 mmol) in isoamylnitrite (31 ml, 226 mmol) and diiodomethane (31 ml, 357 mmol) was stirred at 60 °C for 2.5 h. The reaction mixture was concentrated under reduced pressure, and the residue was taken up in CH_2_Cl_2_. The mixture was washed with saturated aqueous solution of Na_2_S_2_O_3_, brine, dried over Na_2_SO_4_ and evaporated under reduced pressure. The residue was purified by silica gel column chromatography (CH_2_Cl_2_:Hexane = 4:1 then CH_2_Cl_2_:CH_3_OH = 100:0 to 200:1 to 150:1) to afford compound **S2** (49%, 2.6 g, 5.83 mmol) as a pale yellow powder.

^1^H NMR (400 MHz, CDCl_3_) δ 8.64 (1H, s), 8.32 (1H, s), 6.47 (1H, dd, *J* = 6.0, 7.6 Hz), 5.45-5.44 (1H, m), 4.39-4.35 (3H, m), 2.99 (1H, ddd, *J* = 6.4, 7.6, 14.0 Hz), 2.68 (1H, ddd, *J* = 2.8, 6.0, 14.0 Hz), 2.15 (3H, s), 2.09 (3H, s); ^13^C NMR (151 MHz, CDCl_3_) δ 170.4, 170.4, 152.2, 147.5, 142.8, 139.4, 122.6, 85.3, 83.0, 63.7, 37.8, 21.0, 20.9; HRMS (ESI-TOF) calcd. for C_14_H_16_IN_4_O_5_^+^ [M+H]^+^: 447.0160, found: 447.0155.

**2'-Deoxy-6-iodo-*β*-D-purineriboside (S3)**


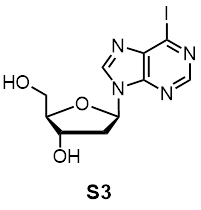
A solution of compound **S2** (3.6 g, 8.07 mmol) in methanolic ammonia (2.0 M, 300 ml) was kept overnight at 4 °C. The reaction mixture was concentrated under reduced pressure and was co-evaporated with EtOH. The residue was recrystallized in hot EtOH to afford compound **S3** (78%, 2.29 g, 6.33 mmol) as a white solid.

^1^H NMR (400 MHz, DMSO-*d_6_*) δ 8.86 (1H, s), 8.64 (1H, s), 6.43 (1H, t, *J* = 6.4 Hz), 5.36 (1H, d, *J* = 4.4 Hz), 4.96 (1H, t, *J* = 5.6 Hz), 4.44 (1H, dt, *J* = 3.6, 9.6 Hz), 3.88 (1H, dd, *J* = 4.4, 8.0 Hz), 3.61 (1H, dt, *J* = 5.2, 12.0 Hz), 3.53 (1H, dt, *J* = 5.6, 12.0 Hz), 2.76 (1H, ddd, *J* = 6.4, 6.6, 14.0 Hz), 2.34 (1H, ddd, *J* = 3.6, 6.4, 14.0 Hz); ^13^C NMR (151 MHz, DMSO-*d_6_*) δ 151.8, 147.4, 144.8, 138.5, 122.8, 88.1, 84.2, 70.4, 61.4, 54.0; HRMS (ESI-TOF) calcd. for C_10_H_12_IN_4_O_3_^+^ [M+H]^+^: 362.9949, found: 362.9947.

**2'-Deoxy-5'-*O*-(4,4'-dimethoxytrityl)-6-iodo-*β*-D-purineriboside (1)**


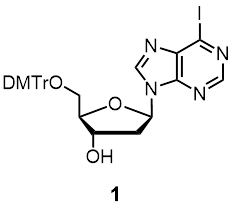
A suspension of compound **S3** (1.15 g, 3.18 mmol) in CH_2_Cl_2_ (130 ml) was added *N*,*N*-diisopropylethylamine (1.5 ml, 7.94 mmol) and 4,4’-dimethoxytrityl chloride (2.15 g, 6.35 mmol) at 0 °C, and the reaction mixture was stirred for 15 min at room temperature. The reaction was quenched by addition of MeOH (5 ml), and the resulting mixture was concentrated under reduced pressure. The residue was taken up in CH_2_Cl_2_, washed with saturated aqueous solution of NaHCO_3_, brine, dried over Na_2_SO_4_ and concentrated under reduced pressure. The residue was purified by silica gel column chromatography (CH_2_Cl_2_:CH_3_OH 100:1 to 30:1 with 0.01% triethylamine) to afford compound **1** (71%, 1.49 g, 2.26 mmol) as a pale-yellow foam.

^1^H NMR (400 MHz, CDCl_3_) δ 8.54 (1H, s), 8.28 (1H, s), 7.60 (2H, dd, *J* = 1.6, 8.2 Hz), 7.30-7.21 (7H, m), 6.80 (4H, d, *J* = 8.8 Hz), 6.46 (1H, t, *J* = 6.4 Hz), 4.70 (1H, dd, *J* = 3.6, 9.6 Hz), 4.18-4.15 (1H, m), 3.79 (6H, s), 3.40 (2H, ddd, *J* = 4.4, 10.4, 18.8 Hz), 2.88 (1H, ddd, *J* = 6.4, 6.4, 13.6 Hz), 2.58 (1H, ddd, *J* = 3.6, 6.4, 13.6 Hz); ^13^C NMR (151 MHz, CDCl_3_) δ 158.8, 152.0, 147.5, 144.5, 143.2, 139.4, 135.7, 135.6, 130.1, 128.2, 128.1, 127.2, 122.4, 113.4, 86.7, 86.5, 85.1, 72.8, 63.7, 55.4, 40.4; HRMS (ESI-TOF) calcd. for C_31_H_29_IN_4_NaO_5_^+^ [M+Na]^+^: 687.1075, found: 687.1077.

**5-Iodo-2-[2-(trimethylsilyl)ethoxy]pyridine (S4)**


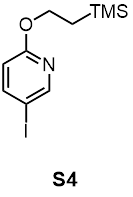
To a suspension of sodium hydride (60% in mineral oil, 750 mg, 18.8 mmol) in DMF (19 ml) and THF (188 ml) was added 2-(trimethylsilyl)ethanol (2.7 ml, 18.8 mmol) dropwise at 0 °C. After stirring for 15 min at the same temperature, a solution of 2-chloro-5-iodopyridine (3 g, 12.5 mmol) in THF (9 ml) was added dropwise, and the reaction mixture was refluxed for 24 h at 75 **°**C. The reaction was quenched with saturated aqueous solution of NH_4_Cl and extracted with AcOEt. The organic layer was separated, washed with brine, dried over Na_2_SO_4_ and evaporated under reduced pressure. The residue was purified by silica gel column chromatography (Hexane:AcOEt = 100:0 to 96:4) to afford compound **S4** (59%, 5.68 g, 20.7 mmol) as a colorless oil.

^1^H NMR (400 MHz, CDCl_3_) δ 8.31 (1H, dd, *J* = 0.8, 8.8 Hz), 7.75 (1H, dd, *J* = 2.4, 8.8 Hz), 6.54 (1H, dd, *J* = 0.8, 8.8 Hz), 4.36-4.32 (2H, m), 1.12-1.08 (2H, m), 0.07 (9H, s); ^13^C NMR (151 MHz, CDCl_3_) δ 163.5,152.9, 146.3, 113.8, 81.8, 64.3, 17.6, -1.2; HRMS (ESI-TOF) *No molecular ion peak was detected in the mass-spectra for this compound.

**5-(Trimethylsilyl)ethynyl-2-[2-(trimethylsilyl)ethoxy]pyridine (S5)**


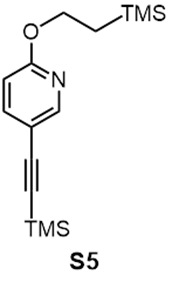
To a solution of compound **S4** (1 g, 3.11 mmol), bis(triphenylphosphine)-palladium (II) dichloride (109 mg, 0.155 mmol), copper (I) iodide (30 mg, 0.155 mmol) in THF (32 ml) was added trimethylsilylacetylene (0.9 ml, 4.67 mmol) and triethylamine (1.7 ml, 9.34 mmol), and the reaction mixture was stirred for 3 h at room temperature. The reaction mixture was concentrated under reduced pressure, and the residue was purified by silica gel column chromatography (Hexane:CH_2_Cl_2_ = 100:0 to 10:1) to afford compound **S5** (90%, 818 mg, 2.80 mmol) as a dark brown oil.

^1^H NMR (400 MHz, CDCl_3_) δ 8.26 (1H, dd, *J* = 0.8, 2.4 Hz), 7.59 (1H, dd, *J* = 2.4, 8.8 Hz), 6.62 (1H, dd, *J* = 0.8, 8.8 Hz), 4.41-4.37 (2H, m), 1.13-1.09 (2H, m), 0.25 (9H, s), 0.07 (9H, s); ^13^C NMR (151 MHz, CDCl_3_) δ 163.4, 150.8, 141.6, 112.8, 110.8, 102.3, 95.7, 64.3, 31.1, 17.7, 0.1, 0.1, -1.2; HRMS (ESI-TOF) *No molecular ion peak was detected in the mass-spectra for this compound.

**5-Ethynyl-2-[2-(trimethylsilyl)ethoxy]pyridine (2)**


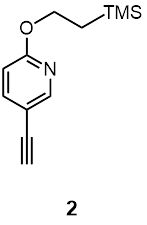
To a solution of compound **S5** (818 mg, 2.81 mmol) in CH_3_OH (28 ml) was added K_2_CO_3_ (777 mg, 5.62 mmol), and the reaction mixture was stirred for 2 h at room temperature. The solvent was removed under reduced pressure. The residue was diluted with CH_2_Cl_2_, filtered over celite, and the filtrate was evaporated under reduced pressure. The residue was purified by silica gel column chromatography (Hexane: CH_2_Cl_2_ = 100:0 to 20:1 to 5:1 to 1:1) to afford compound **2** (83%, 509 mg, 2.32 mmol) as a pale-yellow gel.

^1^H NMR (400 MHz, CDCl_3_) δ 8.29 (1H, dd, *J* = 0.8, 2.4 Hz), 7.62 (1H, dd, *J* = 2.4, 8.8 Hz), 6.65 (1H, dd, *J* = 0.8, 8.8 Hz), 4.42-4.32 (2H, m), 3.10 (1H, s), 1.14-1.10 (2H, m), 0.71 (9H, s); ^13^C NMR (151 MHz, CDCl_3_) δ 163.7, 150.0, 141.6, 111.7, 111.0, 81.0, 78.6, 64.4, 53.6 17.7, -1.2; HRMS (ESI-TOF) *No molecular ion peak was detected in the mass-spectra for this compound.

**2'-Deoxy-5'-*O*-(4,4'-dimethoxytrityl)-6-{[2-(2-trimethylsilyl)ethoxy]pyridin-5-yl}ethynyl-*β*-D-purineriboside (3)**


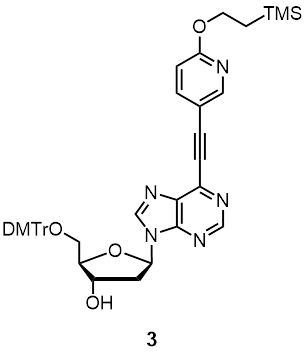
A solution of compound **1** (400 mg, 0.602 mmol), compound **2** (265 mg, 1.21 mmol), bis(triphenylphosphine)palladium (II) dichloride (21 mg, 0.03 mmol), copper (I) iodide (6 mg, 0.03 mmol) in CH_3_CN (20 ml) was added triethylamine (1 ml, 6.02 mmol), and the reaction mixture was stirred overnight at room temperature. The solution was concentrated under reduced pressure. The residue was diluted with AcOEt, filtered over celite and concentrated under reduced pressure. The residue was purified by silica gel column chromatography (Hexane:Acetone = 20:1 to 16:1 to 10:1 to 1:1 with 0.01% of triethylamine) to afford compound **3** (77%, 349 mg, 0.464 mmol) as a brown foam.

^1^H NMR (400 MHz, CDCl_3_) δ 8.86 (1H, s), 8.54 (1H, dd, *J* = 0.8, 2.4 Hz), 8.27 (1H, s), 7.86 (1H, dd, *J* = 2.4, 8.8 Hz), 7.40-7.36 (2H, m), 7.30-7.21 (7H, m), 6.81-6.79 (4H, m), 6.73 (1H, dd, *J* = 0.8, 8.8 Hz), 6.51 (1H, t, *J* = 6.4 Hz), 4.72 (1H, dt, *J* = 4.0, 9.6 Hz), 4.46-4.42 (2H, m), 4.17 (1H, dd, *J* = 4.8, 8.4 Hz), 3.79 (6H, s), 3.43 (2H, ddd, *J* = 4.8, 10.2, 17.4 Hz), 2.90 (1H, ddd, *J* = 6.4, 6.6, 14.0 Hz ), 2.59 (1H, ddd, *J* = 4.0, 6.4, 14.0 Hz), 1.99 (1H, d, *J* = 3.6 Hz), 1.16-1.12 (2H, m), 0.08 (9H, s); ^13^C NMR (151 MHz, CDCl_3_) δ 164.3, 158.5, 152.4, 151.8, 150.9, 144.4, 143.8, 141.9, 141.7, 135.6, 135.6. 135.2, 135.1, 135.0, 134.6, 129.4, 113.2, 111.1, 110.8, 86.6, 86.3, 85.7, 84.7, 72.5, 64.5, 63.6, 55.2, 53.7, 46.1, 40.2, 17.5, 1.4; HRMS (ESI-TOF) calcd. for C_43_H_46_N_5_O_6_Si^+^ [M+H]^+^: 756.3212, found: 756.3213.

**2'-Deoxy-3'-*O*-[2-cyanoethoxy(diisopropylamino)phosphino]-5'-*O*-(4,4'-dimethoxytrityl)-6-{[2-(2-trimethylsilyl)ethoxy]pyridin-5-yl}ethynyl-*β*-D-purineriboside (4)**


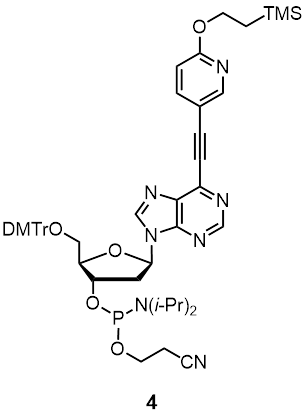
To a suspension of compound **3** (270 mg, 0.357 mmol) and *N*,*N*-diisopropylamine tetrazolide (61 mg, 0.357 mmol) in CH_3_CN (7.0 ml) was added 2-cyanoethyl *N*,*N*,*N*′,*N*′-tetraisopropylphosphorodiamidite (171 μl, 0.537 mmol), and the reaction mixture was stirred overnight at room temperature. The solution was diluted with AcOEt and washed with brine. The organic layer was dried over Na_2_SO_4_ and evaporated under reduced pressure. The residue was purified by silica gel column chromatography (Hexane:AcOEt = 3:1 to 2:1 to 1:1) to afford compound **4** (72%, 246 mg, 0.257 mmol) as a pale yellow foam.

^31^P NMR (CDCl_3_, 162 MHz) δ 148.9, 148.8; HRMS (ESI-TOF) calcd. for C_52_H_63_N_7_O_7_PSi^+^ [M+H]^+^: 956.4290, found: 956.4303.

**2-Amino-5-ethynylpyrimidine (5)**


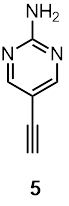
To a solution of 2-amino-5-iodopyrimidine (1.5 g, 6.79 mmol), bis(triphenylphosphine)palladium (II) dichloride (240 mg, 0.34 mmol), copper (I) iodide (65 mg, 0.34 mmol) in CH_3_CN (57 ml) was added trimethylsilylacetylene (2.5 ml, 13.6 mmol) and triethylamine (6 ml, 40.7 mmol), and the reaction mixture was stirred for 4 h at room temperature. The reaction mixture was concentrated under reduced pressure, and the residue was dissolved in CH_3_OH (75 ml). To the solution was added K_2_CO_3_ (9.4 g, 67.9 mmol) and the mixture was stirred for 2 h. The reaction mixture was filtered over celite and the filtrate was concentrated under reduced pressure. The residue was purified by silica gel column chromatography (CH_2_Cl_2_:AcOEt = 25:1 to 20:1 to 10:1 to 5:1) to afford compound **5** (59%, 476 mg, 4.01 mmol) as an orange solid.

^1^H NMR (400 MHz, CDCl_3_) δ 8.40 (2H, s), 5.20 (2H, brs), 3.19 (1H, s);^13^C NMR (151 MHz, CDCl_3_) δ 161.4, 108.1, 80.9, 78.3; HRMS (ESI-TOF) calcd. for C_5_H_6_N_3_^+^ [M+H]^+^: 120.0556, found: 120.0566.

**2'-Deoxy-5'-*O*-(4,4'-dimethoxytrityl)-6-(2-aminopyrimidin-5-yl)ethynyl-*β*-D-purineriboside (6)**


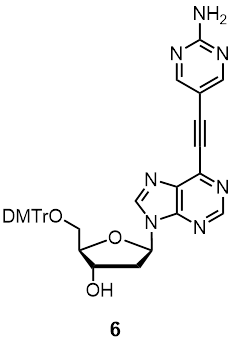
A solution of compound **1** (400 mg, 0.602 mmol), compound **5** (108 mg, 0.907 mmol), bis(triphenylphosphine)palladium (II) dichloride (127 mg, 0.181 mmol), copper (I) iodide (35 mg, 0.181 mmol) in DMF (20 ml) was added triethylamine (0.5 ml, 3.01 mmol), and the reaction mixture was stirred for 2.5 h at 40 °C. The solution was concentrated under reduced pressure. The residue was diluted with AcOEt and filtered over celite. The filtrate was washed with water, saturated aqueous solution of NH_4_Cl, brine, dried over Na_2_SO_4_ and evaporated under reduced pressure. The residue was purified by silica gel column chromatography (CH_2_Cl_2_:CH_3_OH = 50:1 to 40:1 to 30:1 to 15:1 with 0.01% of triethylamine) to afford compound **6** (72%, 284 mg, 0.433 mmol) as a yellow solid.

^1^H NMR (400 MHz, CDCl_3_) δ 8.86 (1H, s), 8.65 (2H, s), 8.28 (1H, s), 7.40-7.38 (2H, m), 7.31-7.19 (7H, m), 6.82-6.79 (4H, m), 6.51 (1H, t, *J* = 6.4 Hz), 5.36 (2H, brs), 4.73-4.72 (1H, m), 4.17 (1H, *J* = 4.8, 8.6 Hz), 3.79 (6H, s), 3.42 (2H, ddd, *J* = 4.8, 10.2, 17.2 Hz), 2.91 (1H, ddd, *J* = 6.4, 6.6, 13.6 Hz), 2.63 (1H, ddd, *J* = 4.4, 6.4, 13.6 Hz); ^13^C NMR (151 MHz, CDCl_3_) δ 161.8, 161.7, 158.6, 152.5, 151.0, 144.4, 143.8, 141.4, 135.6, 135.5, 134.5, 133.4, 130.9, 130.0, 128.8, 128.0, 127.9, 127.0, 113.2, 93.1, 88.0, 86.7, 86.2, 84.6, 72.7, 68.2, 63.6, 55.2, 40.1; HRMS (ESI-TOF) calcd. for C_37_H_34_N_7_O_5_^+^ [M+H]^+^: 656.2616, found: 656.2598.

**2'-Deoxy-5'-*O*-(4,4'-dimethoxytrityl)-6-(2-{[(dimethylamino)methylene]amino}pyrimidin-5-yl)ethynyl-*β*-D-purineriboside (7)**

To a solution of compound **6** (138 mg, 0.21 mmol) in CH_3_OH (2.1 ml) was added *N*,*N*-dimethylformamide dimethylacetal (165 μl, 1.05 mmol), and the reaction mixture was stirred for 2 h at 40°C. The solvent was removed under reduced pressure. The residue was purified by silica gel column chromatography (AcOEt:CH_3_OH = 100:0 to 20:0 to 10:1 to 5:1) to afford compound **7** (72%, 108 mg, 0.152 mmol) as a pale-yellow foam.

^1^H NMR (400 MHz, CDCl_3_) δ 8.86 (1H, s), 8.80 (2H, s), 8.76 (1H, s), 8.28 (1H, s), 7.39-7.37 (2H, m), 7.30-7.20 (7H, m), 6.79 (4H, d, *J* = 8.4 Hz), 6.51 (1H, t, *J* = 6.4 Hz), 4.74-4.71 (1H, m), 4.18 (1H, dd, *J* = 4.8, 8.4 Hz), 3.78 (6H, s), 3.42 (2H, ddd, *J =* 4.8, 10.2, 15.8 Hz), 3.21 (3H, s), 3.19 (3H. s), 2.90 (1H, ddd, *J* = 6.4, 6.6, 13.6 Hz), 2.60 (1H, ddd, *J* = 4.0, 6.4, 13.6 Hz); ^13^C NMR (151 MHz, CDCl_3_) δ 165.8, 161.5, 159.1, 158.7, 152.7, 151.2, 144.5, 144.0, 141.5, 135.72, 135.66 134.8, 130.12, 130.11, 128.2. 128.1, 127.2, 113.4, 110.3, 93.5, 89.2, 86.8, 86.4, 84.8, 72.8, 63.8, 55.4, 53.6, 41.5, 40.3, 35.5; HRMS (ESI-TOF) calcd. for C_40_H_39_N_8_O_5_^+^ [M+H]^+^: 711.3038, found: 711.3054.

**2'-Deoxy-3'-*O*-[2-cyanoethoxy(diisopropylamino)phosphino]-5'-*O*-(4,4'-dimethoxytrityl)-6-(2-{[(dimethylamino)methylene]amino}pyrimidin-5-yl)ethynyl-*β*-D-purineriboside (7)**

To a suspension of compound **7** (153 mg, 0.215 mmol) and *N*,*N*-diisopropylamine tetrazolide (38 mg, 0.215 mmol) in CH_3_CN (4.5 ml) was added 2-cyanoethyl *N*,*N*,*N*′,*N*′-tetraisopropylphosphorodiamidite (105 μl, 0.323 mmol), and the reaction mixture was stirred overnight at room temperature. The solution was diluted with AcOEt and washed with brine. The organic layer was dried over Na_2_SO_4_ and evaporated under reduced pressure. The residue was purified by silica gel column chromatography (AcOEt to AcOEt:MeOH = 20:1 to 15:1 to 5:1) to afford compound **8** (68%, 137 mg, 0.146 mmol) as a pale yellow foam.

^31^P NMR (CDCl_3_, 162 MHz) δ 148.9, 148.8; HRMS (ESI-TOF) calcd. for C_49_H_56_N_10_O_6_P^+^ [M+H]^+^: 911.4116, found: 911.4117.

**Scheme S2**. Full synthesis scheme for ^O^Pz and ^N^Pz phosphoramidites.

**3-Chloro-6-[2-(trimethylsilyl)ethoxy]pyridazine (S6)**


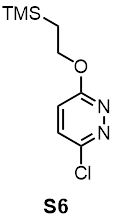
To a suspension of sodium hydride (60% in mineral oil, 1.2 g. 29.5 mmol) in a mixture of DMF (2 ml) and THF (45 ml) was added 2-(trimethylsilyl)ethanol (4.0 ml, 28.2 mmol) at 0 °C. After stirring for 30 min at the same temperature, a solution of 3,6-dichloropyridazine (4.0 g, 26.9 mmol) in THF (45 ml) was added dropwise, and the reaction mixture was stirred overnight at room temperature. The reaction was quenched with saturated aqueous solution of NH_4_Cl and extracted with AcOEt. The organic layer was separated, washed with brine, dried over Na_2_SO_4_ and evaporated under reduced pressure. The residue was purified by silica gel column chromatography (Hexane:AcOEt = 100:0 to 15:1) to afford compound **S6** (80%, 5.0 g, 21.6 mmol) as a white amorphous.

^1^H NMR (400 MHz, CDCl_3_) δ 7.34 (1H, d, *J* = 9.2 Hz), 6.90 (1H, d, *J* = 9.2 Hz), 4.60-4.56 (2H, m), 1.19-1.15 (2H, m), 0.08 (9H, s); ^13^C NMR (151 MHz, CDCl_3_) δ 164.5, 150.8, 130.8, 120.3, 66.3, 17.3, -1.3; HRMS (ESI-TOF) calcd. for C_9_H_16_ClN_2_OSi^+^ [M+H]^+^: 231.0715, found: 231.0707.

**3-Chloro-5-iodo-6-[2-(trimethylsilyl)ethoxy]pyridazine (9)**


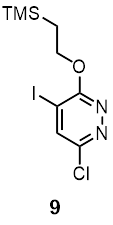
To a solution of 2,2,6,6-tetramethylpiperidine (16.2 ml, 95.0 mmol) in THF (450 ml) was added *n*-butyllithium (2.5 M in hexane, 37.8 ml, 95.0 mmol) at -78 °C, and the reaction mixture was stirred for 30 min at 0 °C. The mixture was cooled to -78 °C and a solution of compound **S6** (10.5 g, 45.5 mmol) in THF (90 ml) was added dropwise. After stirring for 1 h, a solution of iodine (12.0 g, 47.5 mmol) in THF (60 ml) was added dropwise, and the reaction mixture was stirred for another 30 min at -78 °C. The reaction was quenched with saturated aqueous solution of Na_2_S_2_O_3_ and extracted with AcOEt. The organic layer was washed with brine, dried over Na_2_SO_4_ and evaporated under reduced pressure. The residue was purified by silica gel column chromatography (Hexane:AcOEt = 100:0 to 97:3) to afford compound **9** (63%, 10.2 g, 28.6 mmol) as a pale-yellow powder.

^1^H NMR (400 MHz, CDCl_3_) δ 7.89 (1H, s), 4.65-4.60 (2H, m), 1.27-1.23 (2H, m), 0.1 (9H, s); ^13^C NMR (151 MHz, CDCl_3_) δ 163.2, 149.2, 139.9, 94.2, 17.5, 0.1, -1.2; HRMS (ESI-TOF) calcd. for C_9_H_15_ClIN_2_OSi^+^ [M+H]^+^: 357.5709, found: 357.5708.

**2'-Deoxy-3',5'-di-*O*-(*tert*-butyldimethylsilyl)thymidine (S7)**


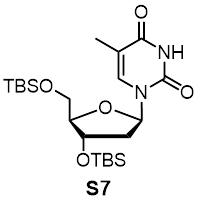
To a solution of thymidine (15.0 g, 61.9 mmol) in DMF (63 ml) was added imidazole (16.9 g, 248.2 mmol) and TBSCl (20.5 g, 136.2 mmol), and the reaction mixture was stirred overnight at room temperature. The reaction mixture was concentrated under reduced pressure, and the residue was taken up in AcOEt. The organic layer was washed with H_2_O, saturated aqueous solution of NH_4_Cl followed by brine, dried over Na_2_SO_4_ and evaporated under reduced pressure to afford compound **S7** (99%, 28.7 g, 61.0 mmol) as a white powder.

^1^H NMR (400 MHz, CDCl_3_) δ 8.07 (1H, s), 7.48 (1H, d, *J* = 1.2 Hz), 6.33 (1H, dd , *J* = 6.0, 8.2 Hz), 4.40 (1H, dt, *J* = 2.4, 6.0 Hz), 3.94 (1H, dd, *J* = 2.4, 5.0 Hz), 3.87 (1H, dd, *J* = 2.4, 11.4 Hz), 3.76 (1H, dd, *J* = 2.4, 11.4 Hz), 2.24 (1H, ddd, *J* = 2.4, 6.0, 13.0 Hz), 2.05-1.97 (1H, m), 1.92 (3H, d, *J* = 1.2 Hz), 0.93 (9H, s), 0.90 (9H, s), 0.11 (6H, d, *J* = 1.2 Hz), 0.08 (6H, d, *J =* 2.8 Hz); ^13^C NMR (151 MHz, CDCl_3_) δ 161.1, 150.5, 135.6, 111.0, 87.9, 84.9, 72.3, 63.1, 41.5, 26.0, 25.9, 18.5, 18.1, 12.7, -4.5, -4.7, -5.3, -5.3; HRMS (ESI-TOF) calcd. for C_22_H_43_N_2_O_5_Si_2_^+^ [M+H]^+^: 471.2705, found: 471.2715.

**3,5-Di-*O*-(*tert*-butyldimethylsilyl)-1,2-didehydro-1,2-dideoxy-D-ribofuranose (S8)**


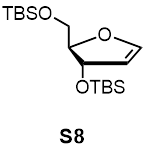
A mixture of compound **S7** (8.0 g, 17.0 mmol) and ammonium sulfate (0.9 g, 6.78 mmol) was suspended in 1,1,1,3,3,3-hexamethyldisilazane (41.6 ml, 192 mmol), and the resulting mixture was refluxed 145 °C for 3 h. The reaction mixture was concentrated under reduced pressure, and the residue was taken up in hexane and stirred for 15 min at room temperature. The white precipitate was filtered over celite, and the filtrate was washed with H_2_O followed by brine, dried over Na_2_SO_4_ and evaporated under reduced pressure. The residue was purified by silica gel column chromatography (Hexane:Ether = 100:0 to 15:1) to afford compound **S8** (63 %, 3.67 g, 10.7 mmol) as a pale yellow oil.

^1^H NMR (400 MHz, CDCl_3_) δ 6.47 (1H, dd , *J* = 1.2, 2.8 Hz), 5.01 (1H, t, *J* = 2.8 Hz), 4.86 (1H, dt, *J* = 0.8, 2.8 Hz), 4.29 (1H, dt, *J* = 2.8, 6.4 Hz), 3.69 (1H, dd, *J* = 5.6, 10.4 Hz), 3.51 (1H, dd, *J* = 6.4, 10.4 Hz), 0.89 (9H, s), 0.89 (9H, s), 0.86 (6H, d, *J* = 0.8 Hz), 0.06 (6H, d, *J* = 3.6 Hz); ^13^C NMR (151 MHz, CDCl_3_) δ 149.1, 103.6, 89.1, 76.1, 63.0, 26.1, 26.0, 18.5, 18.2 -4.1, -4.3, -5.2, -5.2; HRMS (ESI-TOF) calcd. for C_17_H_37_O_3_Si_2_^+^ [M+H]^+^: 367.2101, found: 367.2077.

**3-*O*-(*tert*-Butyldimethylsilyl)-1,2-didehydro-1,2-dideoxy-D-ribofuranose (10)**


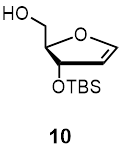
A solution of compound **S8** (1.0 g, 2.90 mmol) in THF (14.5 ml) was added a solution of tetrabutylammonium fluoride (1.0 M in THF, 2.6 ml, 2.60 mmol) at 0 °C, and the reaction mixture was stirred for 40 min at the same temperature. The reaction was quenched by the addition of silica gel, and the solvent was removed under reduced pressure. The residue was purified by silica gel column chromatography (Hexane:Ether = 30:1 to 20:0 to 1:1) to afford compound **10** (88%, 589 mg, 2.56 mmol) as a pale-yellow oil.

^1^H NMR (400 MHz, CDCl_3_) δ 6.40 (1H, *J* = 1.2 Hz), 4.97 (1H, t, *J* = 2.4 Hz), 4.73-4.71 (1H, m), 4.25 (1H, ddd, *J* = 3.2, 3.6, 6.8 Hz), 3.63-3.57 (1H, m), 3.53-3.47 (1H, m), 2.22 (1H, t, *J* = 6.0 Hz), 0.80 (9H, s). 0.00 (6H, s); ^13^C NMR (151 MHz, CDCl_3_) δ 148.8, 104.3, 89.4, 63.1, 15.9, 18.2, -4.2, -4.4; HRMS (ESI-TOF) *No molecular ion peak was detected in the mass-spectra for this compound.

**3-Chloro-5-(2'**-**deoxy**-***β***-**D**-**ribofuranos**-**1**-**yl)-6-[2-(trimethylsilyl)ethoxy]pyridazine (11)**


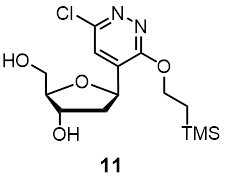
To a solution of **9** (1.6 g, 4.49 mmol) and compound **10** (1.0 g, 4.49 mmol) in CHCl_3_ (45ml) was added a solution of palladium (II) acetate (201 mg, 0.90 mmol) and tris(pentafluorophenyl)phosphine (955 mg, 1.79 mmol) in CHCl_3_ (45 ml) followed by silver carbonate (1.9 g, 6.73 mmol). The reaction mixture was refluxed at 70 °C for 6 h. The reaction mixture was filtered over celite, and the filtrate was concentrated under reduced pressure. The residue was dissolved in THF (45 ml) and triethylamine trihydrofluoride (877 μl, 5.38 mmol) was added at room temperature. After stirring for 1 h, silica gel was added and the volatiles were evaporated under reduced pressure. The residue was purified by silica gel column chromatography (CH_2_Cl_2_:CH_3_OH = 100:0 to 94:6), and the appropriate fractions were combined and evaporated. The residue was subsequently dissolved in a mixture of AcOH (22 ml) and CH_3_CN (22 ml), and sodium triacetoxyborohydride (1.5 g, 6.73 mmol) was added at 0 °C. After stirring for 1 h at the same temperature, the reaction was quenched with acetone and concentrated under reduced pressure. The residue was purified by silica gel column chromatography (CH_2_Cl_2_:CH_3_OH = 100:0 to 95:5) to afford compound **11** (66%, 997 mg, 2.87 mmol) as a yellow foam.

^1^H NMR (400 MHz, CD_3_OD) δ 7.30 (1H, d, *J* = 1.2 Hz), 5.08 (1H, dd, *J* = 6.0, 9.6 Hz), 4.51-4.46 (2H, m), 4.20 (1H, ddd, *J* = 2.0, 2.4, 6.0 Hz), 3.87 (1H, ddd, *J* = 2.4, 2.8, 6.4 Hz), 3.59 (1H, dd, *J* = 4.4, 11.6 Hz), 3.53 (1H, dd, *J* = 5.2, 11.6 Hz), 2.36 (1H, ddd, *J* = 2.0, 6.0, 13.0 Hz), 1.68 (1H, ddd, *J* = 6.0, 8.4, 13.0 Hz), 1.15-1.10 (2H, m), 0.00 (9H, s); ^13^C NMR (151 MHz, CD_3_OD) δ 163.1, 152.4, 138.7, 127.4, 89.2, 74.9, 73.8, 67.4, 63.6, 42.4, 18.4, -1.4; HRMS (ESI-TOF) calcd. for C_14_H_24_ClN_2_O_4_Si^+^ [M+H]^+^: 347.1188, found: 347.1192.

**3-Chloro-5-[5'-*O*-(4,4'-dimethoxytrityl)-2'**-**deoxy**-***β***-**D**-**ribofuranos**-**1**-**yl]-6-[2-(trimethylsilyl)ethoxy] pyridazine (12)**


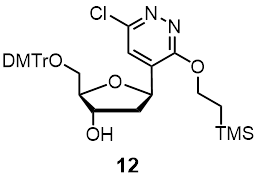
To a solution of compound **11** (694 mg, 2.00 mmol) in pyridine (20 ml) was added 4,4’-dimethoxytrityl chloride (881 mg, 2.60 mmol) at 0 °C, and the mixture was stirred for 1 h at room temperature. The reaction was quenched with MeOH and concentrated under reduced pressure. The residue was purified by silica gel column chromatography (CH_2_Cl_2_:CH_3_OH = 100:0 to 99:1 to 98:2) to afford compound **12** (99%, 1.29 g, 1.97 mmol) as a white foam.

^1^H NMR (400 MHz, CDCl_3_) δ 7.70 (1H, d, *J* = 1.2 Hz), 7.44-7.41 (2H, m), 7.34-7.20 (7H, m), 6.84 (4H, dd, *J* = 0.8, 9.2 Hz ), 5.18 (1H, dd, *J* = 6.4, 9.4 Hz), 4.66-4.54 (2H, m), 4.38 (1H, dd, *J* = 3.2, 6.0 Hz), 4.10 (1H, dd, *J* = 4.8, 7.6 Hz), 3.79 (6H. s), 3.33 (1H, dd, *J =* 4.8, 10.0 Hz), 3.24 (1H, dd, *J* = 4.8, 10.0 Hz), 2.51 (1H, ddd, *J* = 2.4, 6.4, 13.2 Hz), 1.90-1.85 (2H, m), 1.20-1.16 (2H, m), 1.80 (1H, d, *J* = 3.6 Hz), 0.08 (9H, s); ^13^C NMR (151 MHz, CDCl_3_) δ 161.6, 158.7, 151.4, 144.8, 136.0, 135.9, 135.9, 130.2, 130.1, 128.2, 128.0, 127.1, 125.9, 113.4, 86.6, 86.3, 74.2, 73.8, 66.4, 64.3, 55.4, 41.3, 17.9, 0.1, -1.3; HRMS (ESI-TOF) calcd. for C_35_H_42_ClN_2_O_6_Si^+^ [M+H]^+^: 649.2495, found: 649.2513.

**5-[(Triisopropylsilyl)ethynyl]-3-[5'-*O*-(4,4'-dimethoxytrityl)-2'**-**deoxy**-***β***-**D**-**ribofuranos**-**1**-**yl]-2-[2-(trimethylsilyl)ethoxy]pyridazine (13)**


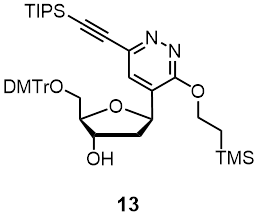
A suspension of compound **12** (450 mg, 0.69 mmol), (triisopropylsilyl)acetylene (624 μl, 2.77 mmol), 2-dicyclohexylphosphino-2′,4′,6′-triisopropylbiphenyl (XPhos; 69 mg, 0.145 mmol), XPhos precatalyst^2^ (69 mg, 0.088 mmol) and Cs_2_CO_3_ (677 mg, 2.08 mmol) in CH_3_CN was heated for 4.5 h at 85 °C in a sealed tube. The reaction mixture was concentrated under reduced pressure, and the residue was purified by silica gel column chromatography (CH_2_Cl_2_:AcOEt = 100:0 to 90:10) to afford compound **13** (78%, 428 mg, 0.54 mmol) as a white foam.

^1^H NMR (400 MHz, CDCl_3_) δ 7.63 (1H, d, *J* = 1.2 Hz), 7.45-7.43 (2H, m), 7.33-7.18 (7H, m), 6.83 (4H, dd, *J* = 1.6, 8.8 Hz ), 5.18 (1H, dd, *J* = 6.0, 9.6 Hz), 4.71-4.59 (2H, m), 4.35-4.34 (1H, m), 4.10 (1H, dt, *J* = 2.8, 7.2 Hz), 3.78 (6H, s), 3.33 (1H, dd, *J* = 4.8, 9.8 Hz), 3.25 (1H, dd, *J* = 5.6, 9.8 Hz), 2.60 (1H, ddd, *J* = 2.4, 6.0, 8.6 Hz), 1.86-1.78 (2H, m), 1.22-1.09 (23H, m), 0.08 (9H, s); ^13^C NMR (151 MHz, CDCl_3_) δ 160.7, 158.7, 144.7, 143.6, 136.1, 136.0, 131.7, 130.1, 128.2, 128.1, 127.5, 127.0, 113.3, 103.4, 94.2, 86.5, 86.2, 74.6, 73.8, 66.2, 64.2, 55.3, 41.4, 29.4, 18.7, 17.9, 11.3, 0.1, -1.3; HRMS (ESI-TOF) calcd. for C_46_H_63_N_2_O_6_Si_2_^+^ [M+H]^+^: 795.4219, found: 795.4214.

**5-Ethynyl-3-[5'-*O*-(4,4'-dimethoxytrityl)-2'**-**deoxy**-***β***-**D**-**ribofuranos**-**1**-**yl]-2-[2-(trimethylsilyl)ethoxy]-pyridazine (14)**


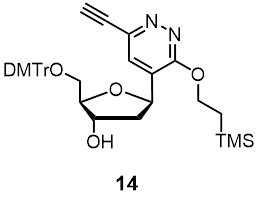
To a solution of compound **13** (397 mg, 0.50 mmol) in CH_3_CN (5 ml) was added silver (I) fluoride (666 mg, 5.25 mmol) portionwise, and the suspension was stirred overnight at room temperature in dark. The reaction was quenched by adding a saturated aqueous solution of NH_4_Cl, and the mixture was extracted with AcOEt. The organic layer was washed with brine, dried over Na_2_SO_4_ and evaporated under reduced pressure. The residue was purified by silica gel column chromatography (Hexane:AcOEt = 80:20 to 30:70) to afford compound **14** (78%, 250 mg, 0.39 mmol) as a pale yellow foam.

^1^H NMR (400 MHz, CDCl_3_) δ 7.70 (1H, d, *J* = 1.2 Hz), 7.44-7.41 (2H, m), 7.34-7.20 (7H, m), 6.85-6.82 (4H, m), 5.19 (1H, dd, *J* = 6.4, 9.6 Hz), 4.71-4.60 (2H, m), 4.36 (1H, dt, *J* = 3.2, 9.6 Hz), 4.13-4.08 (1H, m) 3.79 (6H, s), 3.33 (1H, dd, *J* = 4.8, 10.0 Hz), 3.24 (1H, dd, *J* = 4.8, 10.0 Hz), 2.50 (1H, ddd, *J* = 2.4, 6.4, 13.2 Hz), 2.05 (1H, s), 1.88-1.81 (2H, m), 1.28-1.16 (2H, m), 0.08 (9H, s); ^13^C NMR (151 MHz, CDCl_3_) δ 161.1, 158.8, 158.7, 147.5, 144.8, 142.7, 142.6, 139.6, 136.0, 135.9, 132.3, 131.7, 130.2, 130.1, 129.3, 128.2, 128.0, 128.0, 127.9, 127.9, 127.7, 127.8, 127.2, 127.0, 113.3, 113.3, 87.3, 86.5, 86.2, 81.6, 80.5, 80.4, 79.9, 79.8, 74.2, 73.9, 73.6, 73.5, 66.5, 66.4, 64.4, 63.4, 55.4, 41.4,17.9, -1.3; HRMS (ESI-TOF) calcd. for C_37_H_43_N_2_O_6_Si^+^[M+H]^+^: 639.2885, found: 639.2894.

**2-Bromo-6-[2-(trimethylsilyl)ethoxy]pyridine (S9)**


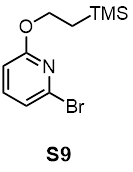
To a suspension of sodium hydride (60% in mineral oil, 1.5 g, 36.8 mmol) in a mixture of DMF (3 ml) and THF (70 ml) was added 2-(trimethylsilyl)ethanol (5.0 ml, 35.0 mmol) dropwise at 0 °C. After stirring for 15 min at the same temperature, a solution of 2,6-dibromopyridine (8.3 g, 35.0 mmol) in THF (70 ml) was added dropwise, and the reaction mixture was stirred overnight at 60 °C. The reaction was quenched with saturated aqueous solution of NH_4_Cl and extracted with AcOEt. The organic layer was separated, washed with brine, dried over Na_2_SO_4_ and evaporated under reduced pressure. The residue was purified by silica gel column chromatography (Hexane:AcOEt = 100:0 to 96:4) to afford compound **S9** (59%, 5.68 g, 20.7 mmol) as a colorless oil.

^1^H NMR (400 MHz, CDCl_3_) δ 7.39 (1H, dd, *J* = 0.4, 7.5 Hz), 7.02 (1H, dd, *J* = 0.4, 7.2 Hz), 6.63 (1H, dd, *J* = 0.4, 7.2 Hz), 4.40-4.36 (2H, m), 1.12-1.08 (2H, m), 0.07 (9H, s); ^13^C NMR (151 MHz, CDCl_3_) δ 163.7, 140.4, 138.8, 120.0, 109.7, 64.8, 17.6, −1.1; HRMS (ESI-TOF) calcd. for C_10_H_17_BrNOSi^+^ [M+H]^+^: 274.025, *peak not found.

**2-Iodo-****6-[2-(trimethylsilyl)ethoxy]pyridine (15)**


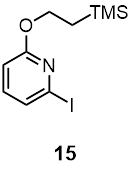
To a solution of compound **S9** (500 mg, 1.82 mmol) in THF (2.6 ml) was added *n*-butyllithium (2.5 M in hexane, 0.73 ml, 1.82 mmol) at -78 °C. After stirring for 1 h at the same temperature, a solution of iodine (509 mg, 2.01 mmol) in THF (2.6 ml) was added dropwise. The reaction mixture was warmed up to room temperature and was stirred for another 2 h. The reaction was quenched with saturated aqueous solution of Na_2_S_2_O_3_ and extracted with AcOEt. The organic layer was washed with brine, dried over Na_2_SO_4_ and evaporated under reduced pressure. The residue was purified by silica gel column chromatography (Hexane:AcOEt = 100:0 to 96:4) to afford compound **15** (84%, 490 mg, 1.53 mmol) as a colorless oil.

^1^H NMR (400 MHz, CDCl_3_) δ 7.27 (1H, d, *J* = 0.8 Hz), 7.15 (1H, dd, *J* = 7.4, 8.0 Hz), 6.63 (1H, dd, *J* = 0.8, 8.0 Hz), 4.39-4.34 (2H, m), 1.11-1.07 (2H, m), 0.07 (9H, s); ^13^C NMR (151 MHz, CDCl_3_) δ 163.4, 139.7, 127.4, 114.0, 110.2, 64.8, 17.6, -1.1; HRMS (ESI-TOF) calcd. for C_10_H_17_INOSi^+^ [M+H]^+^: 322.0119, found: 322.0113.

**5-[2-(Trimethylsilyl)ethoxy]pyridin-6-yl}ethynyl-3-[5'-*O*-(4,4'-dimethoxytrityl)-2'**-**deoxy**-***β***-**D**-**ribofuranos**-**1**-**yl]-2-[2-(trimethylsilyl)ethoxy]pyridazine (16)**


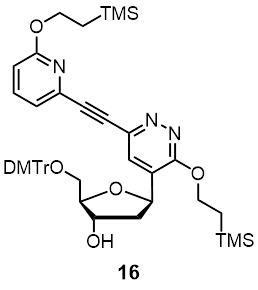
A solution of compound **14** (230 mg, 0.36 mmol), compound **15** (173 mg, 0.54 mmol), bis(triphenylphosphine)palladium (II) dichloride (25 mg, 0.036 mmol), CuI (7 mg, 0.036 mmol), triethylamine (200 μl, 1.44 mmol) in DMF (3.6 ml) was stirred overnight at room temperature. The reaction mixture was concentrated under reduced pressure, and the residue was purified by silica gel column chromatography (Hexane:AcOEt = 90:10 to 50:50) to afford compound **16** (83%, 250 mg, 0.30 mmol) as a pale yellow foam.

^1^H NMR (400 MHz, CDCl_3_) δ 7.77 (1H, d, *J* = 0.8 Hz), 7.49 (1H, dd, *J* = 7.2, 8.4 Hz), 7.45-7.43 (2H, m), 7.35-7.16 (7H, m), 7.07 (1H, dd, *J* = 0.8, 7.2 Hz) 6.85-6.81 (4H, m), 6.69 (1H, dd, *J* = 0.8, 8.4 Hz) 5.21 (1H, dd, *J* = 6.4, 9.2 Hz), 4.74-4.62 (2H, m), 4.42-4.36 (3H, m), 4.12-4.09 (1H, m) 3.73 (6H, s), 3.36 (1H, dd, *J* = 4.8, 10.0 Hz), 3.24 (1H, dd, *J* = 5.2, 10.0 Hz), 2.51 (1H, ddd, *J* = 2.4, 6.4, 13.2 Hz), 1.88-1.70 (2H, m), 1.27-1.16 (2H, m), 1.12-1.08 (2H, m), 0.09 (9H, s), 0.05 (9H, s); ^13^C NMR (151 MHz, CDCl_3_) δ 163.9, 160.9, 158.7, 144.7, 143.3, 139.5, 138.5, 136.0, 136.0, 132.0, 130.2, 130.1, 128.3, 127.9, 127.1, 121.3, 113.4, 112.2, 90.9, 86.6, 86.2, 84.9, 74.4, 73.7, 66.3, 64.4, 64.2, 55.3, 41.3. 18.0, 17.7, 0.1, -1.2, -1.2; HRMS (ESI-TOF) calcd. for C_47_H_58_N_3_O_7_Si_2_^+^ [M+H]^+^: 832.3808, found: 832.3808**.**

**5-[2-(Trimethylsilyl)ethoxy]pyridin-6-yl}ethynyl-3-{3'-*O*-[2-cyanoethoxy(diisopropylamino)phosphino]-5'-*O*-(4,4'-dimethoxytrityl)-2'**-**deoxy**-***β***-**D**-**ribofuranos**-**1**-**yl}-2-[2-(trimethylsilyl)ethoxy]pyridazine (17)**


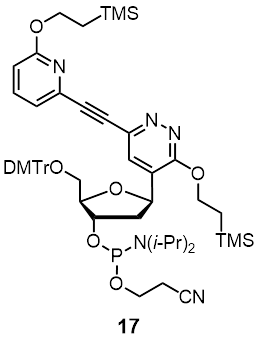
To a suspension of compound **16** (244 mg, 0.30 mmol) and *N*,*N*-diisopropylamine tetrazolide (51 mg, 0.30 mmol) in CH_3_CN (3.8 ml) was added 2-cyanoethyl *N*,*N*,*N*′,*N*′-tetraisopropylphosphorodiamidite (155 μl, 0.39 mmol), and the reaction mixture was stirred overnight at room temperature. The solution was diluted with AcOEt and washed with brine. The organic layer was dried over Na_2_SO_4_ and evaporated under reduced pressure. The residue was purified by silica gel column chromatography (Hexane:AcOEt = 90:10 to 70:30) to afford compound **17** (63%, 191 mg, 0.19 mmol) as a pale yellow foam.

^31^P NMR (CDCl_3_, 162 MHz) δ 148.6, 148.0; HRMS (ESI-TOF) calcd. for C_56_H_75_N_5_O_8_PSi_2_^+^ [M+H]^+^: 1032.4886, found: 1032.4894.

**5-(2-Aminopyrimidin-6-yl)ethynyl-3-[5'-*O*-(4,4'-dimethoxytrityl)-2'**-**deoxy**-***β***-**D**-**ribofuranos**-**1**-**yl]-2-[2-(trimethylsilyl)ethoxy]pyridazine (19)**


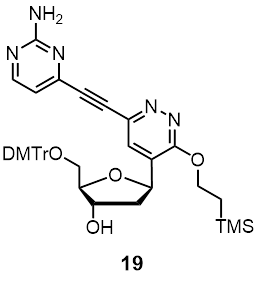
A solution of compound **14** (250 mg, 0.39 mmol), compound **18**^3^ (173 mg, 0.783 mmol), bis(triphenylphosphine)palladium (II) dichloride (27 mg, 0.039 mmol), copper (I) iodide (7.4 mg, 0.039 mmol), triethylamine (217 μl, 1.56 mmol) in DMF (3.9 ml) was stirred overnight at room temperature. The reaction mixture was concentrated under reduced pressure, and the residue was purified by silica gel column chromatography (CH_2_Cl_2_:CH_3_OH = 100:0 to 94:6) to afford compound **19** (84%, 240 mg, 0.33 mmol) as a pale-yellow foam.

^1^H NMR (400 MHz, CDCl_3_) δ 8.28 (1H, d, *J* = 4.8 Hz), 7.81 (1H, d, *J* = 1.2 Hz), 7.43-7.41 (2H, m), 7.35-7.26 (6H, m), 7.21-7.17 (1H, m) 6.87-6.83 (4H, m), 6.75 (1H, d, *J* = 4.8 Hz), 5.23-5.19 (3H, m), 4.75-4.63 (2H, m), 4.39-4.38 (1H, m), 4.10 (1H, dd, *J* = 2.8, 6.4 Hz), 3.76 (6H, s), 3.35 (1H, dd, *J* = 4.8, 10.0 Hz), 3.23 (1H, dd, *J* = 5.2, 10.0 Hz), 2.51 (1H, ddd, *J* = 2.4, 6.4, 13.2 Hz), 2.06 (1H. d. *J* = 4.0 Hz), 1.84 (1H, ddd, *J* = 6.4, 8.6, 13.2 Hz), 1.24-1.16 (2H, m), 0.09 (9H, s); ^13^C NMR (151 MHz, CDCl_3_) δ 163.0, 161.2, 158.9, 158.7, 158.7, 150.8, 144.8, 142.5, 136.0, 135.9, 132.2, 130.2, 130.1, 128.2, 128.1, 127.1, 114.5, 113.4, 88.5, 88.3, 86.6, 86.2, 74.4, 73.6, 66.6, 64.4, 55.4, 41.2, 18.0, 0.1, -1.2; HRMS (ESI-TOF) calcd. for C_41_H_46_N_5_O_6_Si^+^ [M+H]^+^: 732.3212, found: 732.3204.

**5-[(*N*-Phenoxyacetyl)-2-aminopyrimidin-6-yl]ethynyl-3-[5'-*O*-(4,4'-dimethoxytrityl)-2'**-**deoxy**-***β***-**D**-**ribofuranos**-**1**-**yl]-2-[2-(trimethylsilyl)ethoxy]pyridazine (20)**


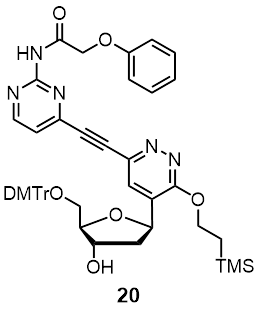
To a solution of compound **19** (175 mg, 0.24 mmol) in pyridine (4.8 ml) was added trimethylchlorosilane (211 μl, 1.67 mmol), and the reaction mixture was stirred for 40 min at room temperature. Subsequently, phenoxyacetyl chloride (49 μl, 0.36 mmol) was added, and the reaction mixture was stirred for another 2 h. The reaction was quenched with a saturated aqueous solution of NaHCO_3_ and extracted with AcOEt. The combined organic layer was washed with a saturated aqueous solution of NH_4_Cl followed by brine, dried over Na_2_SO_4_ and evaporated under reduced pressure. The residue was dissolved in THF (4.8 ml), and triethylamine (133 μl, 0.96 mmol) and triethylamine trihydrofluoride (154 μl, 0.96 mmol) were added. The solution was stirred at room temperature for 2 h, and the reaction was quenched with methoxytrimethylsilane (70 μl). The reaction mixture was concentrated under reduced pressure, and the residue was purified by silica gel column chromatography (CH_2_Cl_2_:AcOEt = 100:0 to 60:40) to afford compound **20** (58%, 120 mg, 0.14 mmol) as a yellow foam.

^1^H NMR (400 MHz, CDCl_3_) δ 9.01 (1H, brs), 8.69 (1H, d, *J* = 5.2 Hz), 7.88 (1H, d, *J* = 1.2 Hz), 7.44-7.41 (2H, m), 7.35-7.32 (8H, m), 7.31-7.20 (1H, m), 7.19 (1H, d, *J* = 5.2 Hz), 7.16-7.07 (1H, m), 7.05-7.00 (2H, m), 6.85-6.82 (4H, m), 5.22 (1H, dd, *J* = 6.4, 9.2 Hz), 4.76-4.64 (4H, m), 4.41 (1H, dd, *J* = 2.8, 6.8 Hz), 3.74 (6H, d, *J* = 1.2 Hz), 3.35 (1H, dd, *J* = 4.8, 10.0 Hz), 3.25 (1H, dd, *J* = 5.2, 10.0 Hz), 2.52 (1H, ddd, *J* = 2.4, 6.4, 13.2 Hz), 1.91-1.84 (2H, m), 1.30-1.17 (2H, m), 0.11 (9H, s); ^13^C NMR (151 MHz, CDCl_3_) δ 161.3, 159.1, 158.7, 158.7, 157.1, 157.0, 151.3, 144.8, 142.1, 136.0, 135.9, 132.3, 130.2, 130.1, 130.0, 129.9, 128.3, 128.2, 128.1, 127.1, 122.6, 119.9, 115.0, 113.4, 90.5, 87.8, 86.6, 86.2, 74.3, 73.6, 66.7, 64.3, 55.4, 41.3, 29.8, 18.0, 0.1, -1.3; HRMS (ESI-TOF) calcd. for C_49_H_52_N_5_O_8_Si^+^ [M+H]^+^: 866.3580, found: 866.3571.

**5-[*N*-(Phenoxyacetyl)-2-aminopyrimidin-6-yl]ethynyl-3-{3'-*O*-[2-cyanoethoxy(diisopropylamino)phosphino]-5'-*O*-(4,4'-dimethoxytrityl)-2'**-**deoxy**-***β***-**D**-**ribofuranos**-**1**-**yl}-2-[2-(trimethylsilyl)ethoxy]pyridazine (21)**


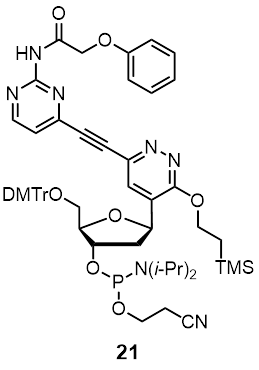
To a suspension of compound **20** (140 mg, 0.162 mmol) and *N*,*N*-diisopropylamine tetrazolide (28 mg, 0.162 mmol) in CH_3_CN (3.2 ml) was added 2-cyanoethyl *N*,*N*,*N*′,*N*′-tetraisopropylphosphorodiamidite (76 μl, 0.242 mmol), and the reaction mixture was stirred overnight at room temperature. The solution was diluted with AcOEt and washed with brine. The organic layer was dried over Na_2_SO_4_ and evaporated under reduced pressure. The residue was purified by silica gel column chromatography (Hexane:AcOEt = 1:1 to 1:2) to afford compound **21** (74%, 128 mg, 0.140 mmol) as a pale yellow foam.

^31^P NMR (CDCl_3_, 162 MHz) δ 148.6, 148.; HRMS (ESI-TOF) calcd. for C_58_H_69_N_7_O_9_PSi^+^ [M+H]^+^: 1066.4658, found: 1066.4574

**Scheme S3**. Synthesis of fully-deprotected ^O^Pu, ^N^Pu, ^O^Pz, ^N^Pz nucleosides.

**2'-Deoxy-5'-*O*-(4,4'-dimethoxytrityl)-6-(2-pyridon-5-yl)ethynyl-*β*-D-purineriboside (S10)**


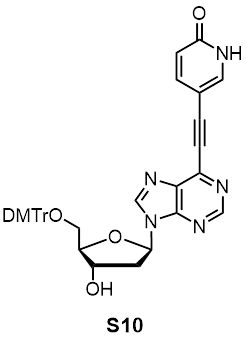
To a solution of compound **3** (100 mg, 0.132 mmol) in THF (1.3 ml) was added tetrabutylammonium fluoride (1.0 M in THF, 324 μl, 0.264 mmol), and the mixture was stirred 7 h at 40 °C. The reaction mixture was quenched by addition of methoxytrimethylsilane (80 μl) and concentrated under reduced pressure. The residue was purified by silica gel column chromatography (CH_2_Cl_2_:CH_3_OH: 60:1 to 20:1 to 10:1 with 0.01% of triethylamine) to afford compound **S10** (94%, 83 mg, 0.124 mmol) as a pale orange powder.

^1^H NMR (400 MHz, CD_3_OD) δ 8.80 (1H, s), 8.70 (1H, s), 8.11 (1H, dd, *J* = 0.8, 2.4 Hz), 7.86 (1H, dd, *J* = 2.4, 9.6 Hz), 7.39-7.36 (2H, m), 7.26-7.18 (7H, m), 6.80-6.76 (4H, m), 6.63-6.58 (2H, m), 4.74 (1H, ddd, *J* = 3.2, 4.0, 6.0 Hz), 4.63 (1H, brs), 4.22 (1H, ddd, *J* = 3.8, 4.0, 4.8 Hz), 3.78 (6H, s), 3.43-3.39 (2H, m), 3.15 (1H, ddd, *J* = 6.0, 6.4, 13.8 Hz), 2.60 (1H, ddd, *J* = 4.4, 6.6, 13.8 Hz); ^13^C NMR (151 MHz, CDCl_3_) δ 163.6, 158.7, 152.6, 151.1, 144.5, 144.4, 143.7, 140.6, 135.7, 135.7, 134.4, 130.1, 128.2, 128.0, 127.1, 120.9, 113.3, 101.5, 94.6, 86.8, 86.6, 85.6, 84.9, 72.5, 63.8, 55.4, 53.6, 40.2; HRMS (ESI-TOF) calcd. for C_38_H_34_N_5_O_6_^+^ [M+H]^+^: 656.2504, found: 656.2518.

**2'-Deoxy-6-(2-pyridon-5-yl)ethynyl-*β*-D-purineriboside (S11)**


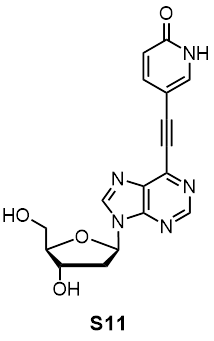
To a solution of compound **S10** (20 mg, 0.031 mmol) in CH_2_Cl_2_ (0.6 ml) was added a solution of dichloroacetic acid (1% in CH_2_Cl_2_, 0.6 ml), and the reaction mixture was stirred at room temperature for 2 h. The reaction mixture was concentrated under reduced pressure. The residue was triturated with cold solution of CH_2_Cl_2_-CH_3_OH (20:1) to afford compound **S11** (82%, 9 mg, 0.025 mmol) as a white solid.

^1^H NMR (400 MHz, CD_3_OD) δ 8.91 (1H, s), 8.88 (1H, s), 8.10 (1H, dd, *J =* 0.8, 2.4 Hz), 7.86 (1H, dd, *J* = 2.4, 9.6 Hz), 6.63-6.60 (2H, m), 4.69-4.63 (1H, m), 4.10 (1H, dd, *J* = 3.6, 6.8 Hz), 3.80 (1H, dd, *J* = 3.6, 12.2 Hz), 3.79 (1H, dd, *J =* 4.0, 12.2 Hz), 2.90 (1H, ddd, *J* = 6.4, 6.8, 14.4 Hz), 2.55 (1H, ddd, *J* = 3.6, 6.4, 14.4 Hz); ^13^C NMR (151 MHz CD_3_OD) δ 164.4, 153.3, 152.2, 147.2, 144.8, 142.9, 142.0, 135.3, 121.4, 102.3, 96.5. 89.7, 85.5, 72.5, 63.1, 41.4; HRMS (ESI-TOF) calcd. for C_17_H_16_N_5_O_4_^+^ [M+H]^+^: 354.1197, found: 354.1247.

**2'-Deoxy-6-(2-aminopyrimidin-5-yl)ethynyl-*β*-D-purineriboside (S12)**


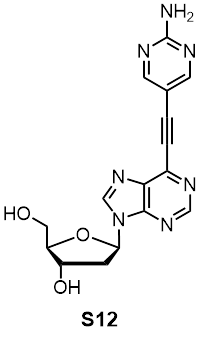
To a solution of compound **6** (60 mg, 0.092 mmol) in CH_2_Cl_2_ (1.8 ml) was added a solution of dichloroacetic acid (1% in CH_2_Cl_2_, 1.8 ml), and the reaction mixture was stirred at room temperature for 30 min. The reaction mixture was concentrated under reduced pressure. The residue was triturated with cold solution of CH_2_Cl_2_-CH_3_OH (50:1) to afford compound **S12** (78%, 25 mg, 0.071 mmol) as a white solid.

^1^H NMR (400 MHz, CD_3_OD) δ 8.81 (1H, s), 8.78 (1H, s), 8.58 (2H, s), 6.52 (1H, t, *J* = 5.2 Hz), 4.56 (2H, ddd, *J* = 2.6, 2.8, 4.8 Hz), 4.00 (1H, dd, *J* = 2.8, 4.8 Hz), 3.77 (1H, dd, *J* = 2.8, 9.8 Hz), 3.70 (1H, dd, *J* = 3.2, 9.8 Hz), 2.81 (1H, ddd, *J* = 5.2, 5.4, 10.8 Hz), 2.45 (1H, ddd, *J* = 2.8, 5.2, 10.8 Hz); ^13^C NMR (151 MHz, CD_3_OD) δ 163.9, 163.0, 153.4, 152.2, 147.1, 142.1, 135.2, 106.4, 95.9, 89.7, 88.0, 86.6, 79.4, 79.2, 79.0, 72.5, 63.1, 41.4, 30.7; HRMS (ESI-TOF) calcd. for C_16_H_16_N_7_O_3_^+^ [M+H]^+^: 354.1309, found: 354.1305.

**5-(2-Pyridon-6-yl)ethynyl-3-[5'-*O*-(4,4'-dimethoxytrityl)-2'**-**deoxy**-***β***-**D**-**ribofuranos**-**1**-**yl]pyridazinone (S13)**


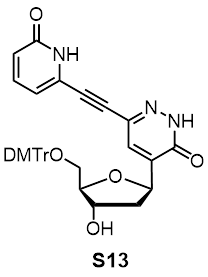
To a solution of compound **16** (10 mg, 0.012 mmol) in THF (0.1 ml) was added tetrabutylammonium fluoride (1.0 M in THF, 60 μl, 0.060 mmol), and the mixture was stirred overnight at 40 °C. The reaction was quenched by methoxytrimethylsilane (80 μl) and concentrated under reduced pressure. The residue was purified by column chromatography (CH_2_Cl_2_:CH_3_OH: 50:1 to 20:1 to 15:1) to afford compound **S13** (92%, 7 mg, 0.021 mmol) as a white solid.

^1^H NMR (400 MHz, CDCl_3_) δ 7.72 (1H, d, *J* = 1.2 Hz ), 7.45-7.33 (2H, m), 7.37-7.33 (6H, m), 7.26 (3H, t, *J* = 8.0 Hz), 7.17 (1H, t, *J* = 7.2 Hz), 6.81 (4H, d, *J* = 8.8 Hz), 6.64 (1H, *J* = 9.2 Hz), 6.39 (1H, d, *J* = 6.8 Hz), 5.33-5.29 (1H, m), 4.43 (1H, t , *J* = 2.0 Hz), 4.23 (1H, d, *J* = 2.0 Hz), 3.74 (6H, s), 3.27 (1H, dd, *J* = 4.0, 10.2 Hz), 3.21 (1H, dd, *J* = 4.8, 10.2 Hz), 2.80 (1H, dd, *J* = 6.8, 12.0 Hz); ^13^C NMR (151 MHz, CDCl_3_) δ 207.2, 164.4, 161.0, 158.7, 145.0, 144.9, 140.9, 136.1, 136.0, 132.0, 130.2, 130.2, 129.2, 128.2, 128.0, 127.0, 113.3, 86.4, 86.4, 74.7, 73.9, 64.5, 55.4, 41.0, 31.1, 29.8, 29.8, 22.8, 14.3, 2.0, 0.1; HRMS (ESI-TOF) calcd. for C_37_H_34_N_3_O_7_^+^ [M+H]^+^: 632.2391; found: 632.2384.

**5-(2-Pyridon-6-yl)ethynyl-3-(2'**-**deoxy**-***β***-**D**-**ribofuranos**-**1**-**yl)pyridazinone (S14)**


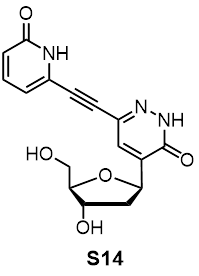
To a solution of compound **S13** (12 mg, 0.019 mmol) in CH_2_Cl_2_ (0.4 ml) was added a solution of dichloroacetic acid (1% in CH_2_Cl_2_, 0.4 ml), and the reaction mixture was stirred at room temperature for 15 min. The reaction mixture was concentrated under reduced pressure. The residue was triturated with cold solution of CH_2_Cl_2_-CH_3_OH (100:1) to afford compound **S14** (80%, 5 mg, 0.015 mmol) as a white powder.

^1^H NMR (400 MHz, CD_3_OD) δ 7.65 (1H, d, *J* = 1.2 Hz ), 7.46 (1H, dd, *J* = 7.2, 9.2 Hz), 6.61 (1H, d, *J* = 7.2 Hz), 6.51 (1H, d, *J* = 9.2 Hz), 5.05 (1H, dd, *J* = 6.0, 9.6 Hz), 4.20-4.19 (1H, m), 3.87 (1H, dd, *J* = 4.8, 7.4 Hz), 3.62-3.53 (2H, m), 2.39 (1H, ddd, *J* = 2.4, 6.0, 13.0 Hz), 2.45 (1H, ddd, *J* = 6.0, 8.4, 13.0 Hz); ^13^C NMR (151 MHz, CD_3_OD) δ 165.5, 161.4, 145.6, 142.6, 132.8, 130.4, 129.5, 122.8, 114.2, 89.0, 75.8, 74.0, 63.8, 41.9; HRMS (ESI-TOF) calcd. for C_16_H_16_N_3_O_5_^+^ [M+H]^+^: 330.1084, found: 330.1098.

**5-(2-Aminopyrimidin-6-yl)ethynyl-3-[5'-*O*-(4,4'-dimethoxytrityl)-2'**-**deoxy**-***β***-**D**-**ribofuranos**-**1**-**yl]pyridazinone (S15)**


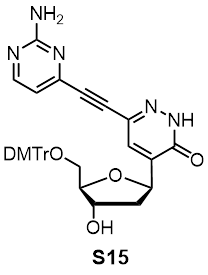
To a solution of compound **19** (25 mg, 0.034 mmol) in THF (0.34 mL) was added tetrabutylammonium fluoride (1.0 M in THF, 51 μl, 0.051 mmol), and the mixture was stirred for 1 h at 40 °C. The reaction was quenched by methoxytrimethylsilane (35 μl) and concentrated under reduced pressure. The residue was purified by column chromatography (CH_2_Cl_2_:CH_3_OH: 50:1 to 30:1 to 20:1 to 15:1 with 0.01% of triethylamine) to afford compound **S15** (88%, 19 mg, 0.03 mmol) as a white solid.

^1^H NMR (400 MHz, CDCl_3_) δ 8.86 (1H, s), 8.41 (1H, d, *J* = 1.4 Hz), 8.27 (1H, s), 8.03 (1H, d, *J* = 1.4 Hz), 7.40-7.38 (2H, m), 7.30-7.21 (7H, m), 6.81-6.79 (4H, m), 6.51 (1H, t, *J* = 6.4 Hz), 4.73-4.70 (1H, m), 4.17 (1H, dd, *J* = 4.8, 8.6 Hz), 3.44 (1H, dd, *J* = 4.8, 10.0 Hz), 3.39 (1H, dd, *J* = 5.2, 10.0 Hz), 2.90 (1H, dt, *J* = 6.4, 14.0 Hz), 2.60 (1H, ddd, *J* = 2.4, 6.4, 14.0 Hz), 2.08 (1H, d, *J* = 3.6 Hz); ^13^C NMR (151 MHz, CD_3_OD) δ 164.6, 161.3, 160.1, 160.1, 160.0, 151.3, 146.4, 146.4, 137.4, 137.3, 132.8, 131.3, 131.2, 130.4, 129.3, 128.9, 127.9, 114.3, 114.2, 88.6, 87.6, 87.1, 75.6, 74.1, 65.3, 55.7, 41.8; HRMS (ESI-TOF) calcd. for C_36_H_34_N_5_O_6_^+^ [M+H]^+^: 632.2504, found: 632.2504.

**5-(2-Aminopyrimidin-6-yl)ethynyl-3-(2'**-**deoxy**-***β***-**D**-**ribofuranos**-**1**-**yl)pyridazinone (S16)**


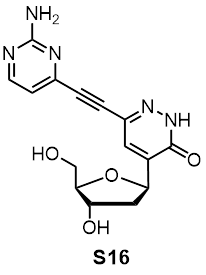
To a solution of compound **S15** (10 mg, 0.016 mmol) in CH_2_Cl_2_ (0.3 ml) was added a solution of dichloroacetic acid (1% in CH_2_Cl_2_, 0.3 ml), and the reaction mixture was stirred at room temperature for 1 min. The reaction mixture was concentrated under reduced pressure. The residue was triturated with cold solution of CH_2_Cl_2_-CH_3_OH (20:1) to afford compound **S16** (76%, 4mg, 0.012 mmol) as a white solid.

^1^H NMR (400 MHz, CD_3_OD) δ 8.31 (1H, d, *J =* 5.0 Hz), 7.76 (1H, d, *J* = 1.4 Hz), 6.84 (1H, d, *J* = 5.0 Hz), 5.14 (1H, dd, *J* = 6.1, 9.6 Hz), 4.31-4.28 (1H, m), 3.71-3.62 (1H, m), 3 69 (1H, dd, *J* = 4.2, 12.0 Hz), 3.67 (1H, dd, *J* = 5.0, 12.0 Hz), 2.48 (1H, ddd, *J* = 2.2, 6.1, 13.1 Hz), 1.83 (1H, ddd, *J* = 3.1, 6.1, 13.1 Hz); ^13^C NMR (151 MHz, CD_3_OD) δ 163.8, 161.3, 160.3, 150.5, 145.7, 132.9, 130.6, 114.2, 89.0, 88.3, 87.2, 75.8, 74.0, 70.3, 63.7, 41.9; HRMS (ESI-TOF) calcd. for C_15_H_16_N_5_O_4_^+^ [M+H]^+^: 330.1197, found: 330.1199.

**2. UV absorption spectra of the alkynylated purine and pyridazine nucleosides**


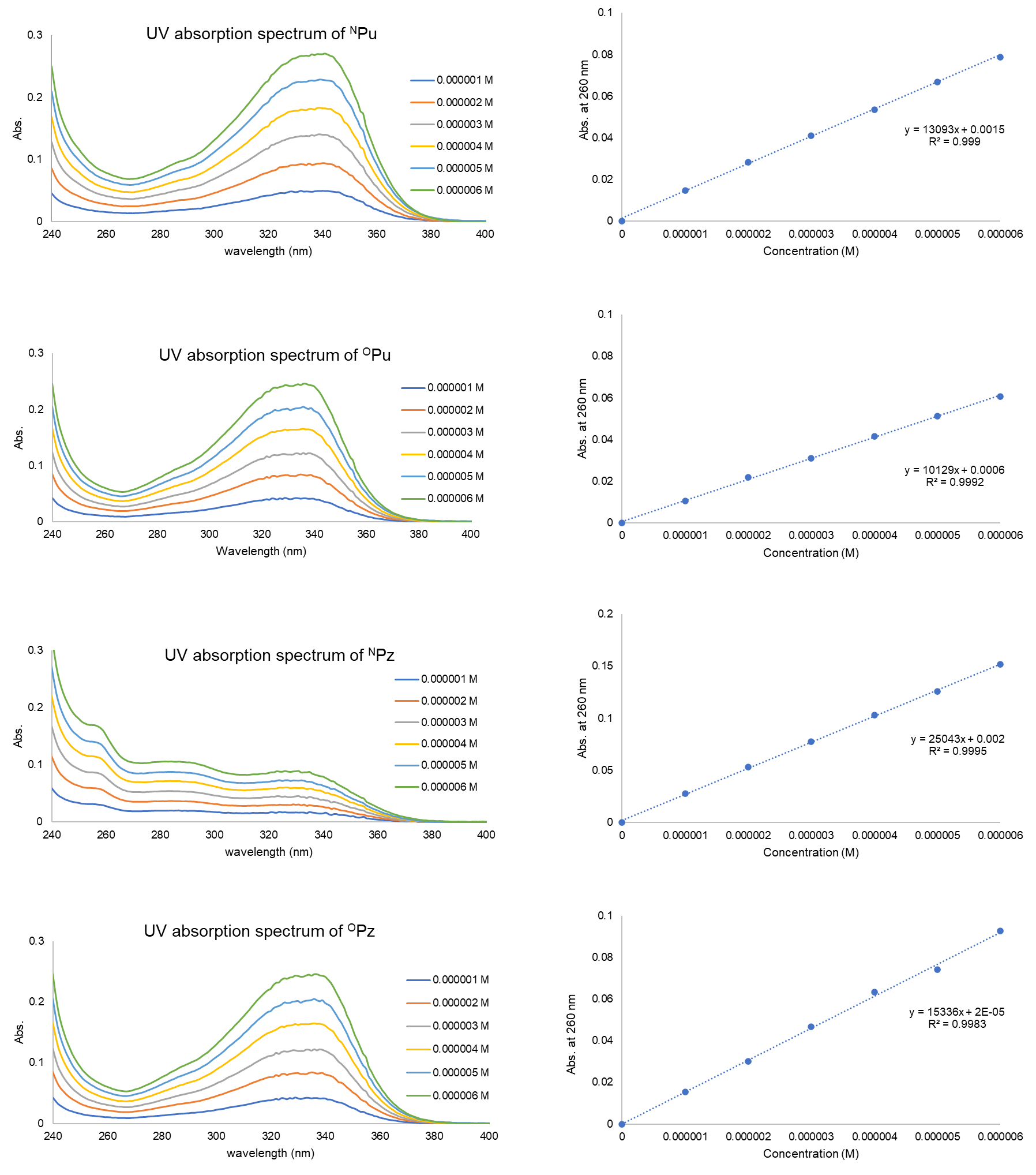


**Figure S1**. UV absorption spectra of ^N^Pu, ^O^Pu, ^N^Pz, ^O^Pz in ddH_2_O.


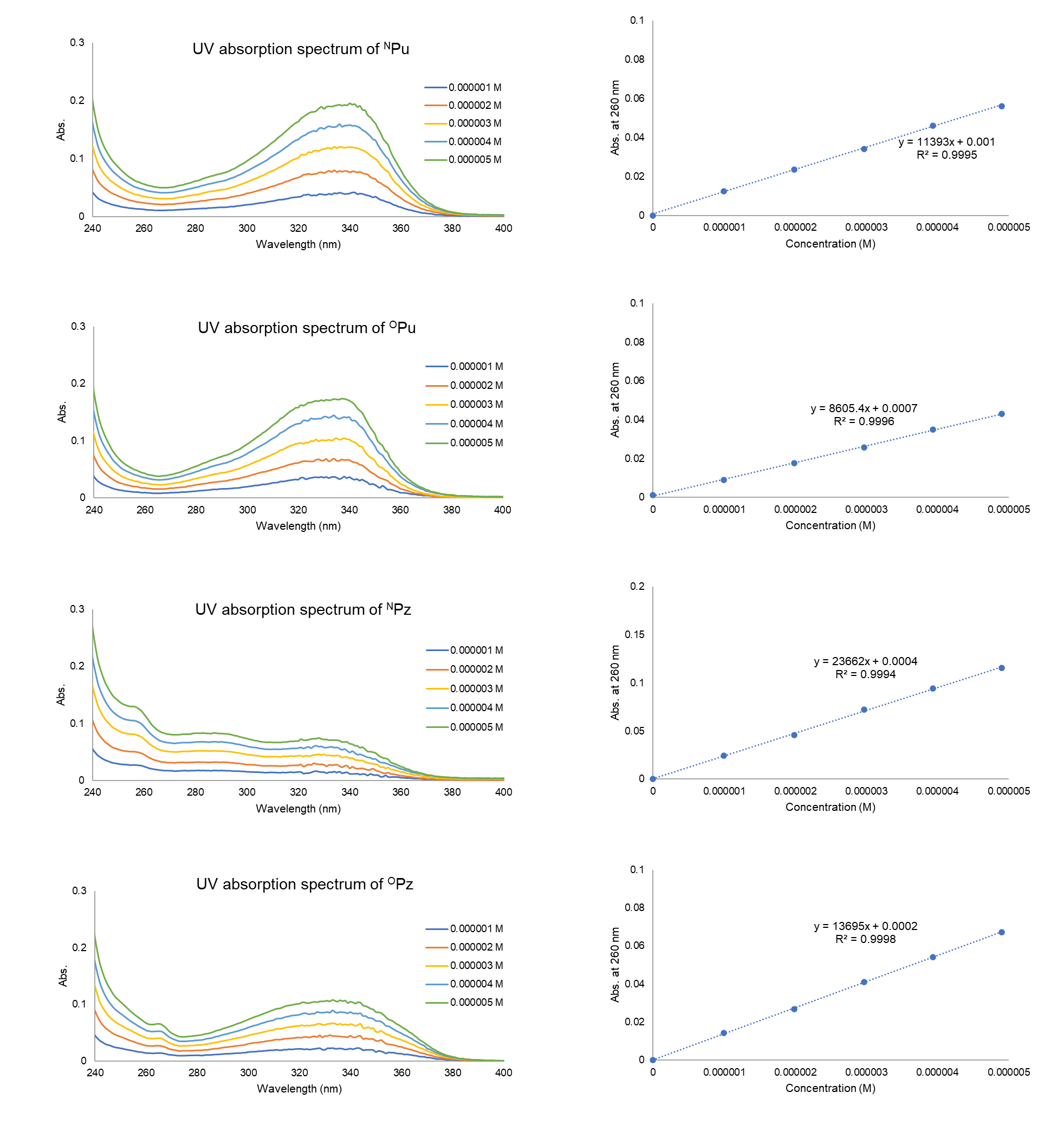


**Figure S2**. UV absorption spectra of ^N^Pu, ^O^Pu, ^N^Pz, ^O^Pz in 10 mM sodium phosphate buffer (pH 7.0).

**3. Sequences and characterization of** **the synthesized oligonucleotides**

**Table S1**. Sequences of the synthesized oligonucleotides and their MALDI-TOF mass data.

| Sequences (5' to 3') | Calcd. [M-H]^-^ | Found (*m*/*z*) |
| --- | --- | --- |
| TCAG**^O^Pu**GTAAG | 3175.55 | 3175.84 |
| TCAG**^N^Pu**GTAAG | 3175.56 | 3175.92 |
| TCAG**^O^Pz**GTAAG | 3151.54 | 3151.44 |
| TCAG**^N^Pz**GTAAG | 3151.55 | 3151.63 |
| CTTAC**^O^Pu**CTGA | 3086.51 | 3086.12 |
| CTTAC**^N^Pu**CTGA | 3086.52 | 3086.80 |
| CTTAC**^O^Pz**CTGA | 3062.50 | 3062.84 |
| CTTAC**^N^Pz**CTGA | 3062.51 | 3062.39 |
| TCA**^N^Pu**G**^N^Pu**TAAG | 3261.58 | 3261.93 |
| TCA**^N^Pu**G**^N^Pu**T**^N^Pu**AG | 3363.59 | 3363.53 |
| TCA**^O^Pu**G**^O^Pu**TAAG | 3261.56 | 3261.96 |
| TCA**^O^Pu**G**^O^Pu**T**^O^Pu**AG | 3363.56 | 3363.08 |
| CTTA**^O^Pz**C**^O^Pz**TGA | 3164.51 | 3164.98 |
| CT**^O^Pz**A**^O^Pz**C**^O^Pz**TGA | 3251.52 | 3251.37 |
| CTT**^N^Pu**C**^N^Pu**CTGA | 3188.53 | 3188.46 |
| CTT**^N^Pu**C**^N^Pu**C**^N^Pu**GA | 3299.56 | 3299.10 |
| CTT**^O^Pu**C**^O^Pu**CTGA | 3188.51 | 3188.12 |
| CTT**^O^Pu**C**^O^Pu**C**^O^Pu**GA | 3299.53 | 3299.31 |
| TCA**^N^Pu**G**^N^Pu**TAAG | 3261.58 | 3261.93 |
| TCA**^N^Pu**G**^N^Pu**T**^N^Pu**AG | 3363.59 | 3363.53 |
| TCAG**^N^Pu^N^Pu**TAAG | 3261.58 | 3261.13 |
| TCA**^N^Pu^N^Pu^N^Pu**TAAG | 3347.60 | 3347.37 |
| TCAG**^O^Pu^O^Pu**TAAG | 3261.56 | 3261.59 |
| TCA**^O^Pu^O^Pu^O^Pu**TAAG | 3347.57 | 3347.52 |
| CTTA**^O^Pz^O^Pz**CTGA | 3164.51 | 3164.02 |
| CTTA**^O^Pz^O^Pz^O^Pz**TGA | 3266.52 | 3266.64 |
| CTTA**^N^Pu^N^Pu**CTGA | 3212.55 | 3212.74 |
| CTTA**^N^Pu^N^Pu^N^Pu**TGA | 3338.58 | 3338.95 |
| CTTA**^O^Pu^O^Pu**CTGA | 3212.53 | 3212.96 |
| CTTA**^O^Pu^O^Pu^O^Pu**TGA | 3338.55 | 3338.89 |
| TCAG**^O^Pz^O^Pz**TAAG | 3213.54 | 3213.05 |
| TCA**^O^Pz^O^Pz^O^Pz**TAAG | 3275.54 | 3275.46 |
| TG**^O^Pz**GGCC**^N^Pu**CA | 3214.53 | 3214.13 |


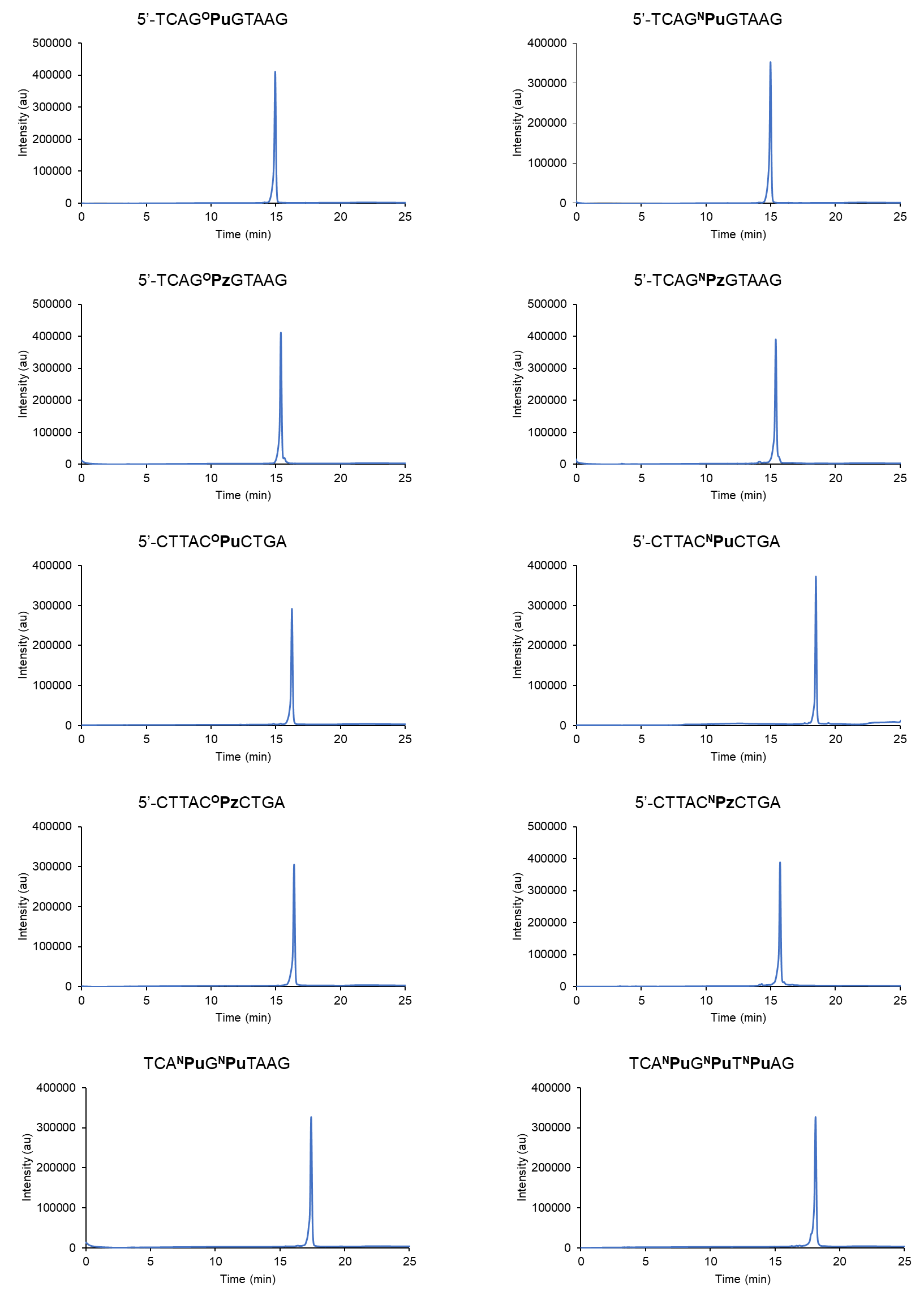


**Figure S3**. Analytical HPLC traces of purified oligonucleotides.


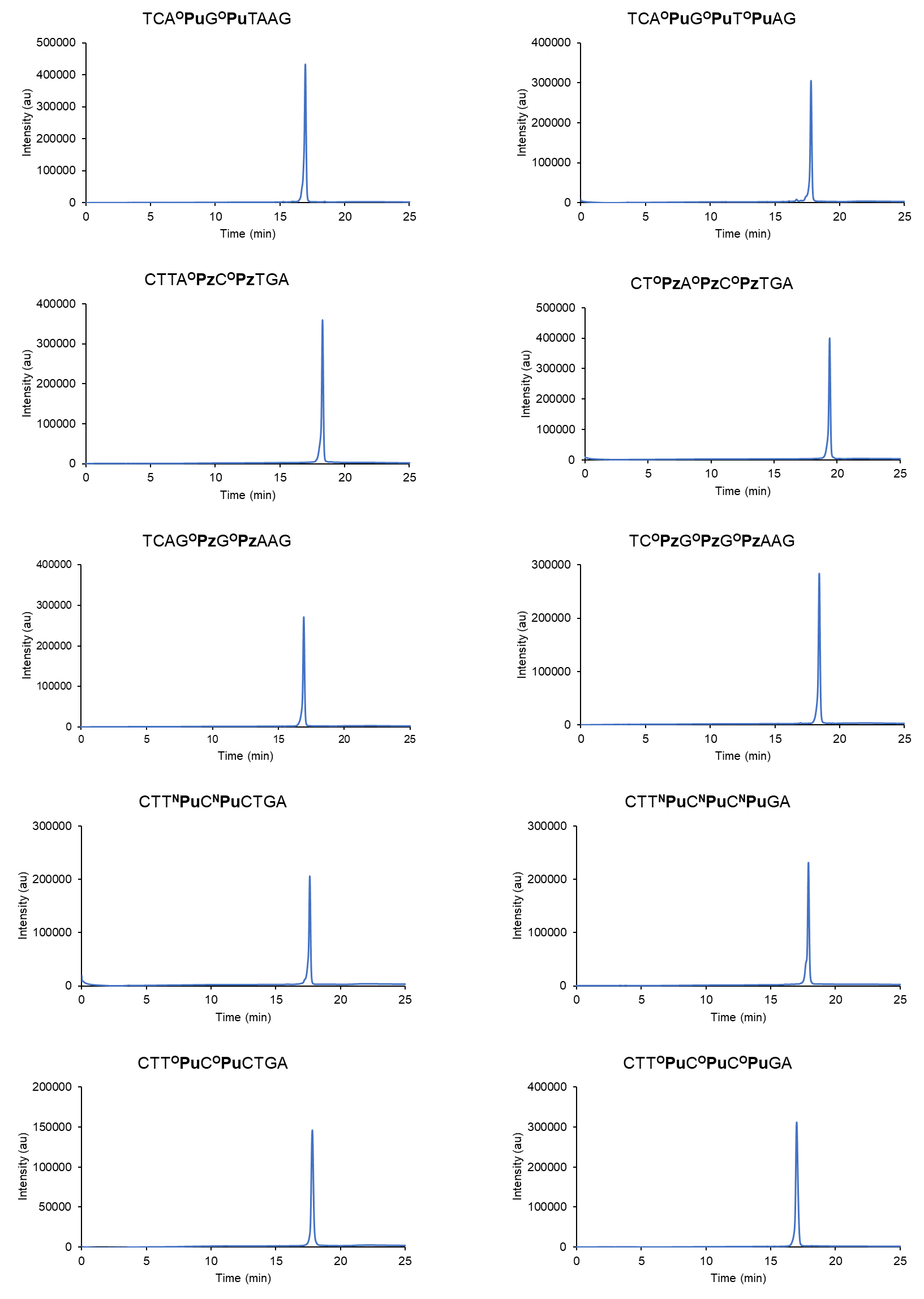


**Figure S3 (continued)**. Analytical HPLC traces of purified oligonucleotides.

**
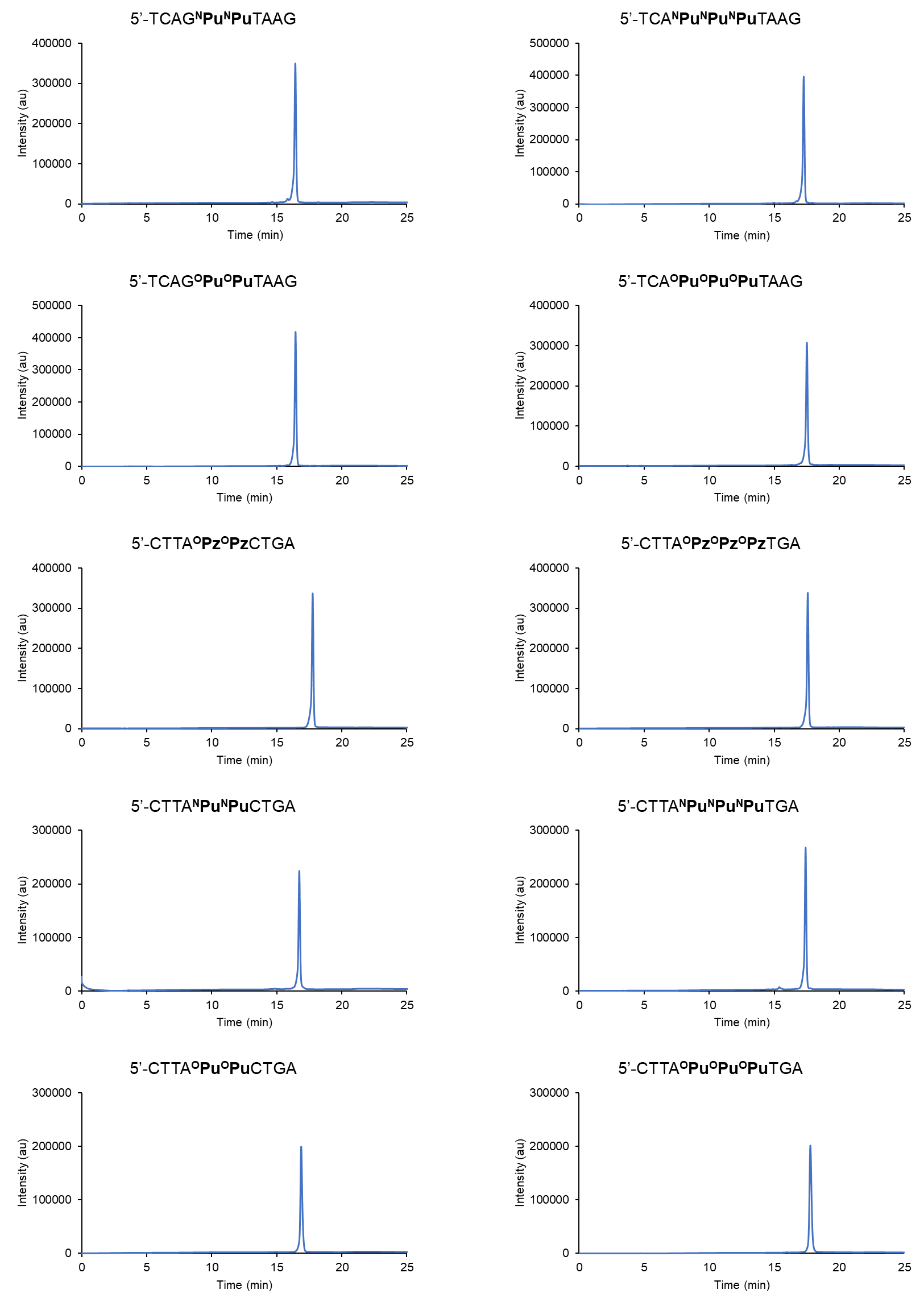
**

**Figure S3 (continued)**. Analytical HPLC traces of purified oligonucleotides.


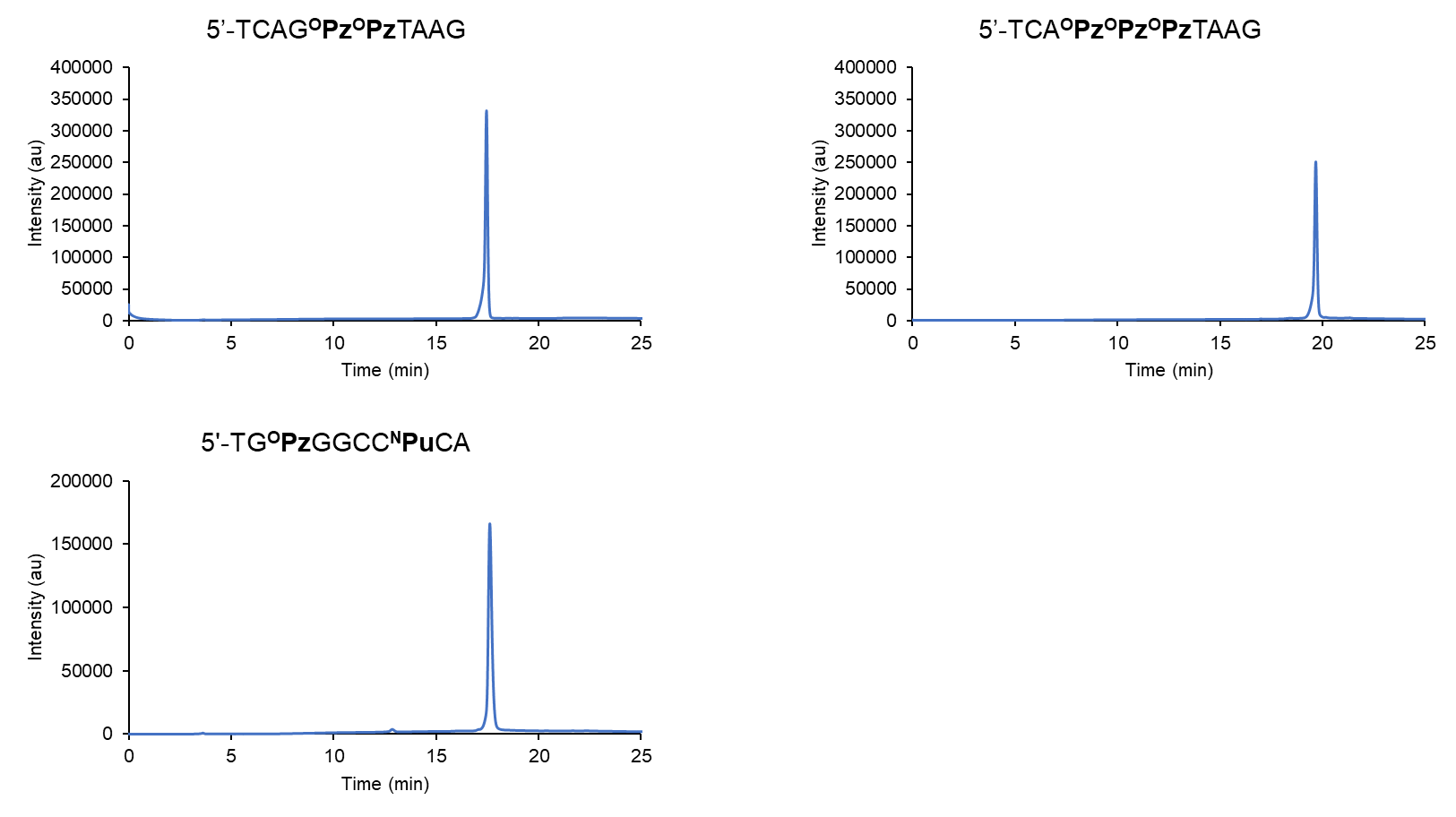


**Figure S3 (continued)**. Analytical HPLC traces of purified oligonucleotides.

**
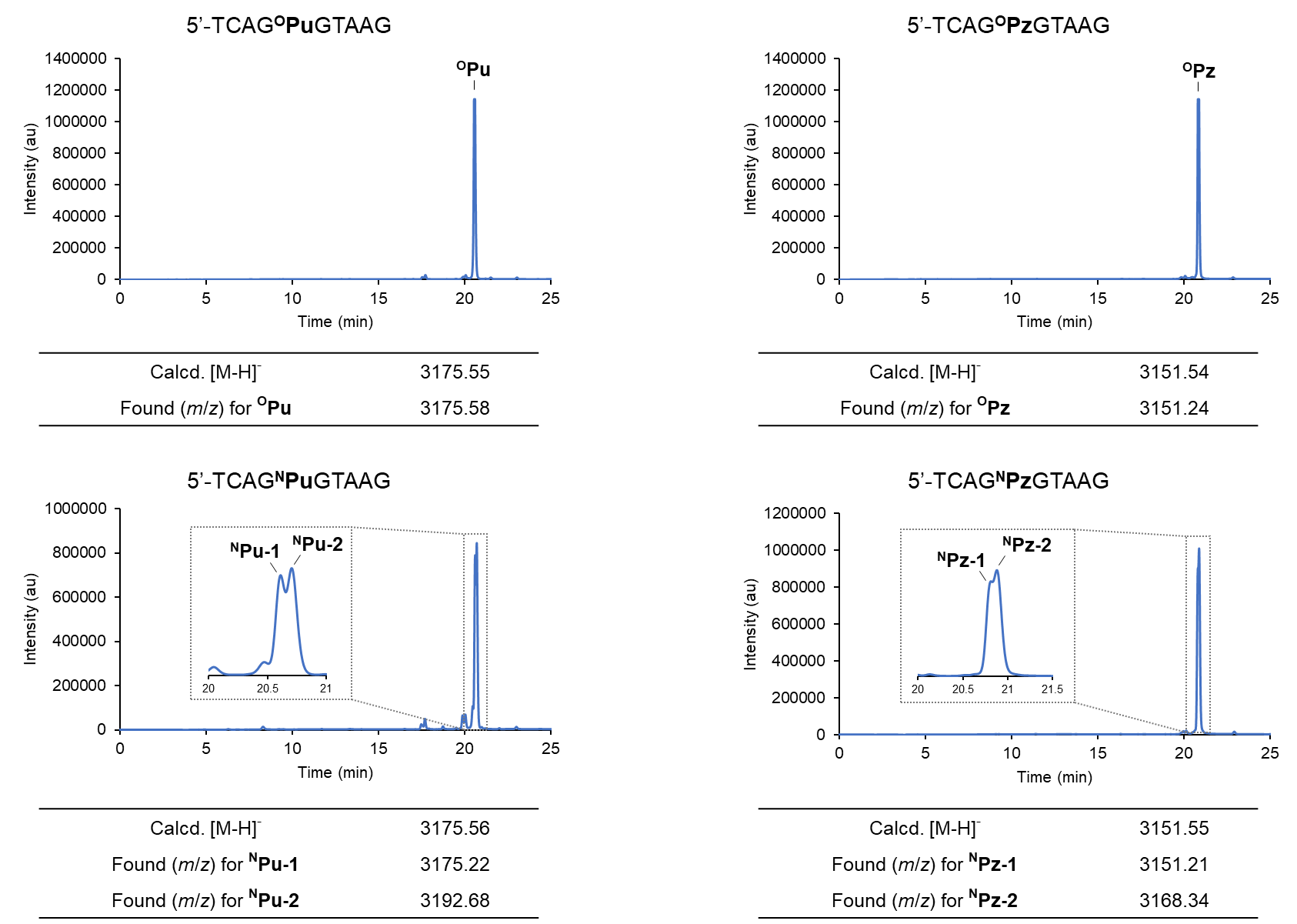
**

**Figure S4**. Analytical HPLC traces and MALDI-TOF MS data of the oligonucleotides incorporating the alkynylated purine and pyridazine nucleosides after the treatment with 28% NH_4_OH at 55 °C. While ^O^Pu and ^O^Pz were intact under the conditions, ^N^Pu and ^N^Pz afforded an additional peak with slightly longer retention time (^N^Pu-2 and ^N^Pz-2, respectively). These oligonucleotides were 17 Da larger than the parental ^N^Pu-1 and ^N^Pz-1.

**4. UV melting analyses of the duplexes containing the alkynylated purines and pyridazines at single position**


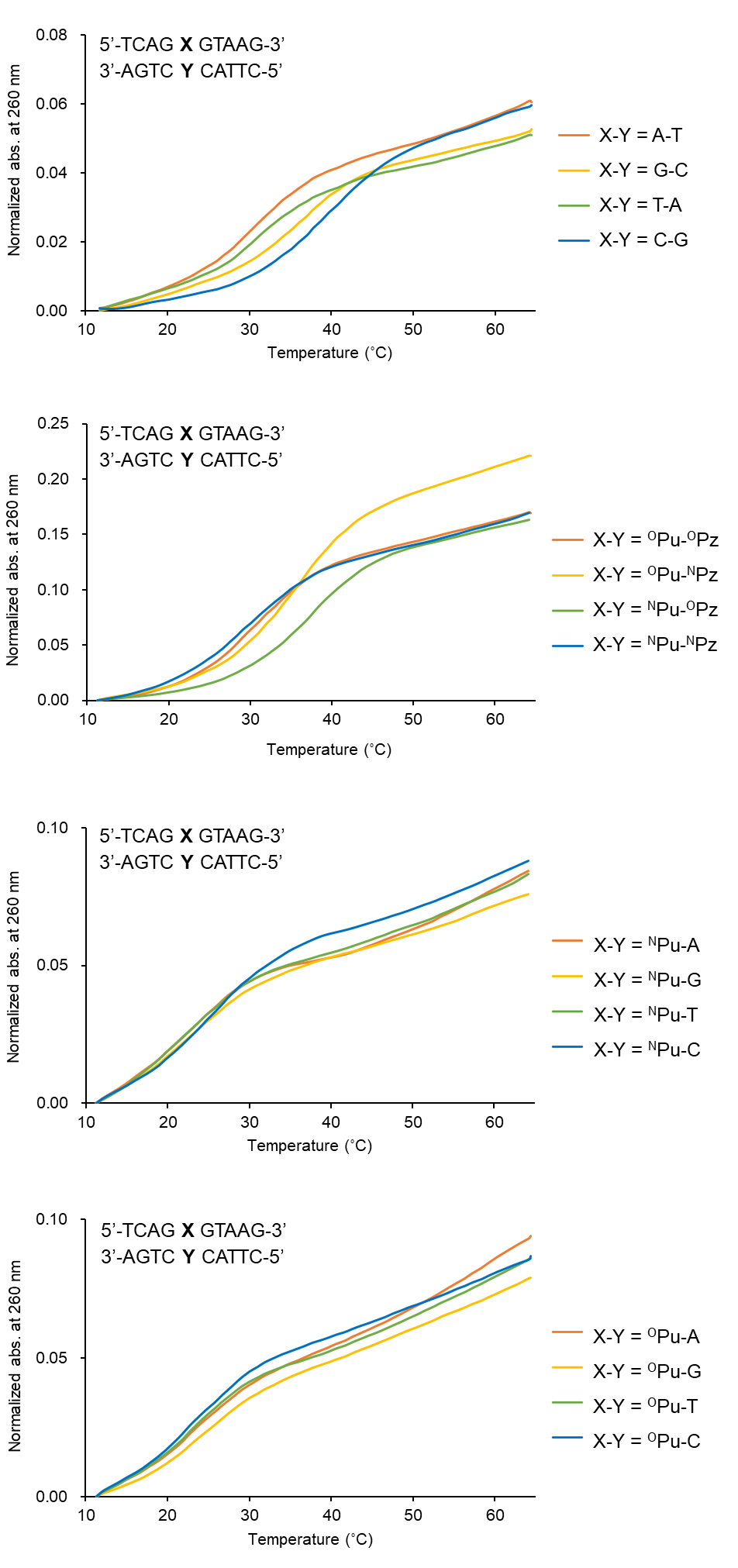


**Figure S5**. UV melting curves of the DNA duplexes with different combinations of the nucleosides at X-Y.


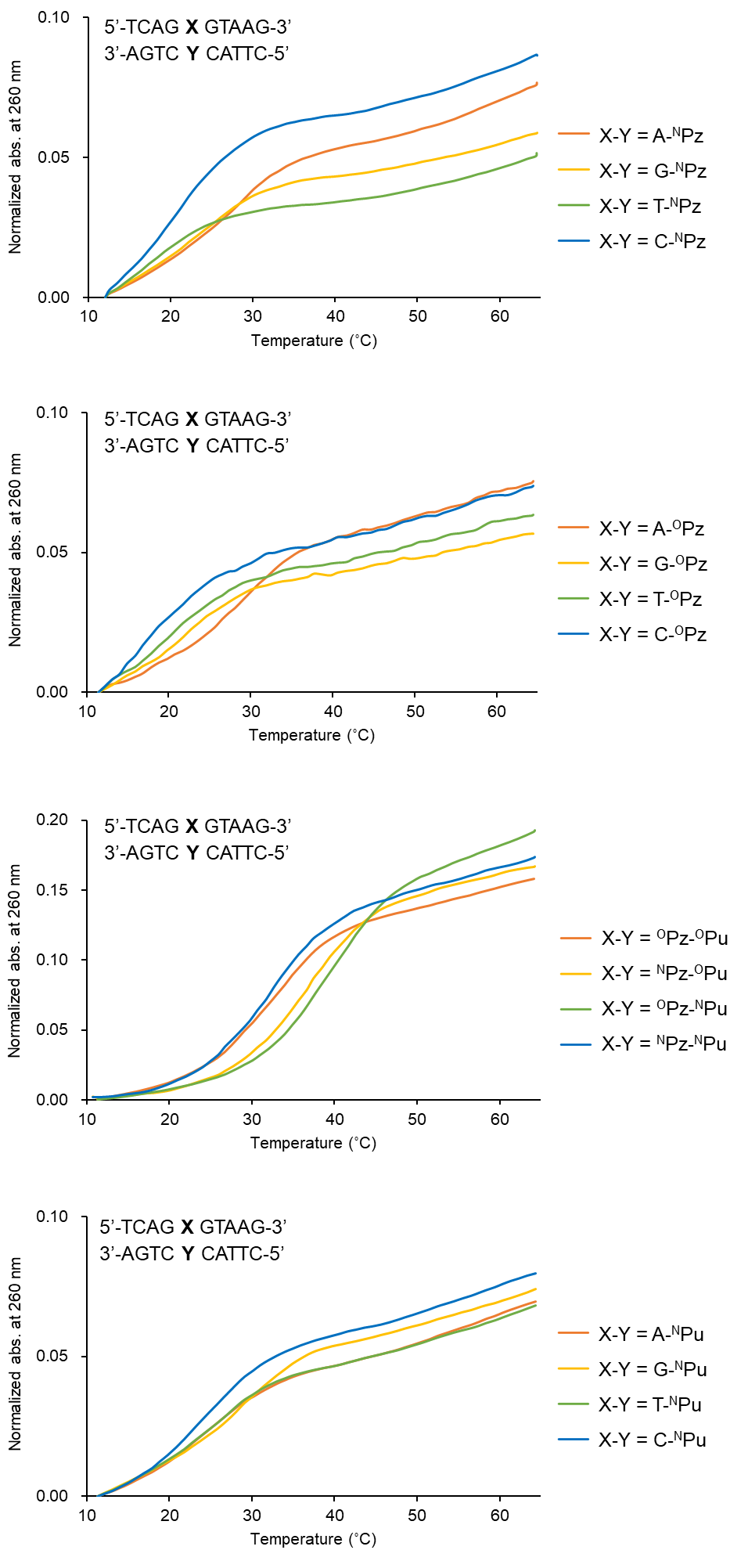


**Figure S5 (continued)**. UV melting curves of the DNA duplexes with different combinations of the nucleosides at X-Y.


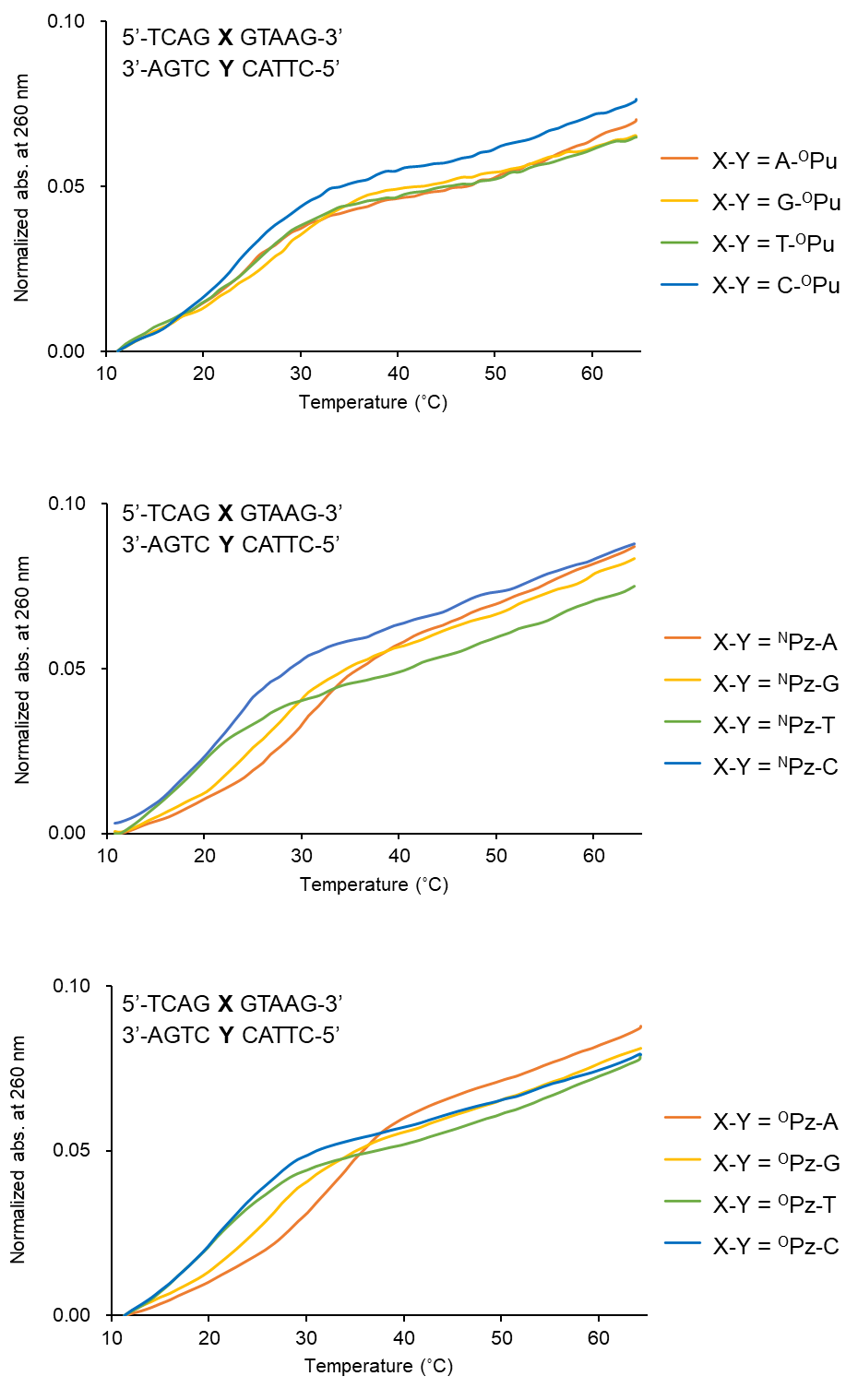


**Figure S5 (continued)**. UV melting curves of the DNA duplexes with different combinations of the nucleosides at X-Y.

**Table S2.** *T*_m_ values of the DNA duplexes featuring different combinations of ^N^Pu, ^O^Pu, ^N^Pz, ^O^Pz and canonical bases.


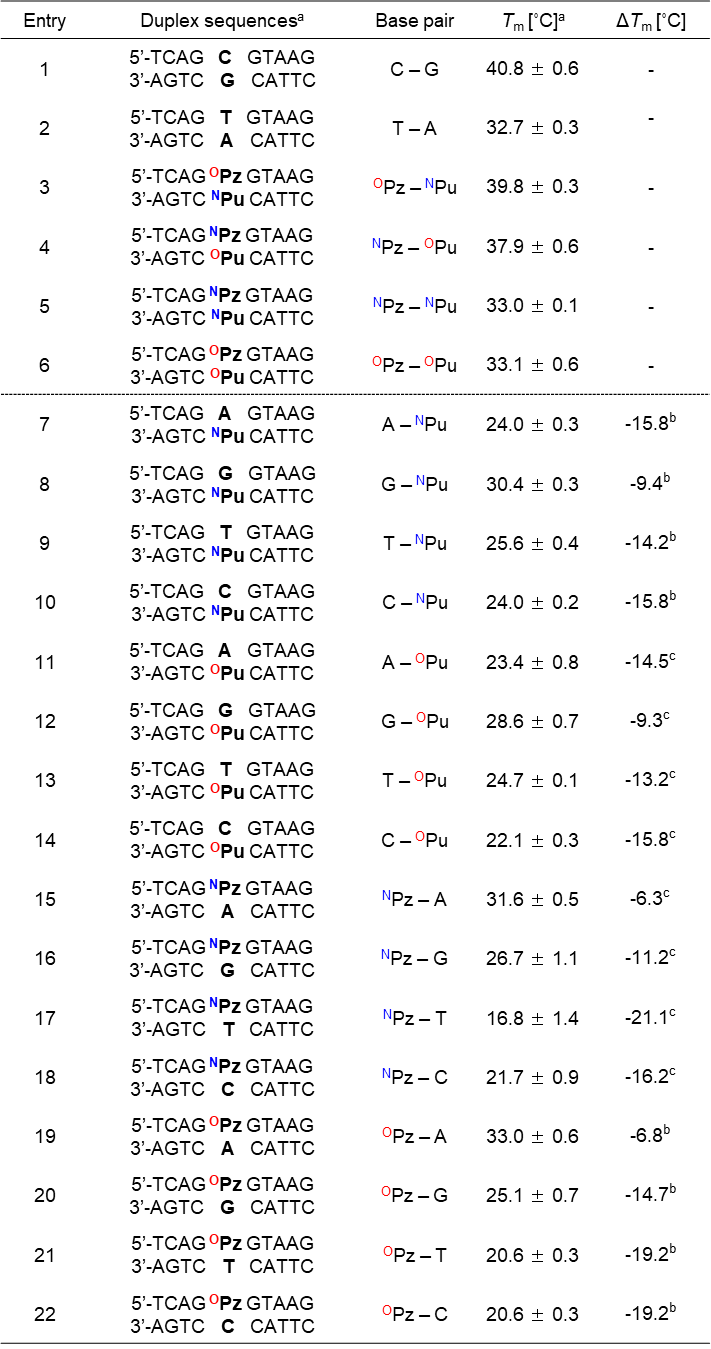


^a^Conditions: 2 μM of each ODN, 150 mM NaCl, 10 mM sodium phosphate buffer, pH 7.0. Each *T*_m_ value is average of three measurements. Δ*T*_m_ values are shown in parentheses. ^b^To duplex with a ^O^Pz-^N^Pu pair (Entry 2). ^c^To duplex with a ^N^Pz-^O^Pu pair (Entry 3).

**5. Thermodynamic analyses of the duplexes containing single alkynylated purine-pyridazine pair**


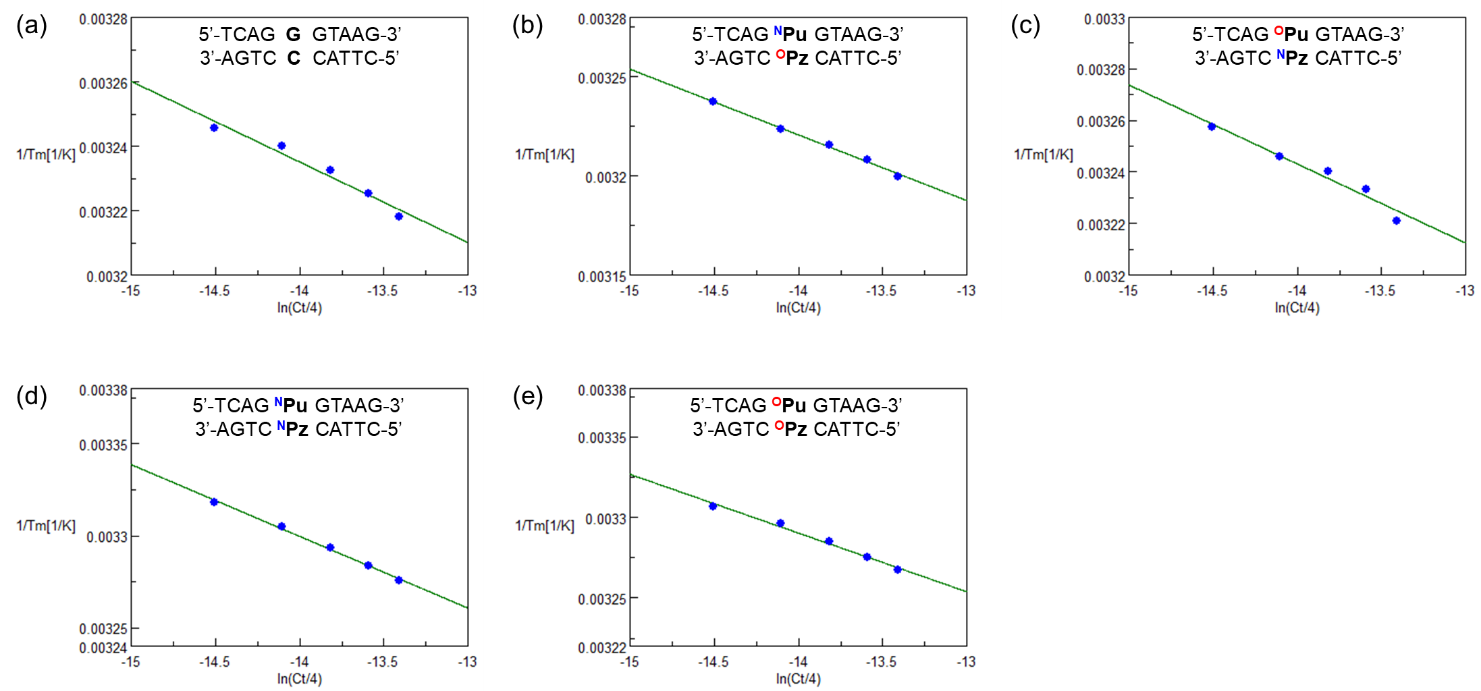


**Figure S6.** Representative Van’t Hoff plots obtained from the UV melting experiments of the DNA duplexes featuring (a) G-C, (b) ^N^Pu-^O^Pz, (c) ^O^Pu-^N^Pz, (d) ^N^Pu-^N^Pz, and (e) ^O^Pu-^O^Pz pairs. Conditions: 1-3 μM of each ODN, 150 mM NaCl, 10 mM sodium phosphate buffer.

**Table S3.** Thermodynamic parameters for the DNA duplexes containing different combinations of ^N^Pu, ^O^Pu, ^N^Pz, ^O^Pz and canonical nucleobases.^a^


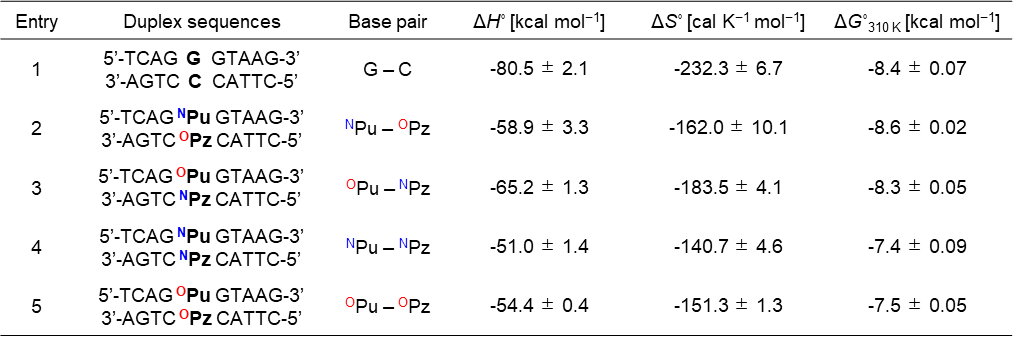


^a^Each thermodynamic parameter was determined from the average of three measurements.

**6. Speculated interaction modes of the alkynylated purines and pyridazines with canonical bases**


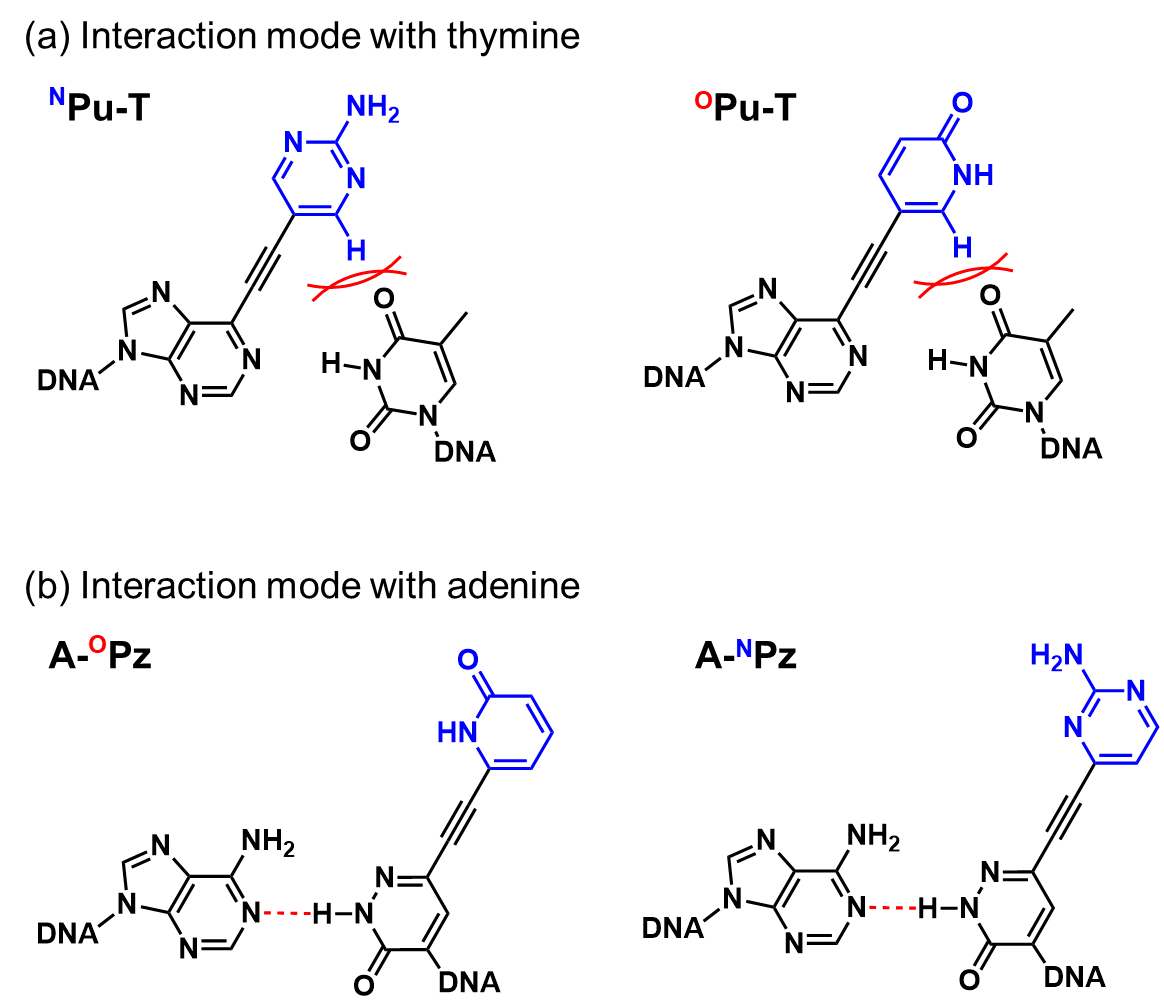


**Figure S7**. Speculated interaction modes of (a) the alkynylated purines (^N^Pu, ^O^Pu) with thymine and (b) the alkynylated pyridazines (^N^Pz, ^O^Pz) with adenine in the DNA duplex.

**7. CD spectra of the duplexes containing single alkynylated purine-pyridazine pair**


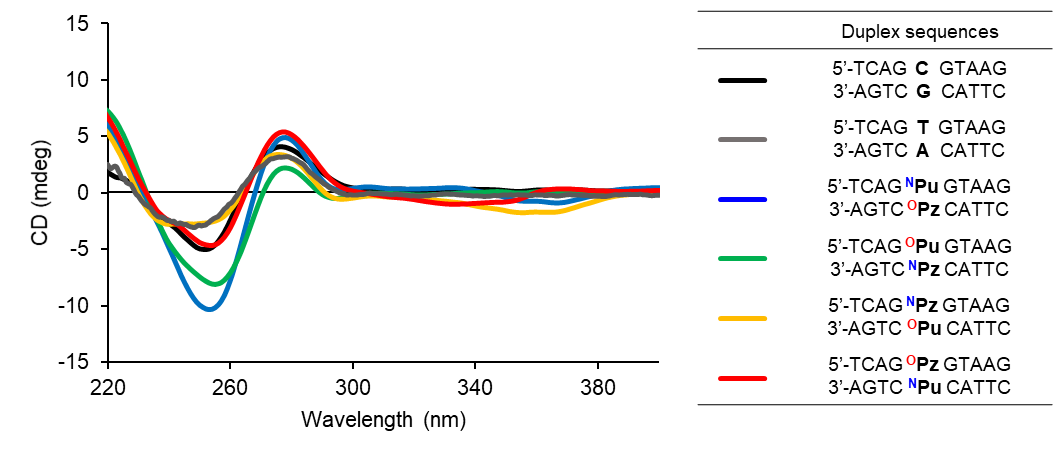


**Figure S8**. CD spectra of the DNA duplexes containing C-G, T-A, ^N^Pu-^O^Pz, ^O^Pu-^N^Pz, ^N^Pz-^O^Pu and ^O^Pz-^N^Pu. Conditions: 2 μM of each ODN, 150 mM NaCl, 10 mM sodium phosphate buffer, pH 7.0 at 20 ˚C.

**8.** **NMR structural studies of the duplex containing ^N^Pu-^O^Pz pairs**


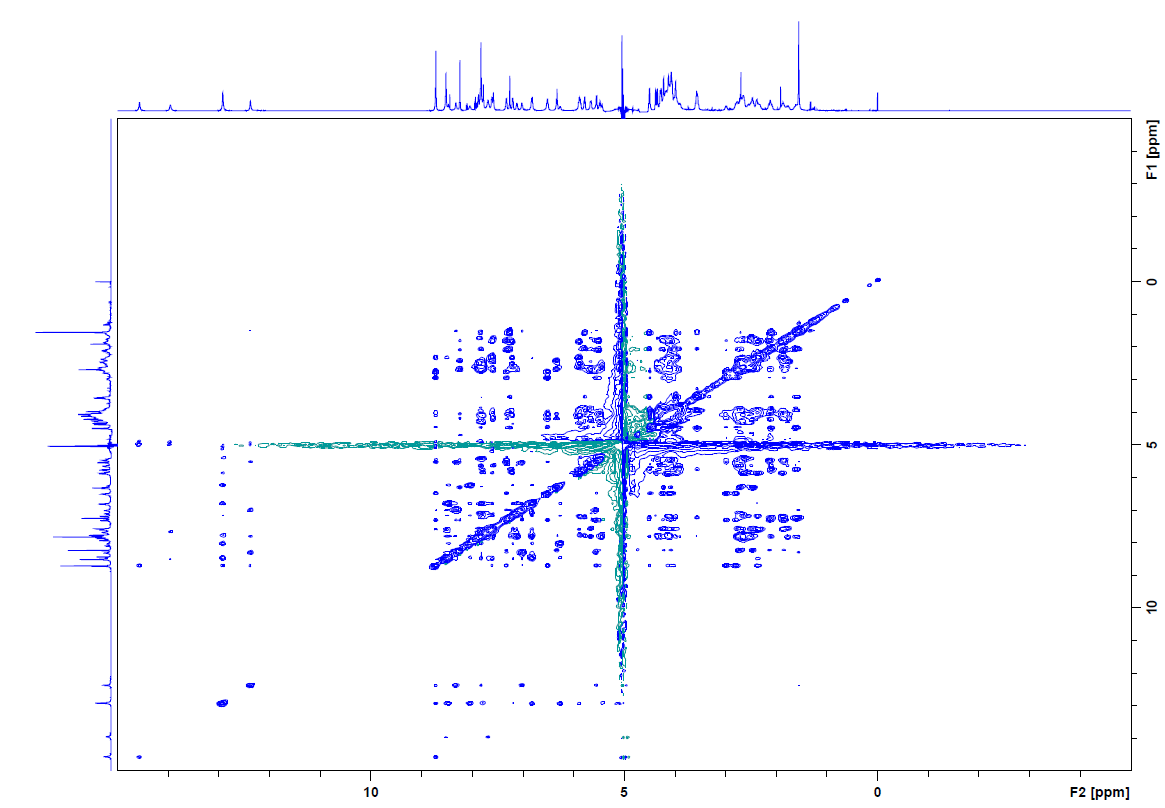


**Figure S9**. ^1^H-^1^H 2D NOESY spectrum of the 10-bp self-complementary DNA duplex (5’-TG^O^PzGGCC^N^PuCA) recorded in H_2_O-D_2_O at 5 °C.


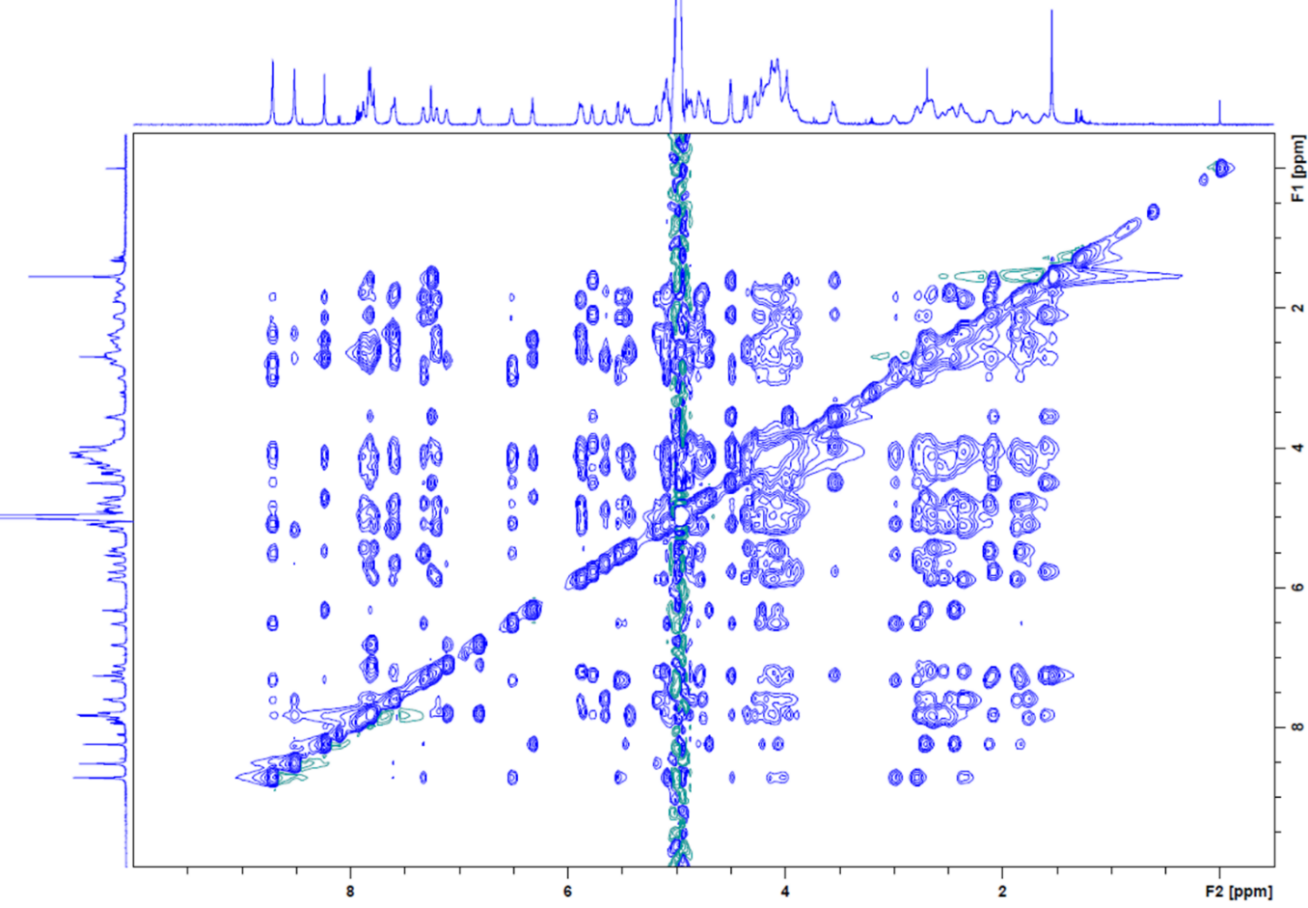


**Figure S10**. ^1^H-^1^H 2D NOESY spectrum of the 10-bp self-complementary DNA duplex (5’-TG^O^PzGGCC^N^PuCA) recorded in D_2_O at 5 °C.


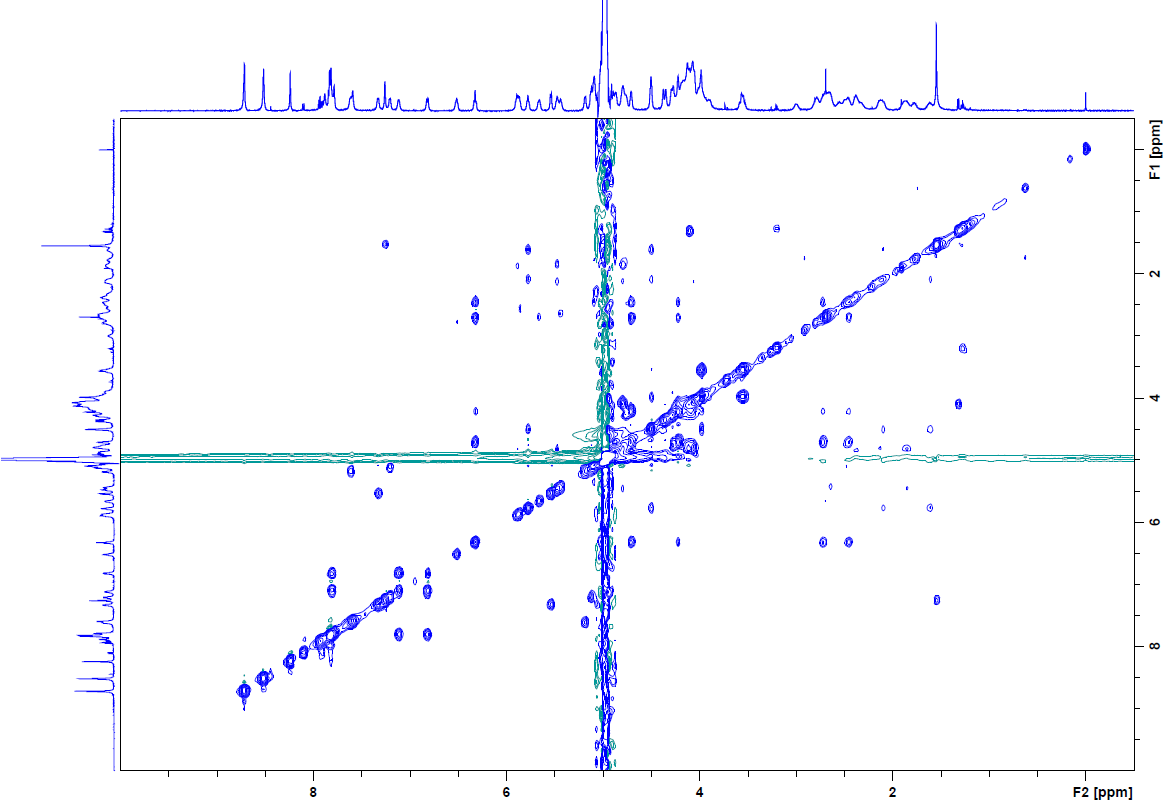


**Figure S11**. ^1^H-^1^H 2D TOCSY spectrum of the 10-bp self-complementary DNA duplex (5’-TG^O^PzGGCC^N^PuCA) recorded in D_2_O at 5 °C.


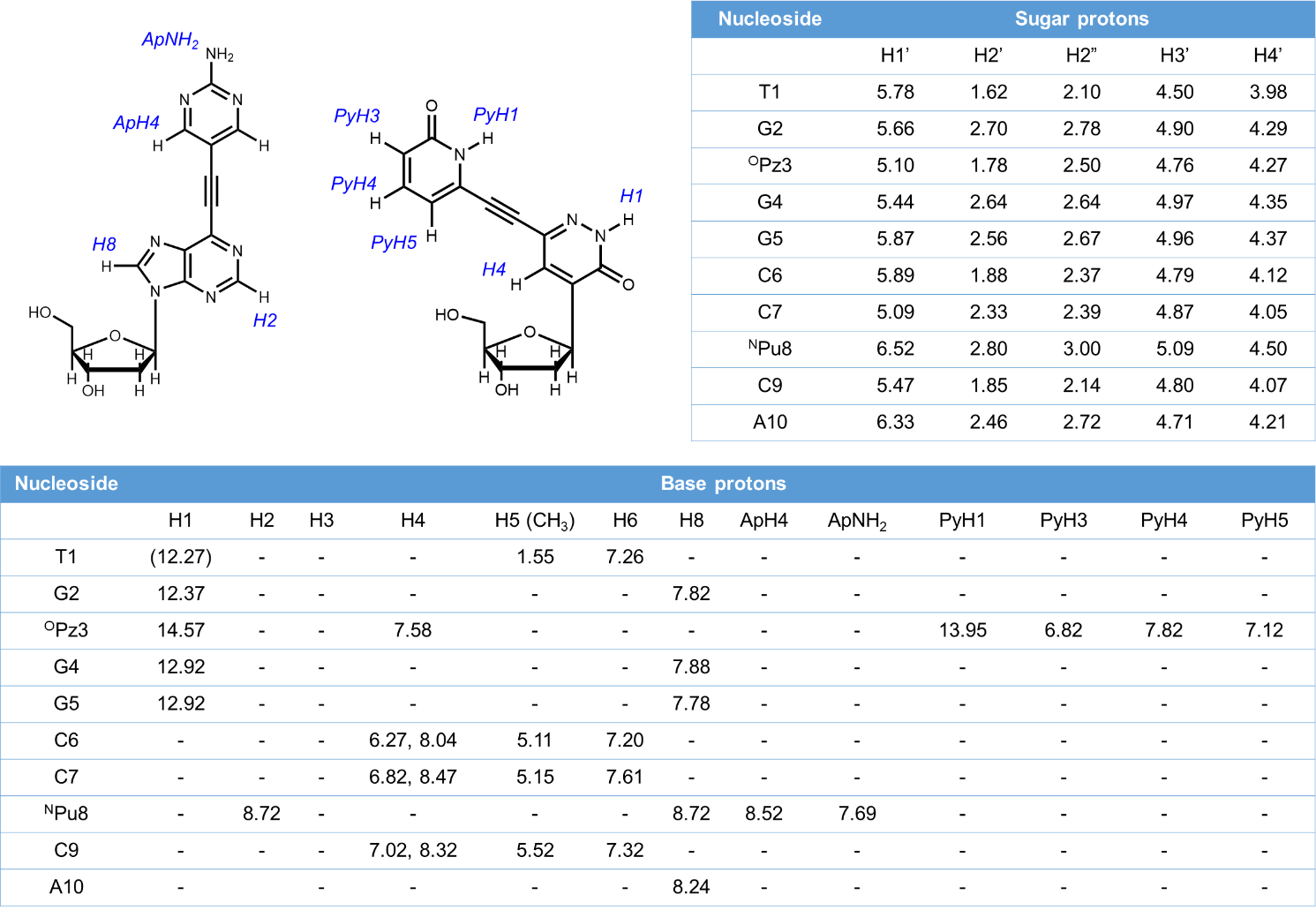


**Figure S12**. Assignment of ^1^H chemical shifts of the 10-bp self-complementary DNA duplex (5’-TG^O^PzGGCC^N^PuCA).


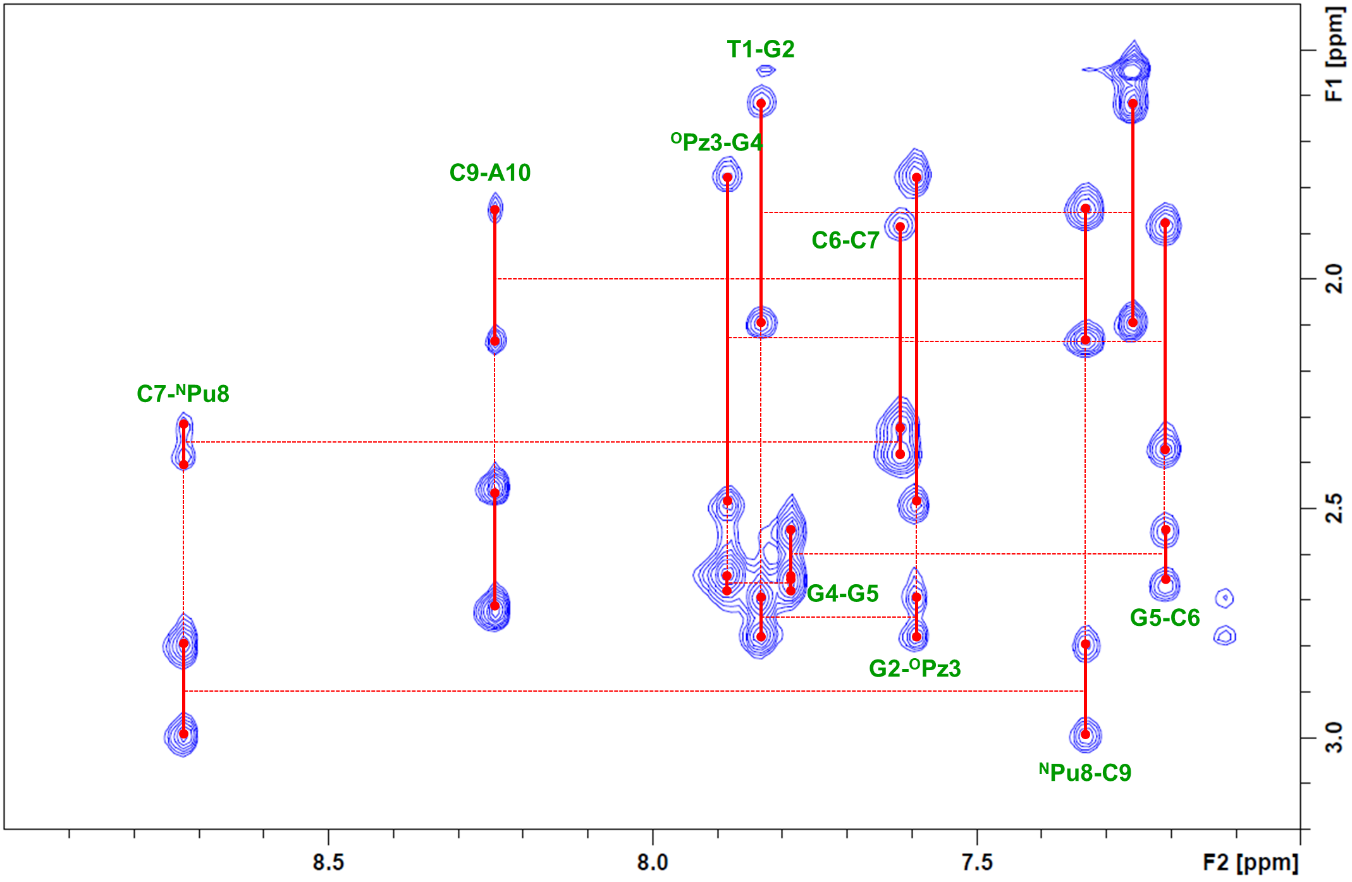


**Figure S13**. An excerpt of NOESY spectrum showing the connectivity of H2’/2” and base protons through the strand. The spectrum was measured in D_2_O at 5 °C.


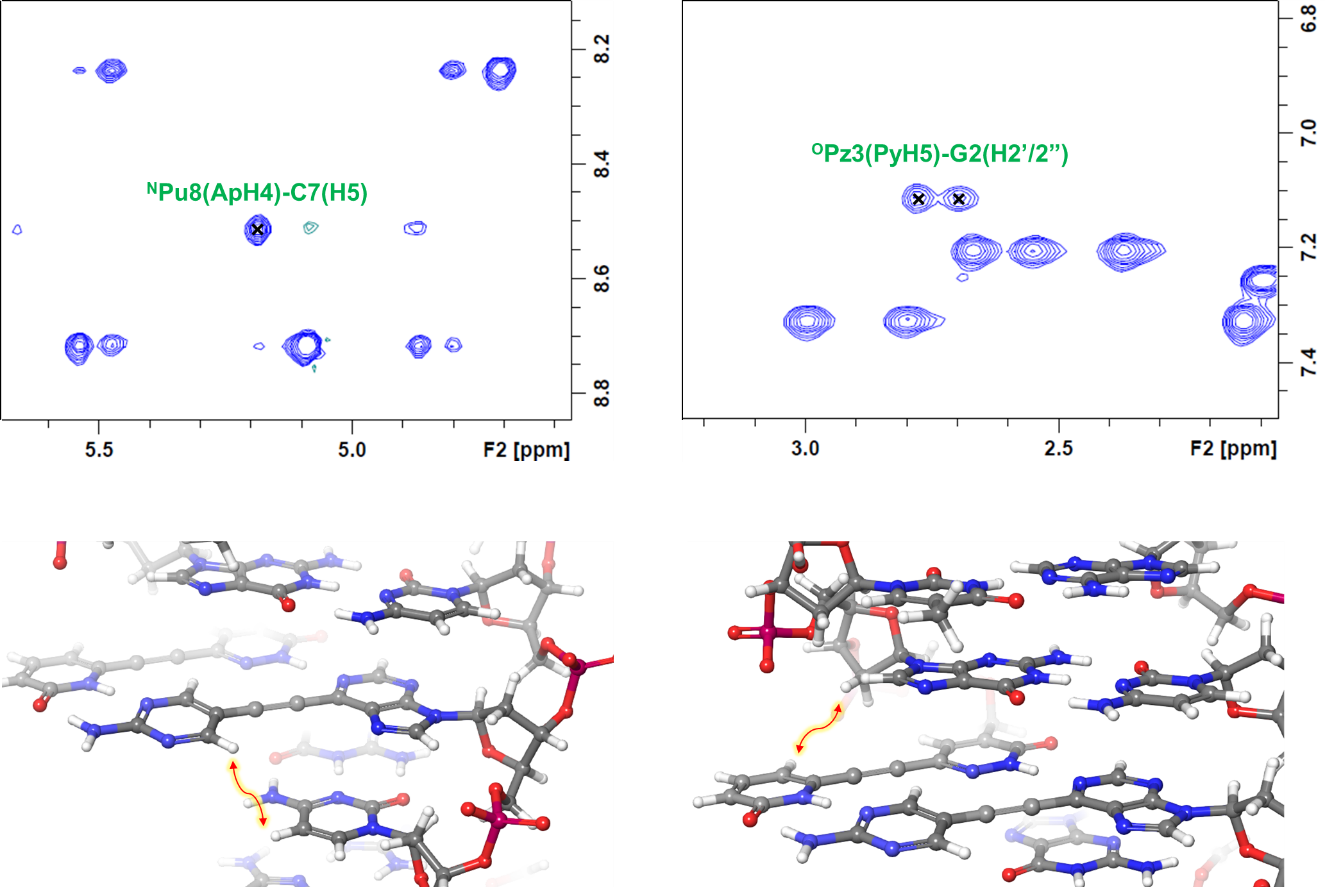


**Figure S14**. Excerpts of NOESY spectra indicating the positioning of pseudo-nucleobases in the major groove. Two NOEs, ^N^Pu8(ApH4)-C7(H5) and ^O^Pz3(PyH5)-G2(H2’/2”), were used for ^1^H chemical shift assignment of ApH4 of 2-aminopyrimidine and PyH5 of 2-pyridone moieties, respectively. The spectrum was measured in D_2_O at 5 °C.


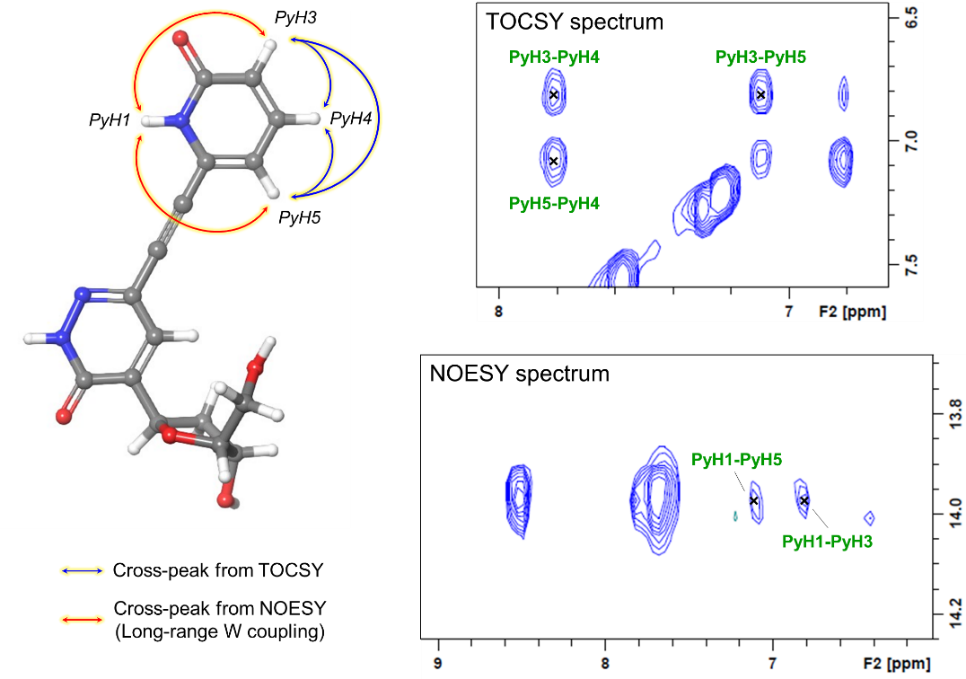


**Figure S15**. Assignment of ^1^H chemical shifts of the 2-pyridone moiety of the ^O^Pz residue. A TOCSY spectrum was used to identify the other two C-H protons (PyH3, PyH4) of the 2-pyridone ring. Furthermore, NOEs exhibited by PyH3/PyH5 toward an imino proton at 13.95 ppm were used for identification of PyH1. The measurements were performed under the following conditions: D_2_O at 5 °C for TOCSY and H_2_O-D_2_O at 5 °C for NOESY.

**9. MD simulations of the duplexes containing the alkynylated purine-pyridazine pairs**


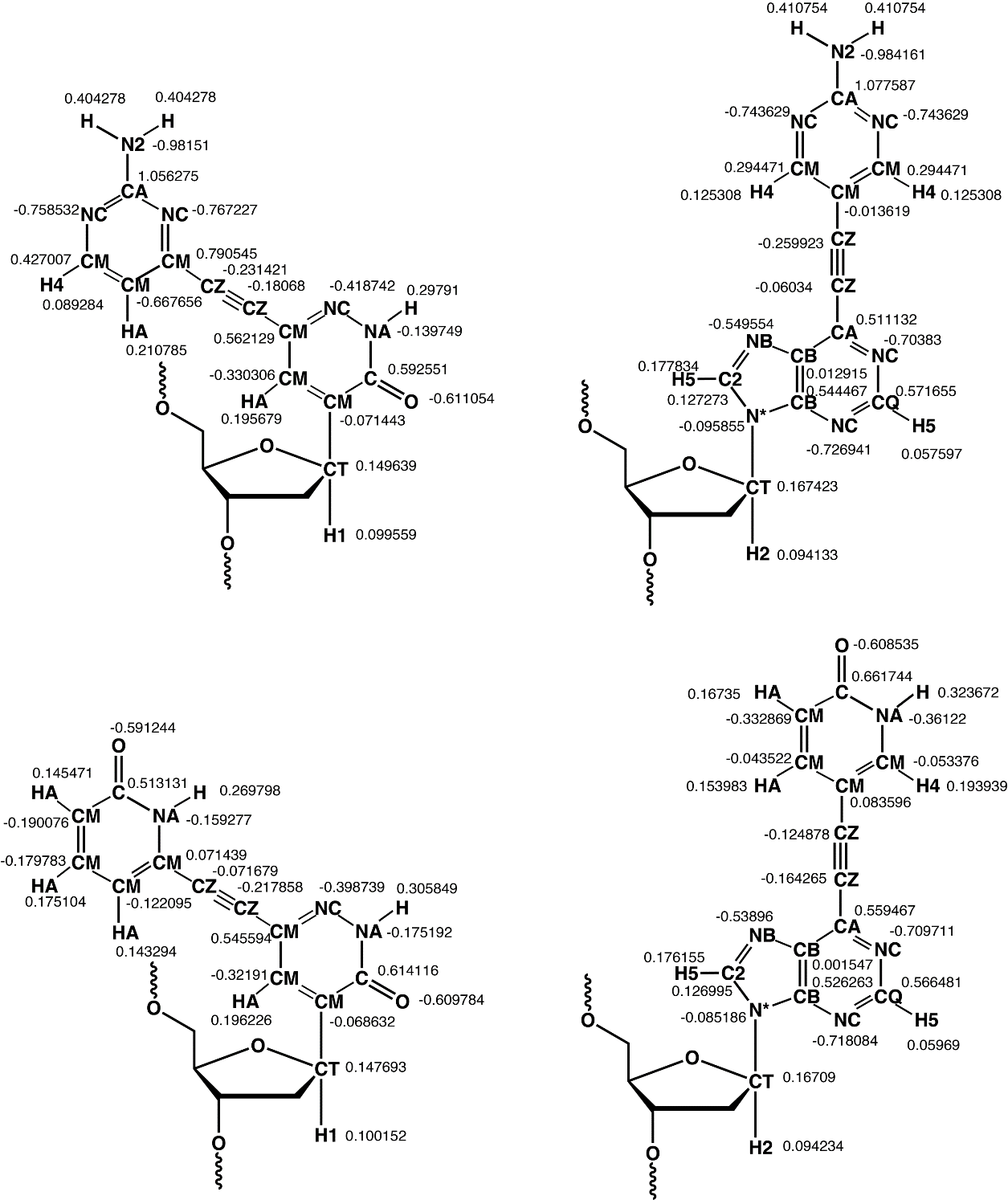


**Figure S16**. Atom types and charges assigned for the alkynylated nucleosides.

**
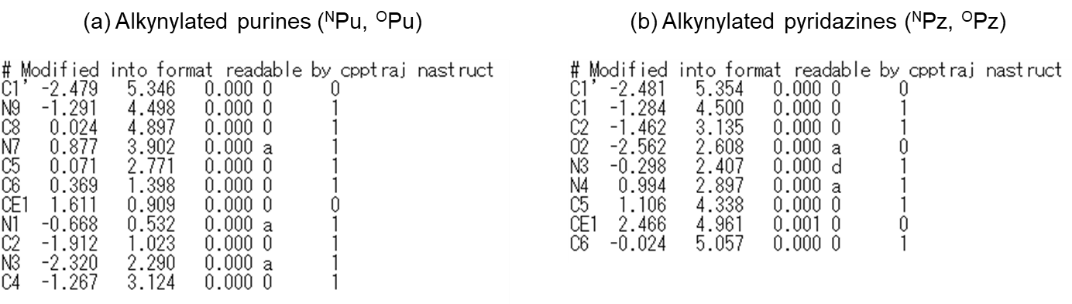
**

**Figure S17**. Reference files used for the calculation of base pair and base step parameters for (a) alkynylated purine and (b) pyridazine nucleosides

**
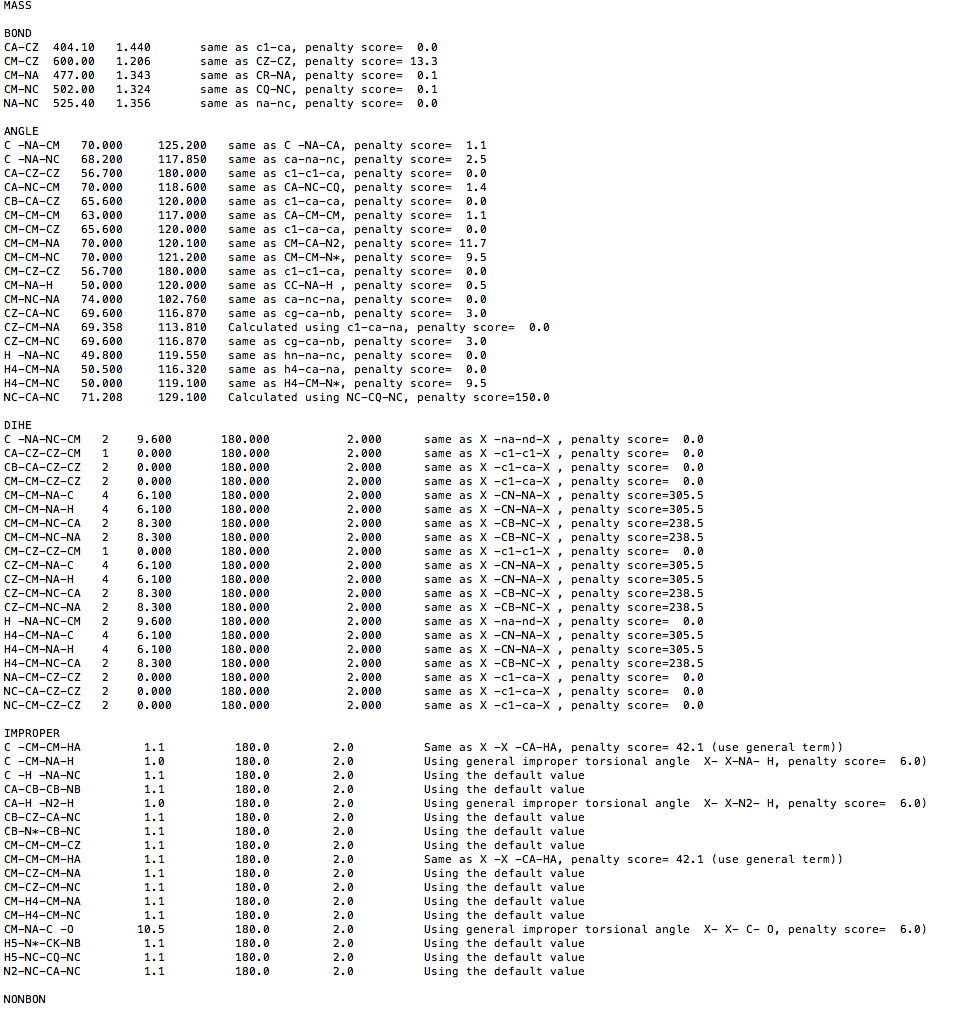
**

**Figure S18**. Additional force fields used in the calculation.


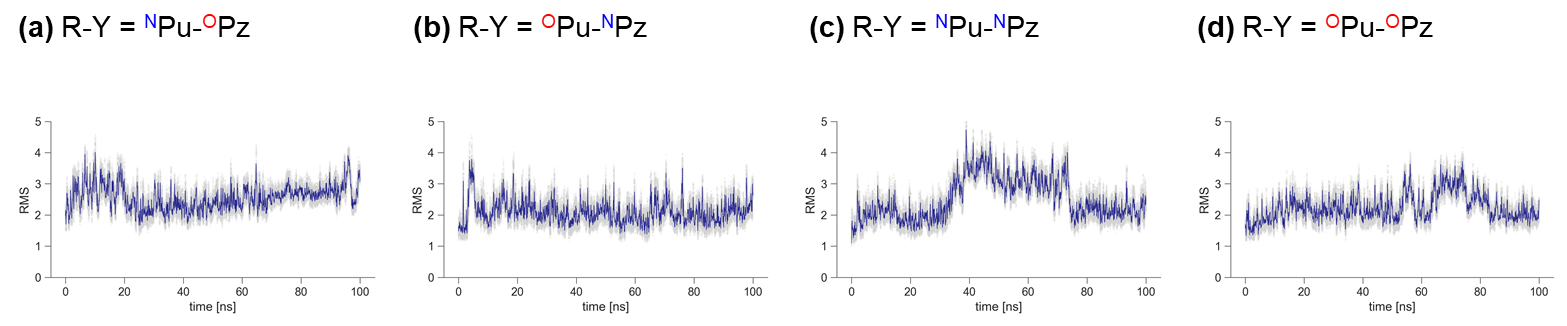


**Figure S19**. Structural deviation of the DNA duplexes incorporating each pair of the alkynylated nucleosides compared to their initial structures during 100 ns of the calculation.


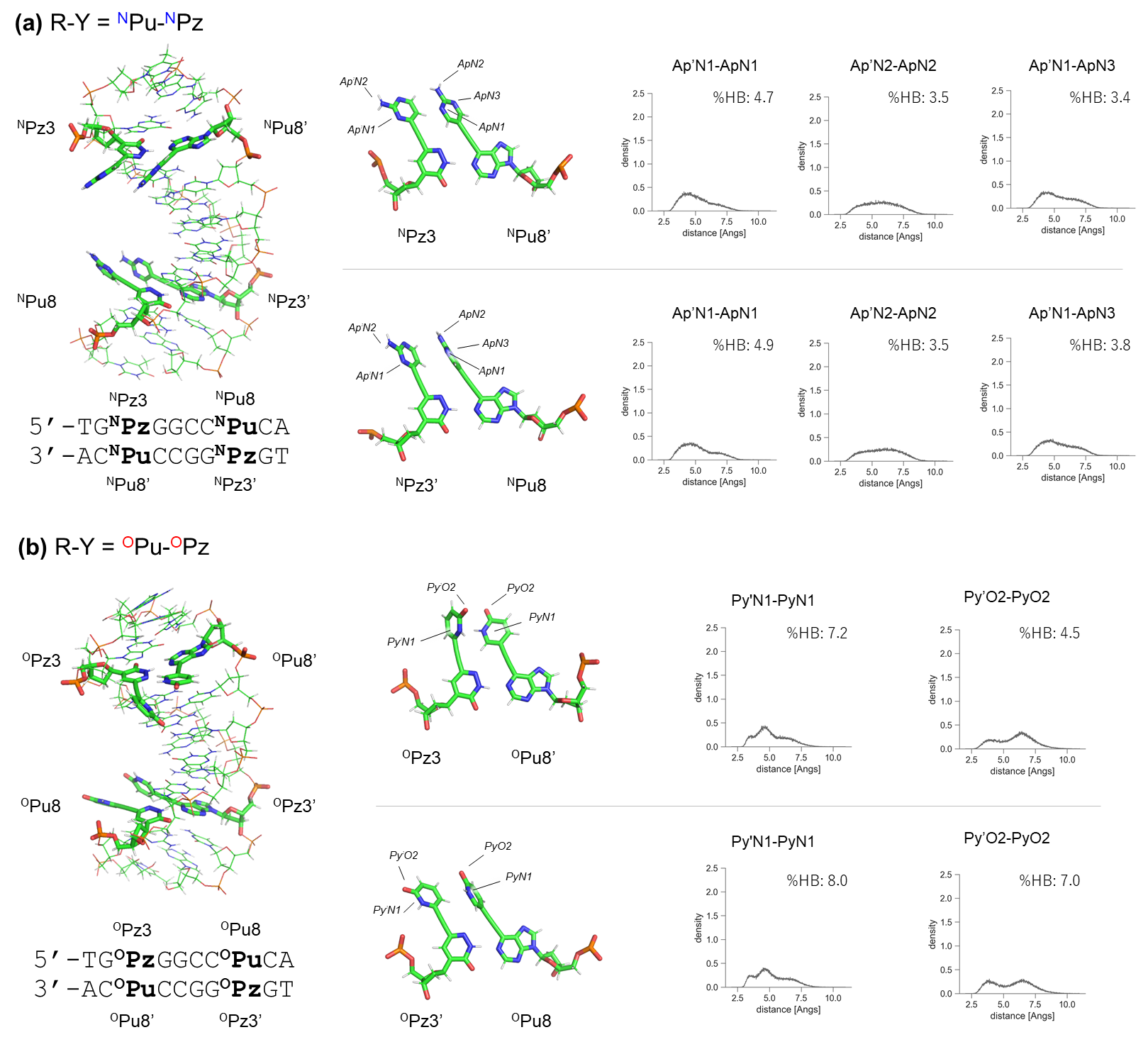


**Figure S20**. Energy minimized structures of the last snapshot from the MD calculations of the DNA duplexes featuring (a) ^N^Pu-^N^Pz and (b) ^O^Pu-^O^Pz. The MD calculation was performed using AMBER force field with TIP3P water model for 100 ns. The distances between the hydrogen-bonding heteroatoms on the pseudo-nucleobases are shown in histograms. %HB denotes the percentage of frames showing less than 3.5 Å between the heteroatoms in 100ns simulation.

**
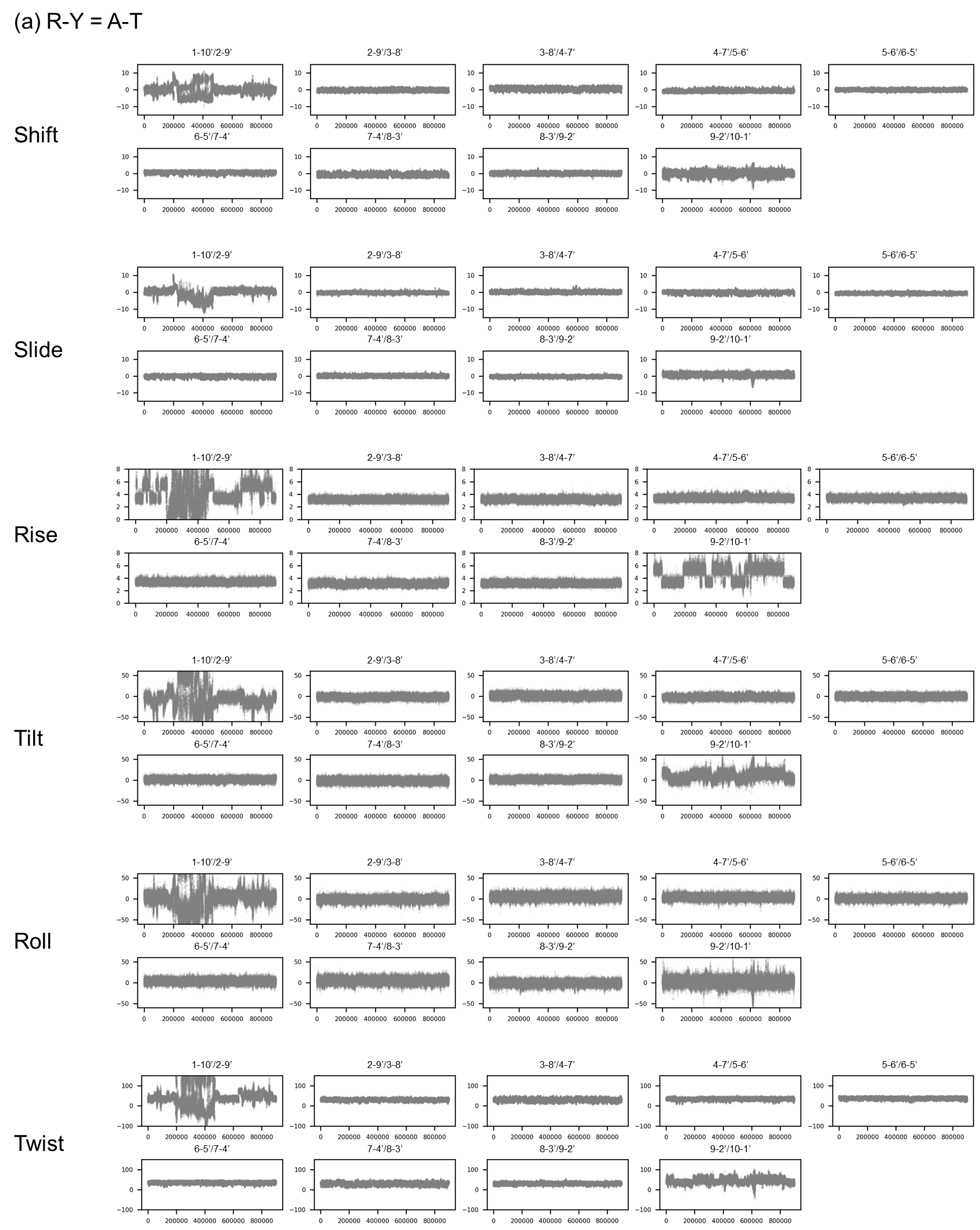
**

**Figure S21**. The simulation results of base pair and base step parameters of the 10-bp DNA duplexes containing (a) A-T, (b) ^N^Pu-^O^Pz, (c) ^O^Pu-^N^Pz, (d) ^N^Pu-^N^Pz, and (e) ^O^Pu-^O^Pz pairs over 100 ns of the calculation.

**
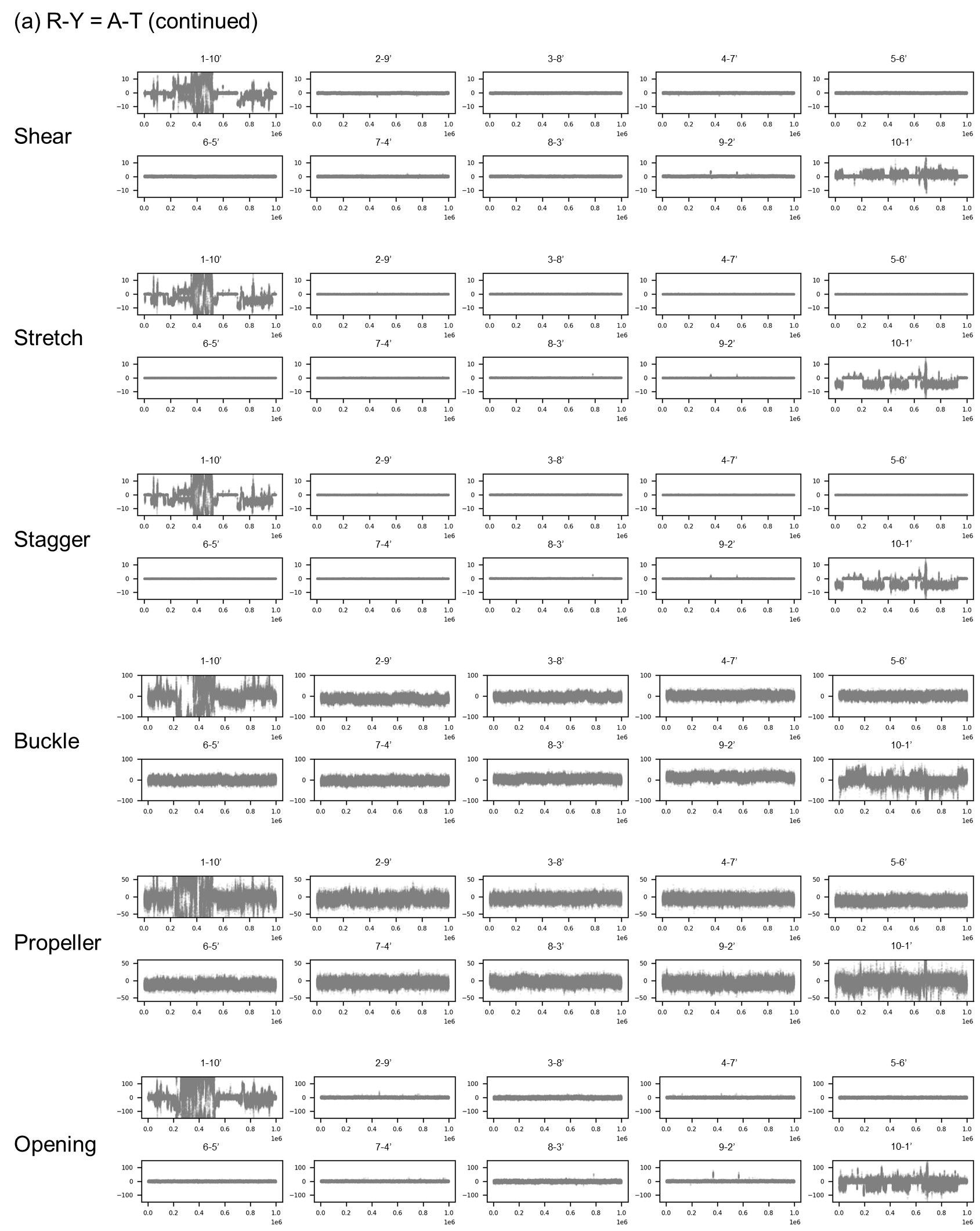
**

**Figure S21 (continued)**. The simulation results of base pair and base step parameters of the 10-bp DNA duplexes containing (a) A-T, (b) ^N^Pu-^O^Pz, (c) ^O^Pu-^N^Pz, (d) ^N^Pu-^N^Pz, and (e) ^O^Pu-^O^Pz pairs over 100 ns of the calculation.

**
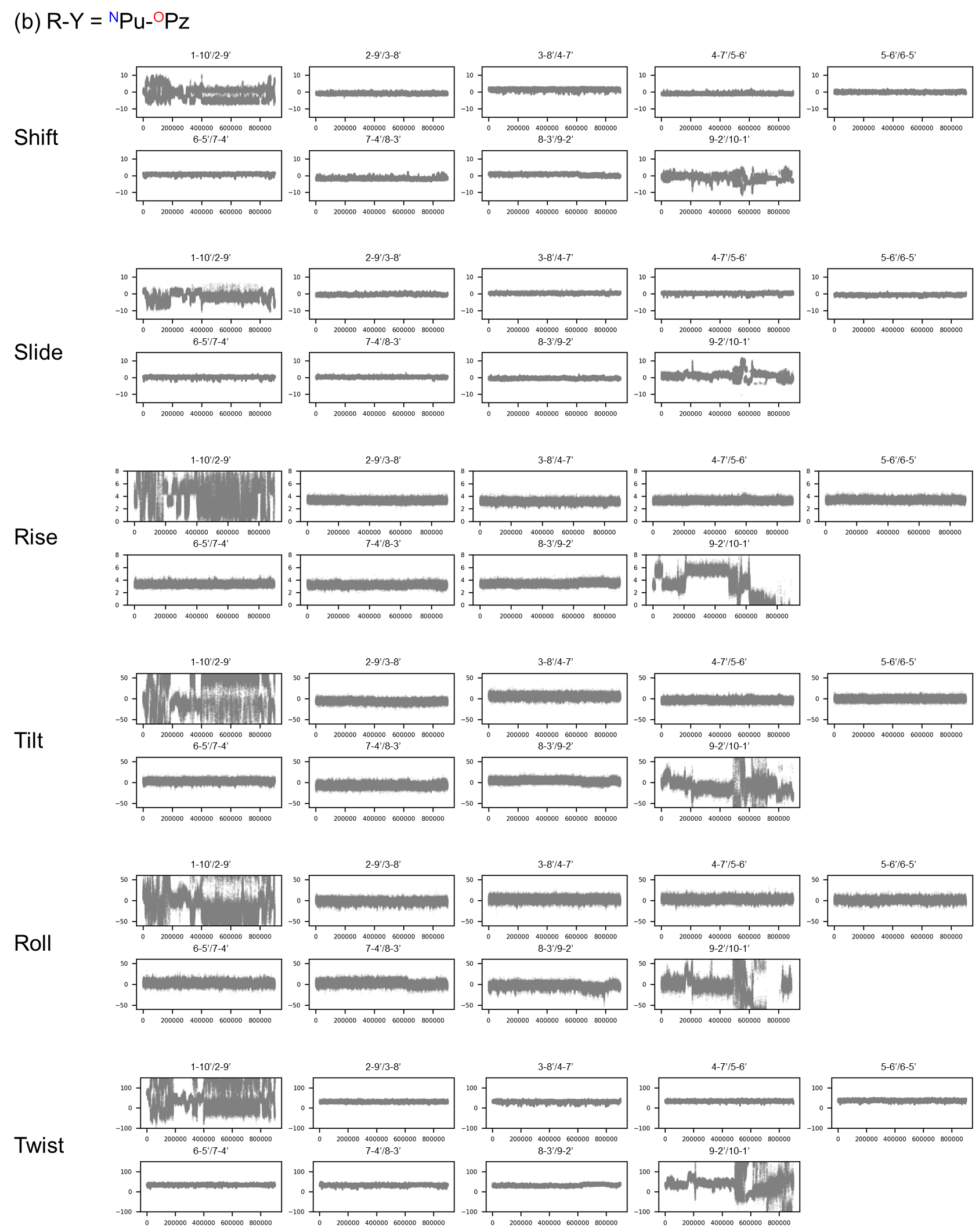
**

**Figure S21 (continued)**. The simulation results of base pair and base step parameters of the 10-bp DNA duplexes containing (a) A-T, (b) ^N^Pu-^O^Pz, (c) ^O^Pu-^N^Pz, (d) ^N^Pu-^N^Pz, and (e) ^O^Pu-^O^Pz pairs over 100 ns of the calculation.

**
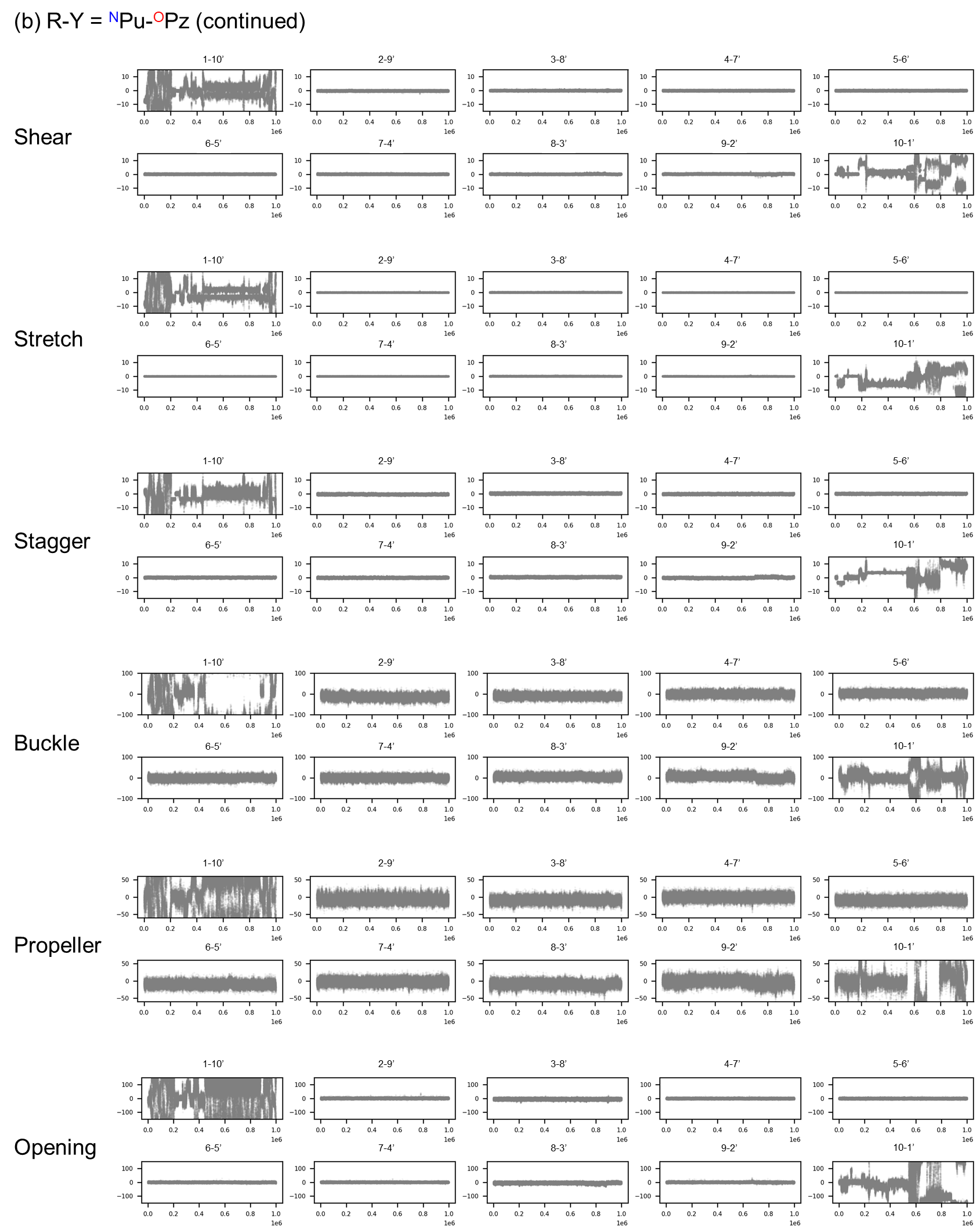
**

**Figure S21 (continued)**. The simulation results of base pair and base step parameters of the 10-bp DNA duplexes containing (a) A-T, (b) ^N^Pu-^O^Pz, (c) ^O^Pu-^N^Pz, (d) ^N^Pu-^N^Pz, and (e) ^O^Pu-^O^Pz pairs over 100 ns of the calculation.

**
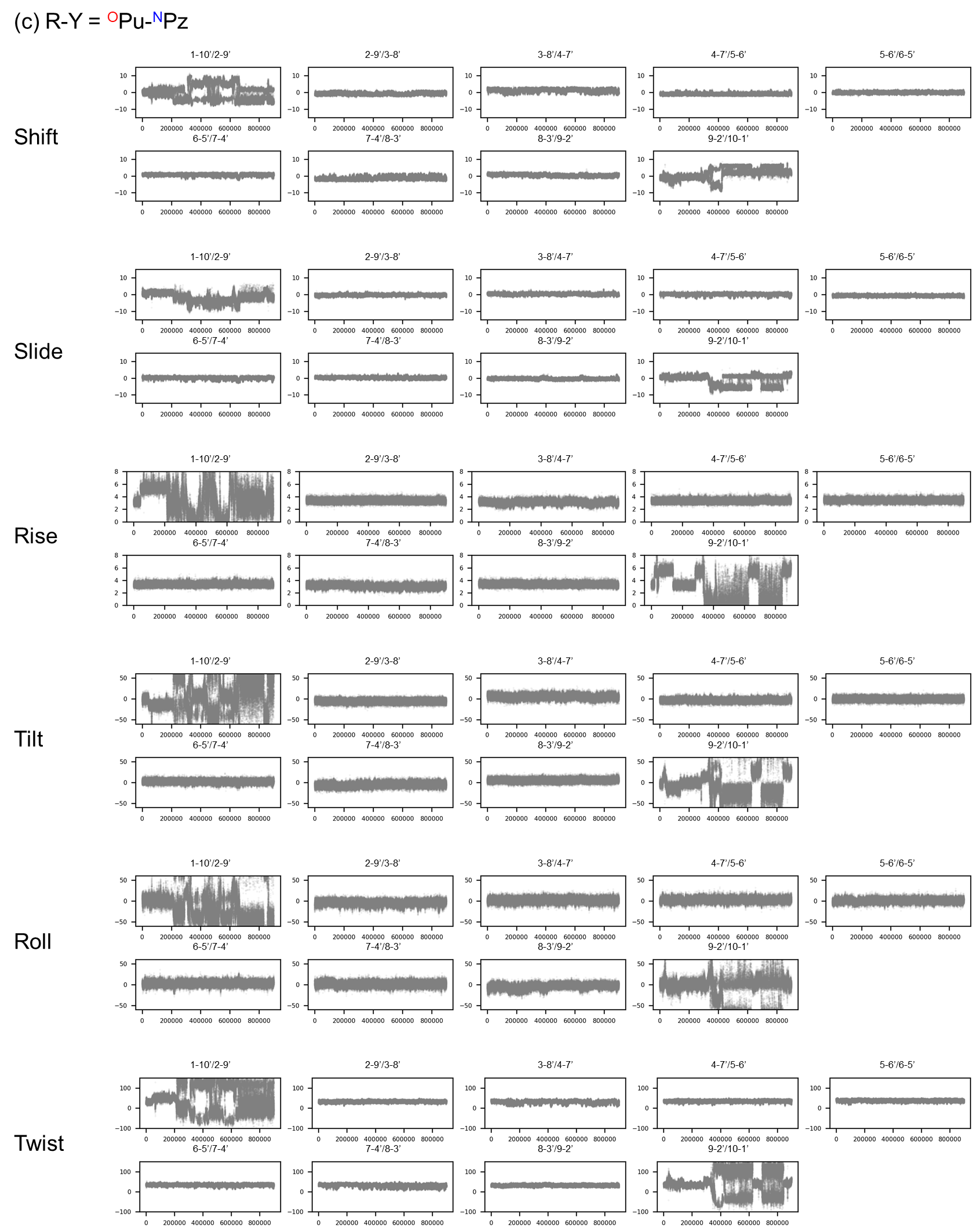
**

**Figure S21 (continued)**. The simulation results of base pair and base step parameters of the 10-bp DNA duplexes containing (a) A-T, (b) ^N^Pu-^O^Pz, (c) ^O^Pu-^N^Pz, (d) ^N^Pu-^N^Pz, and (e) ^O^Pu-^O^Pz pairs over 100 ns of the calculation.

**
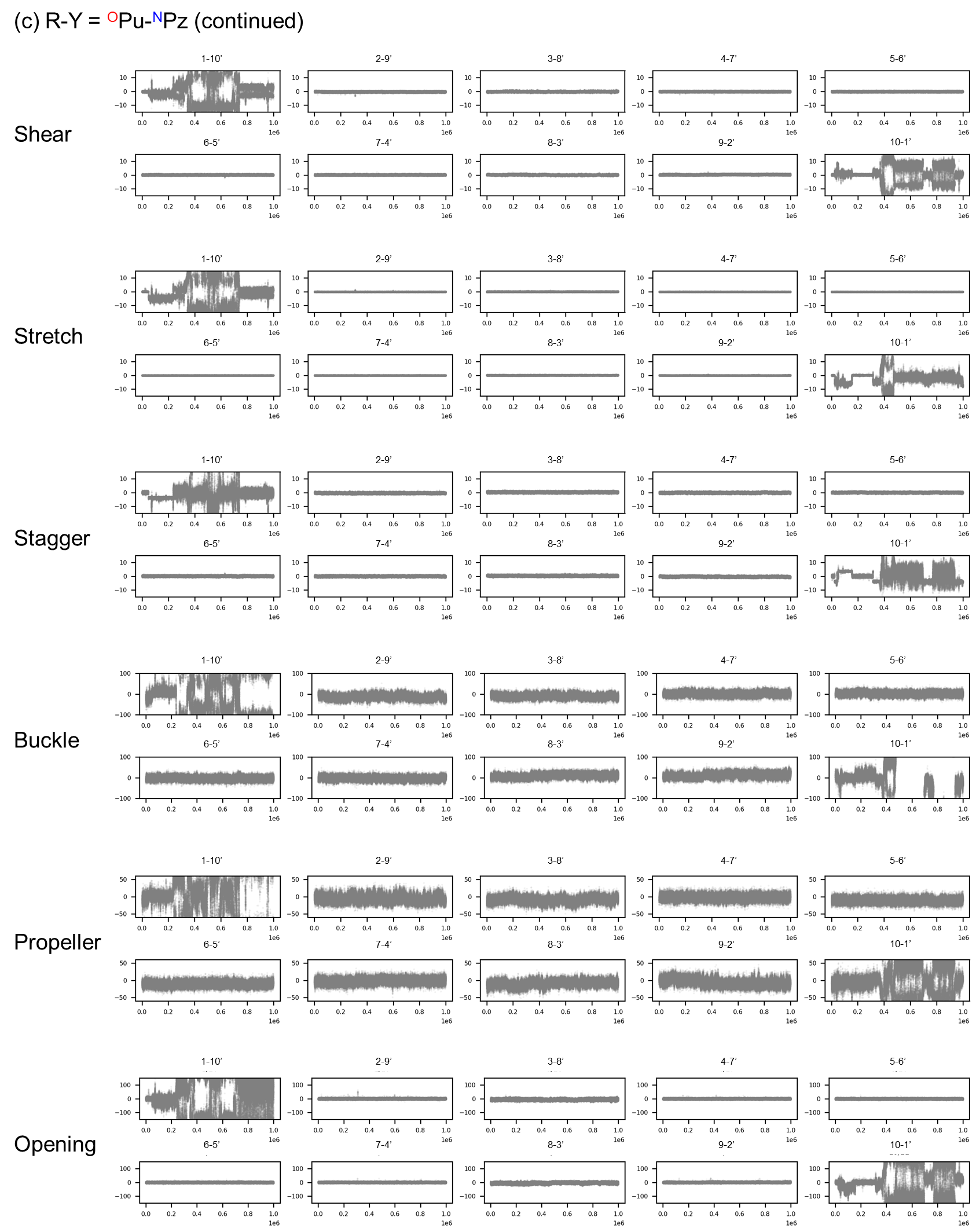
**

**Figure S21 (continued)**. The simulation results of base pair and base step parameters of the 10-bp DNA duplexes containing (a) A-T, (b) ^N^Pu-^O^Pz, (c) ^O^Pu-^N^Pz, (d) ^N^Pu-^N^Pz, and (e) ^O^Pu-^O^Pz pairs over 100 ns of the calculation.

**
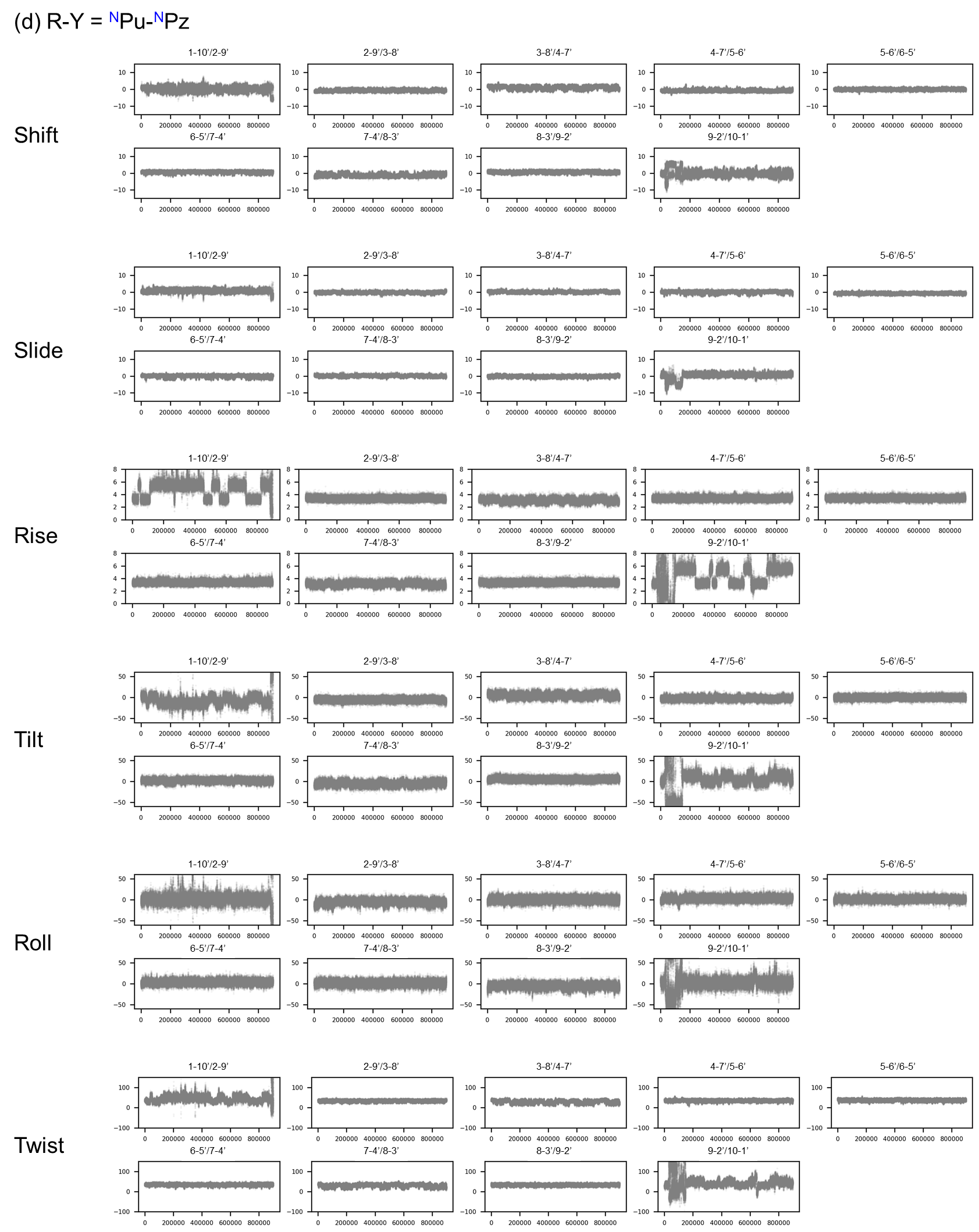
**

**Figure S21 (continued)**. The simulation results of base pair and base step parameters of the 10-bp DNA duplexes containing (a) A-T, (b) ^N^Pu-^O^Pz, (c) ^O^Pu-^N^Pz, (d) ^N^Pu-^N^Pz, and (e) ^O^Pu-^O^Pz pairs over 100 ns of the calculation.

**
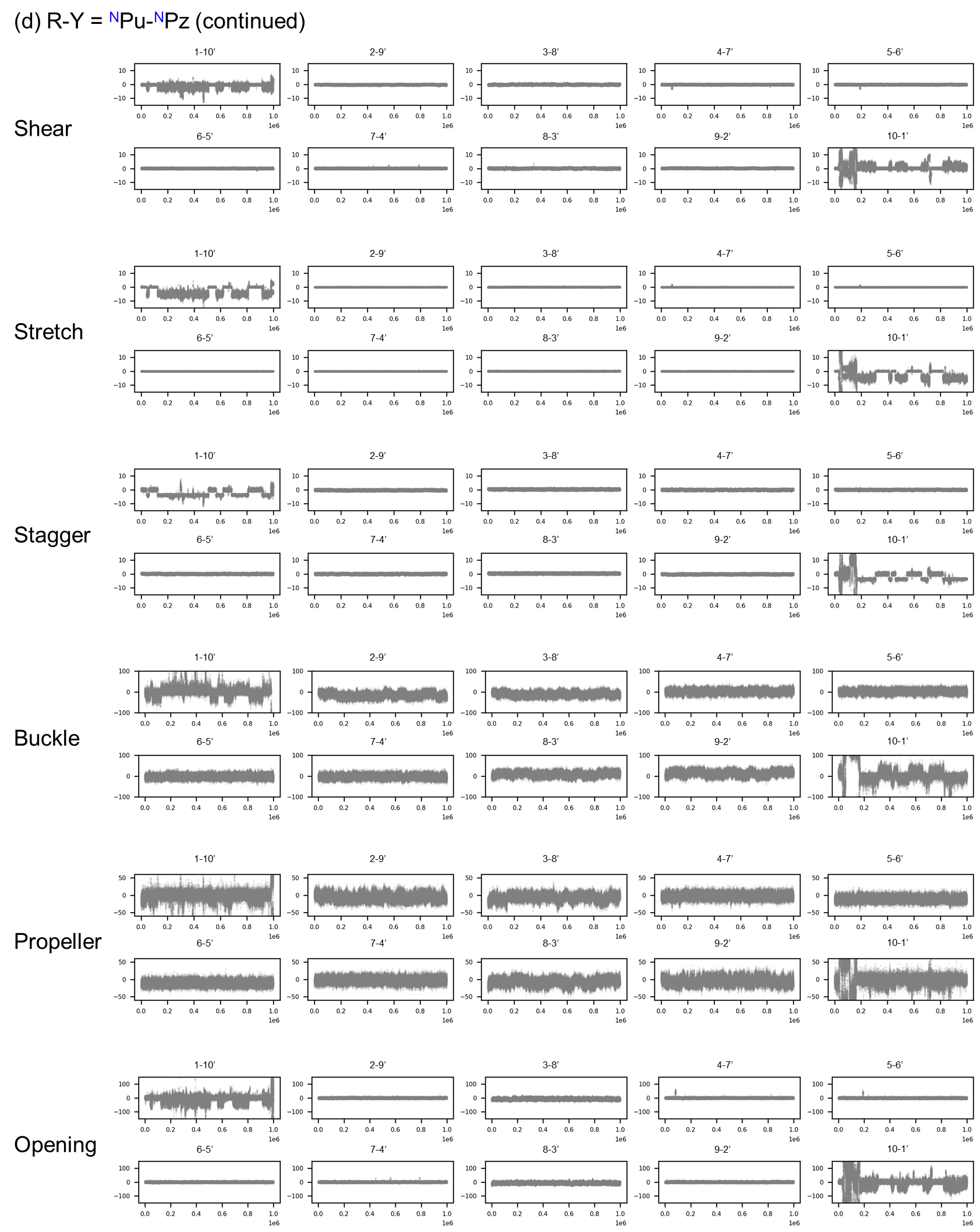
**

**Figure S21 (continued)**. The simulation results of base pair and base step parameters of the 10-bp DNA duplexes containing (a) A-T, (b) ^N^Pu-^O^Pz, (c) ^O^Pu-^N^Pz, (d) ^N^Pu-^N^Pz, and (e) ^O^Pu-^O^Pz pairs over 100 ns of the calculation.

**
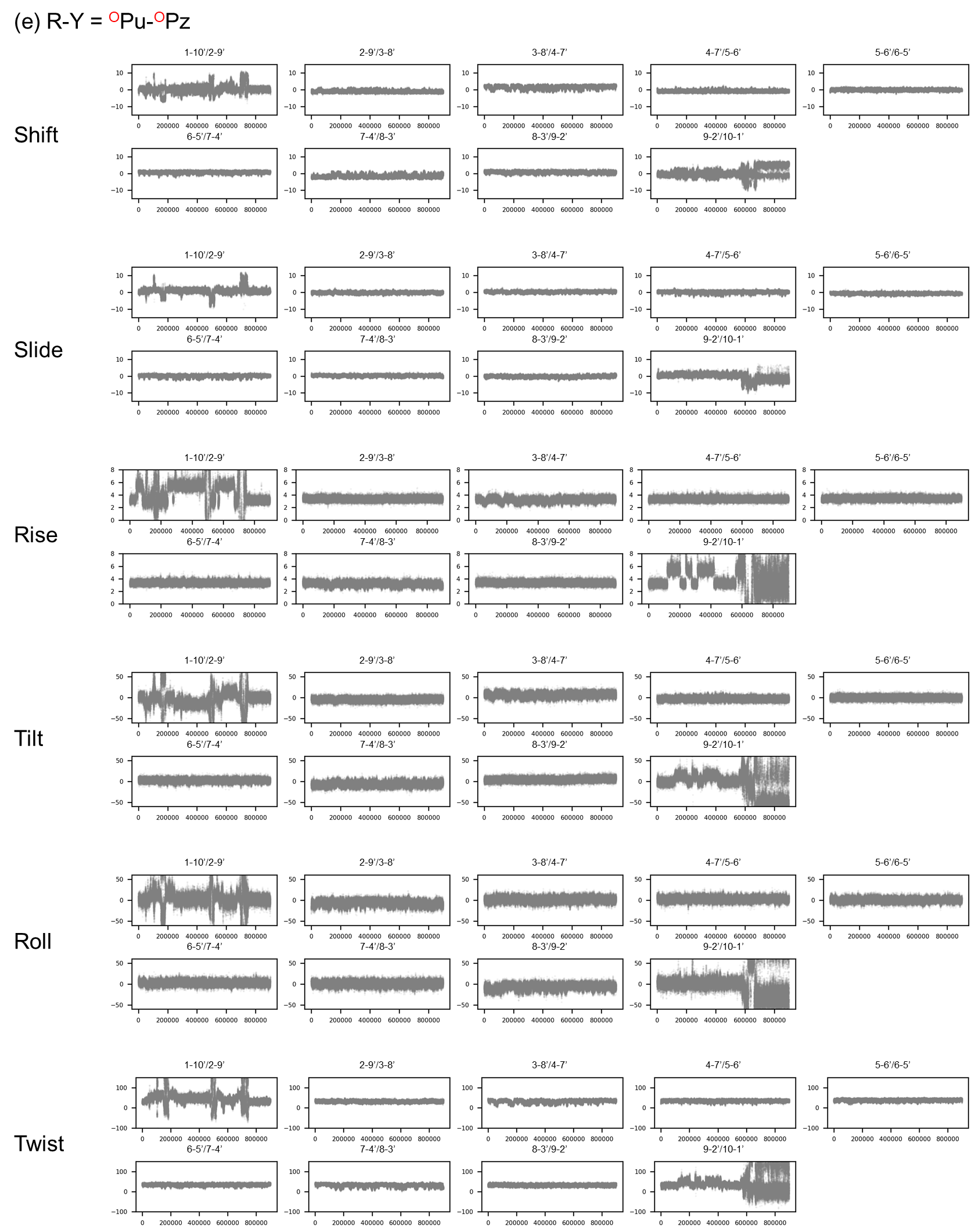
**

**Figure S21 (continued)**. The simulation results of base pair and base step parameters of the 10-bp DNA duplexes containing (a) A-T, (b) ^N^Pu-^O^Pz, (c) ^O^Pu-^N^Pz, (d) ^N^Pu-^N^Pz, and (e) ^O^Pu-^O^Pz pairs over 100 ns of the calculation.

**
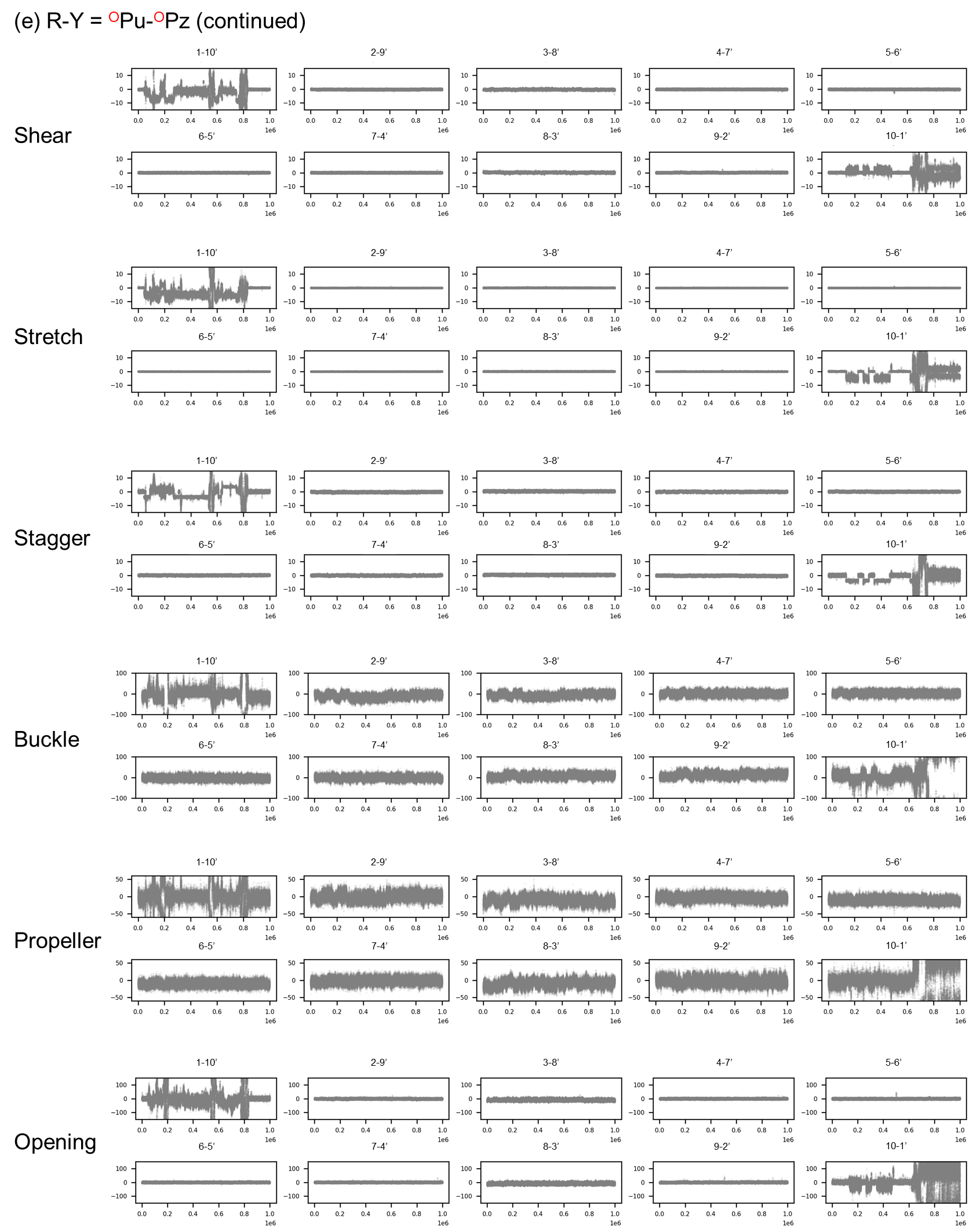
**

**Figure S21 (continued)**. The simulation results of base pair and base step parameters of the 10-bp DNA duplexes containing (a) A-T, (b) ^N^Pu-^O^Pz, (c) ^O^Pu-^N^Pz, (d) ^N^Pu-^N^Pz, and (e) ^O^Pu-^O^Pz pairs over 100 ns of the calculation.

**Table S4**. Summary of mean values of the base pair and base step parameters.

**Table S4 (continued)**. Summary of mean values of the base pair and base step parameters.

**
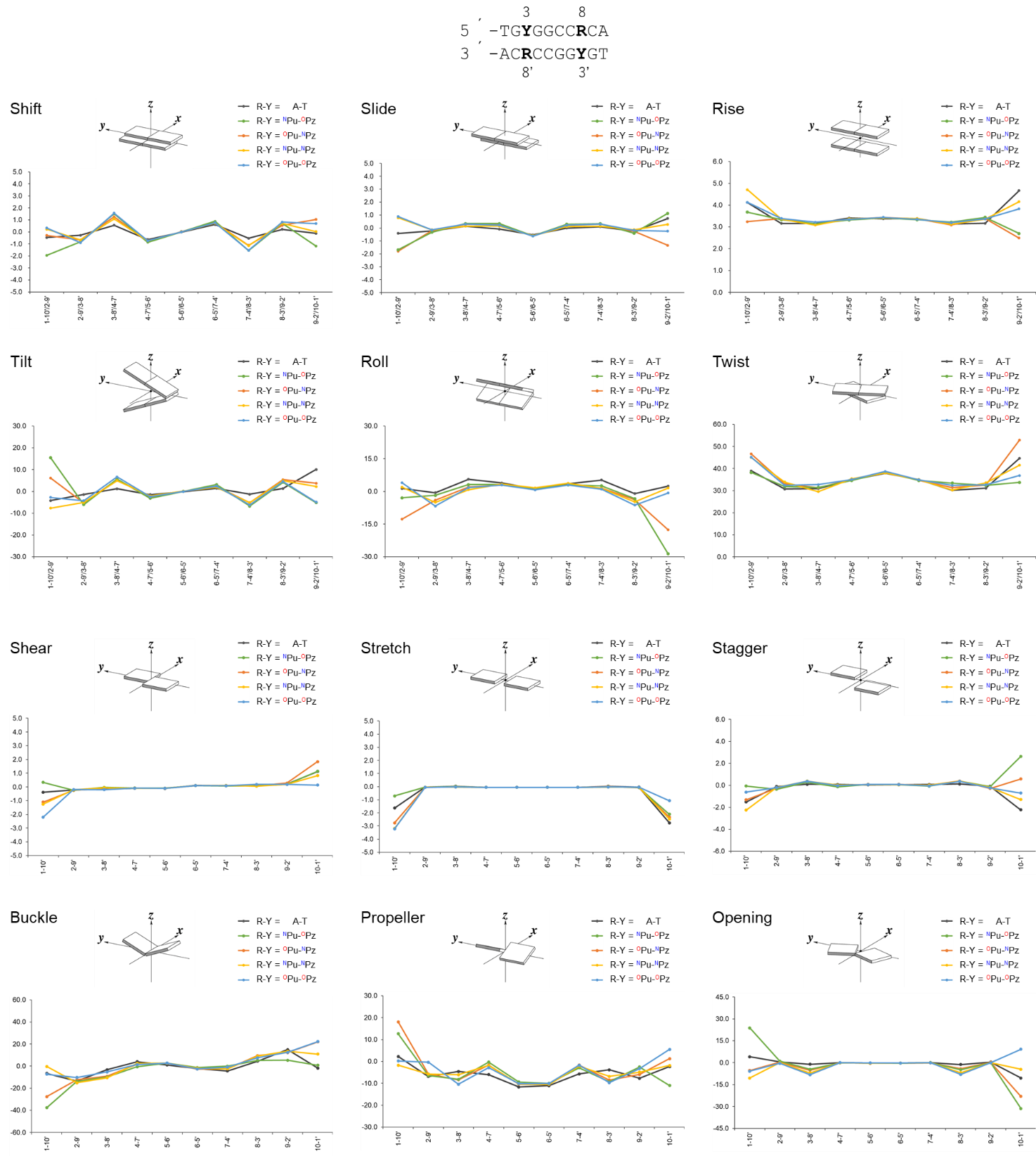
**

**Figure 22**. Comparison of all base pair and base step parameters obtained from the modified and non-modified DNA duplexes. The schematic diagrams of base-pair parameter were adopted from the previous report.^4^ A slight difference was observed for the shift values, which could be explained by the altered stacking geometry of the alkynylated purine-pyridazine pairs with the adjacent bases. Also, the unnatural pairs provided slightly varied tilt as well as propeller and opening values in comparison with those of A-T pair. This may be ascribed to the rotation around the alkyne spacer between the pseudo-nucleobases and purine/pyridazine core structure; thus, tilting of the pairing pseudo-nucleobases could potentially affect the tilting angle of the purine-pyridazine part and hence the opening, which further influence the propeller angle of the adjacent base pairs.

**10. UV melting analyses of the duplexes containing the alkynylated purines and pyridazines at multiple position**


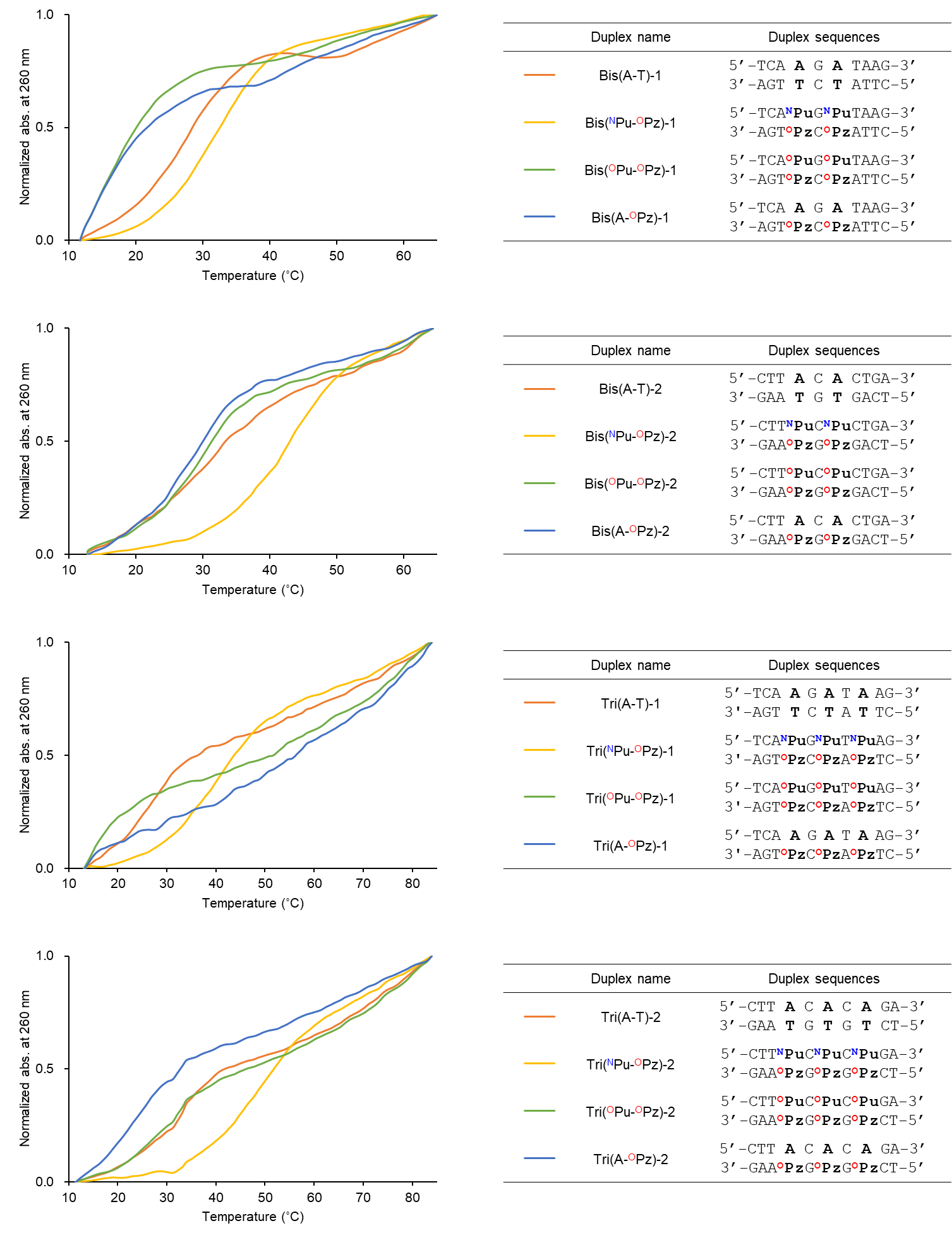


**Figure S23**. UV melting curves of the DNA duplexes containing non-contiguous ^N^Pu-^O^Pz, ^O^Pu-^O^Pz and A-^O^Pz pairs.

**11. CD spectra of the duplexes containing multiple alkynylated purine-pyridazine pairs**


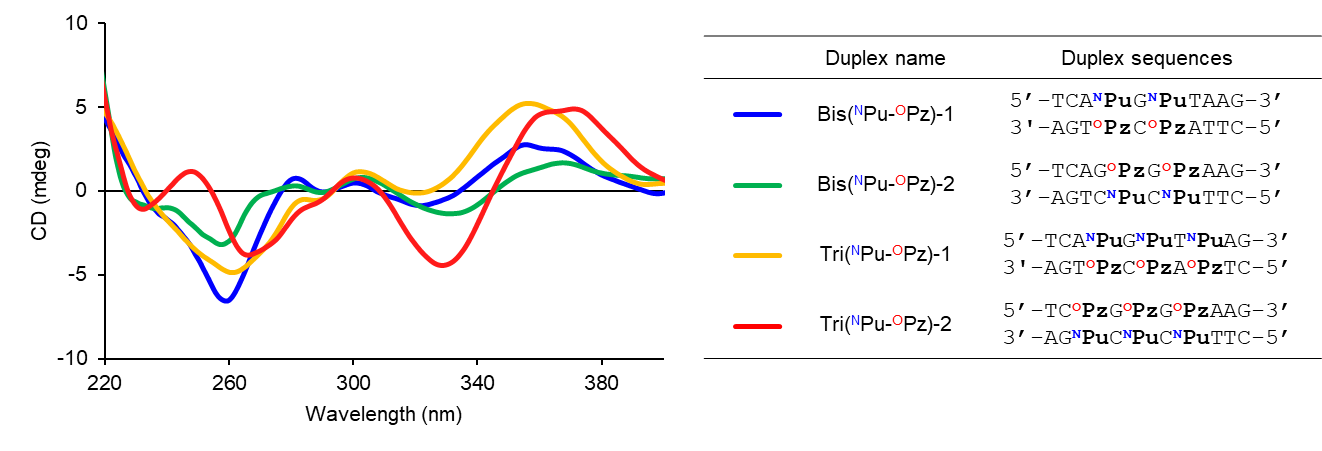


**Figure S24**. CD spectra of the DNA duplexes featuring multiple ^N^Pu-^O^Pz pairs. Conditions: 2 μM of each ODN, 150 mM NaCl, 10 mM sodium phosphate buffer, pH 7.0 at 20 ˚C.

**12. UV melting analyses of the duplexes containing the alkynylated purines and pyridazines at consecutive position**

**
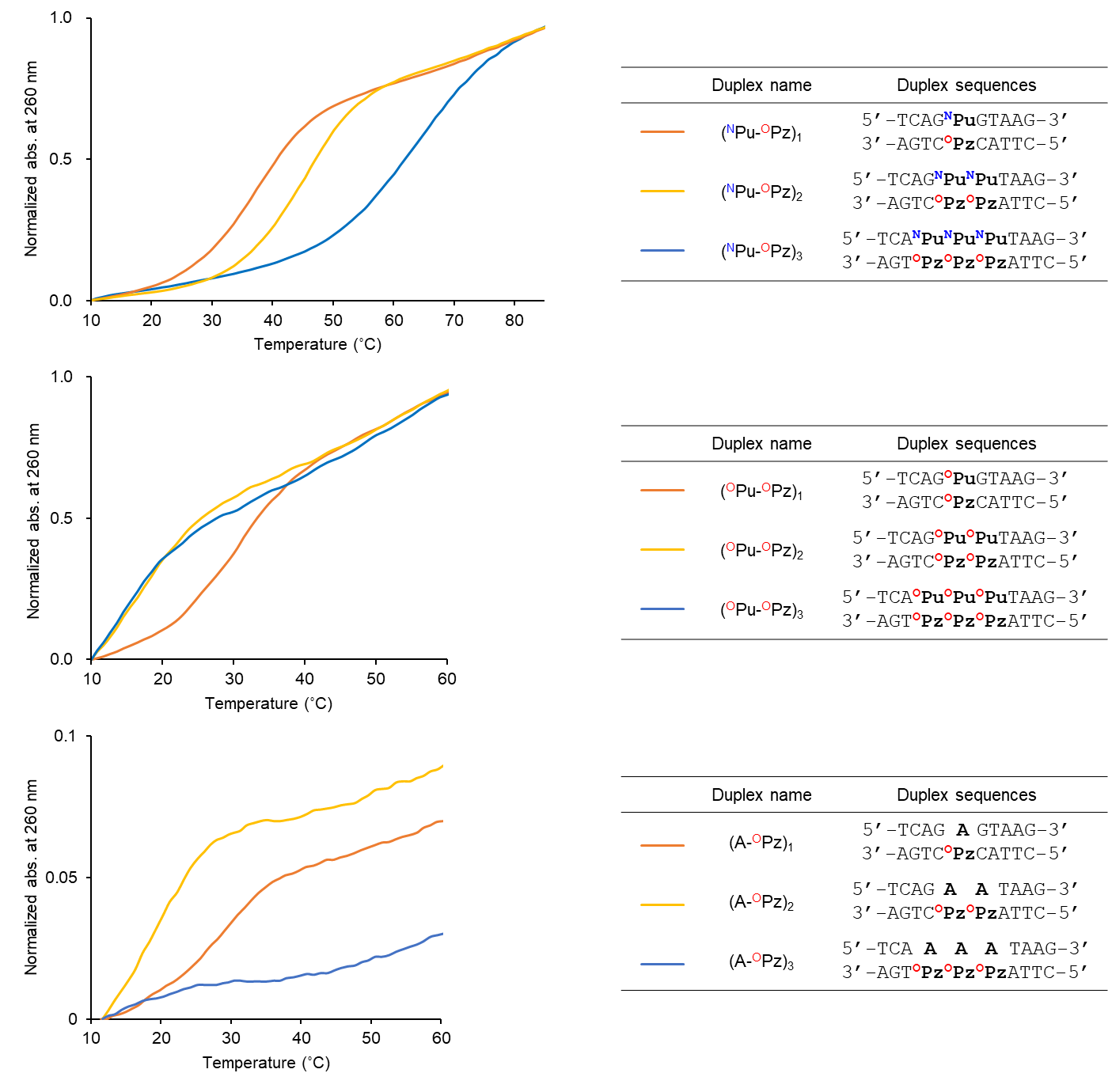
**

**Figure S25**. UV melting curves of the DNA duplexes containing consecutive ^N^Pu-^O^Pz, ^O^Pu-^O^Pz and A-^O^Pz pairs.


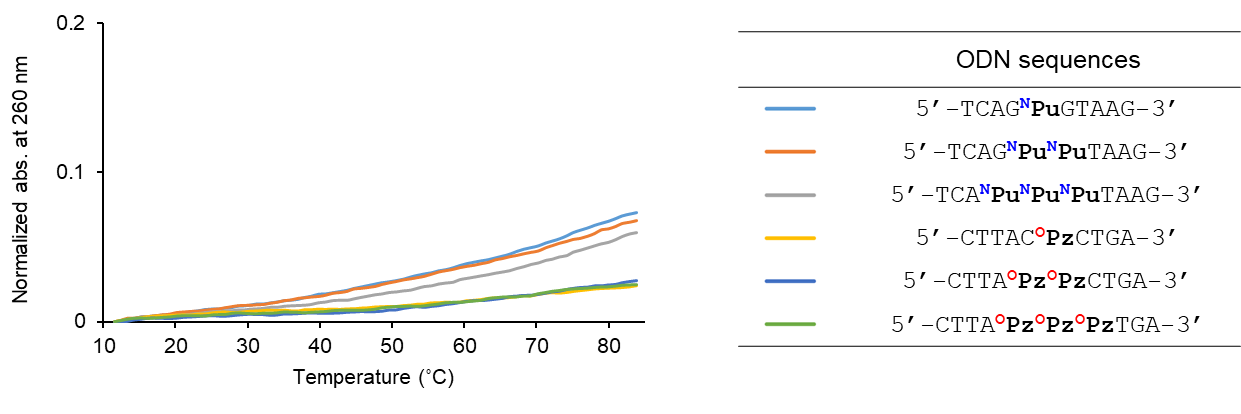


**Figure S26**. Temperature-dependent UV absorbance of the ODNs containing consecutive ^N^Pu and ^O^Pz.


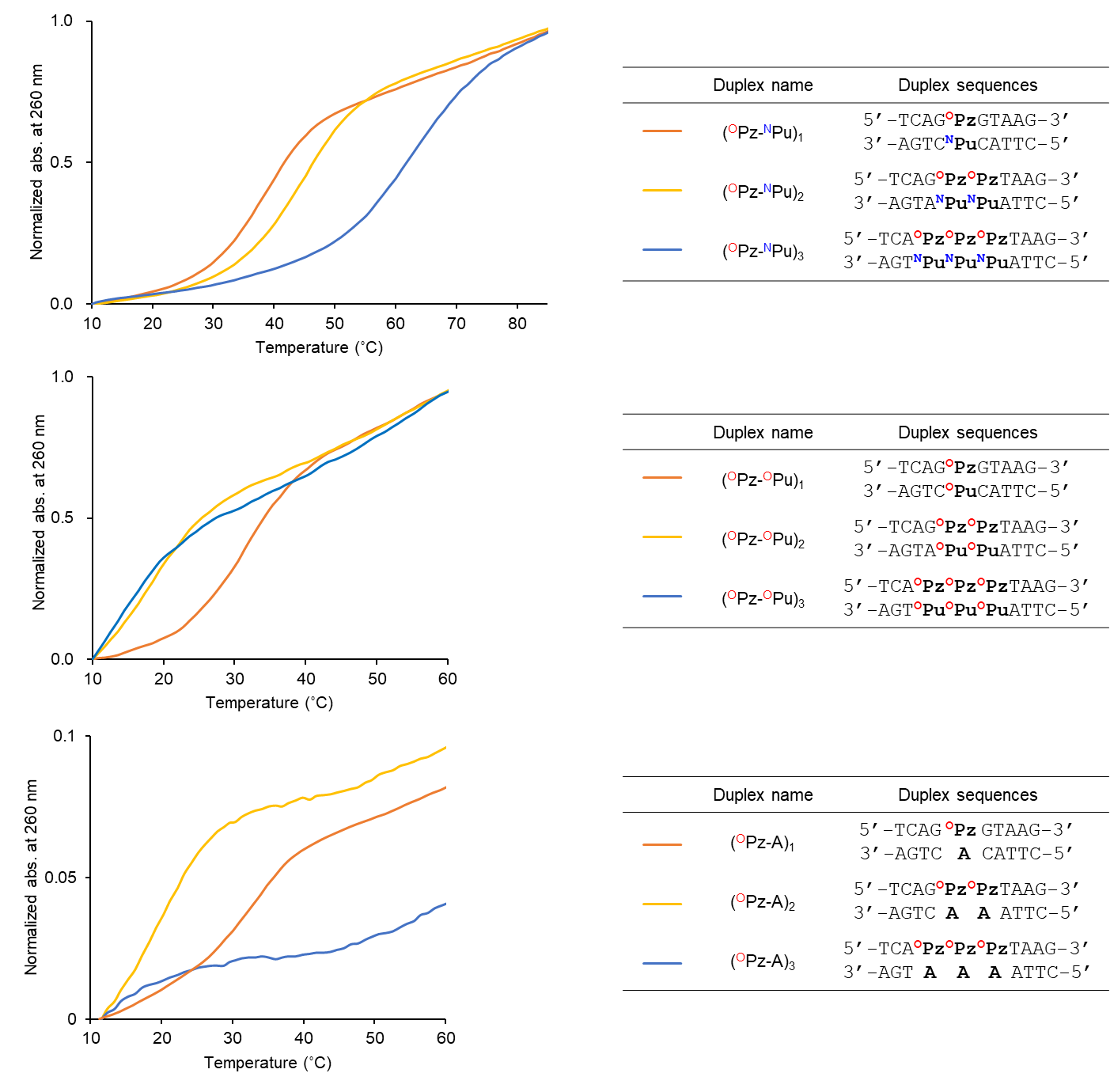


**Figure S27**. UV melting curves of the DNA duplexes containing consecutive ^O^Pz- ^N^Pu, ^O^Pz- ^O^Pu and ^O^Pz- A pairs.


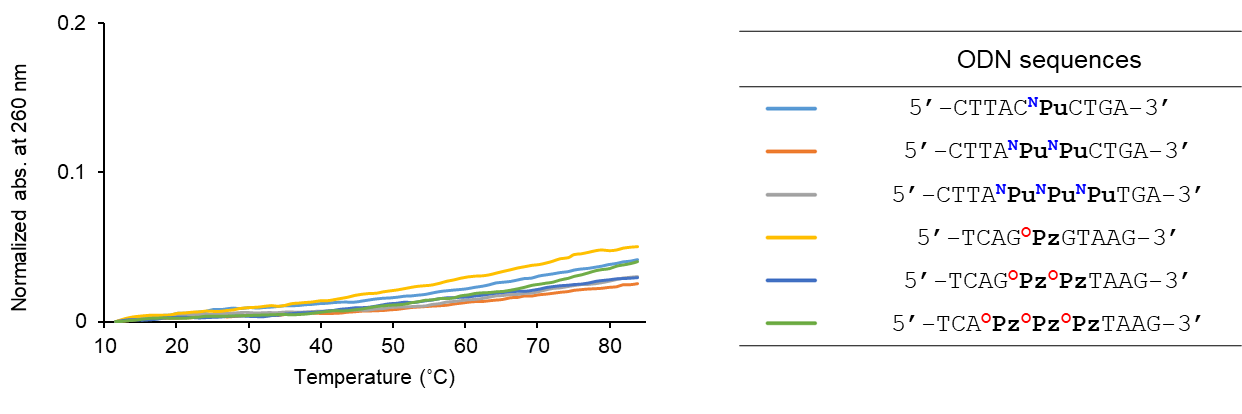


**Figure S28**. Temperature-dependent UV absorbance of the ODNs containing consecutive ^N^Pu and ^O^Pz.

**Table S5.** *T*_m_ values of the DNA duplexes featuring consecutive alkynylated purine and pyridazine nucleosides.


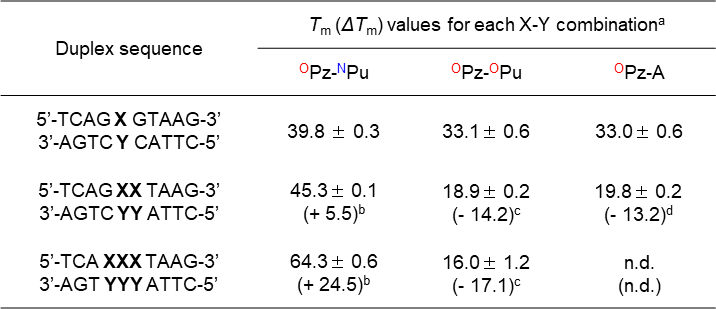


^a^Conditions: 2 μM of each ODN, 150 mM NaCl, 10 mM sodium phosphate buffer, pH 7.0. Each *T*_m_ value is average of three measurements. n.d.: not determined. ^b^To the duplex with single ^O^Pz- ^N^Pu pair. ^c^To the duplex with single ^O^Pz- ^O^Pu pair. ^d^To the duplex with single ^O^Pz-A pair.

**13. Thermodynamic analyses of the duplexes containing consecutive alkynylated purine-pyridazine pair**


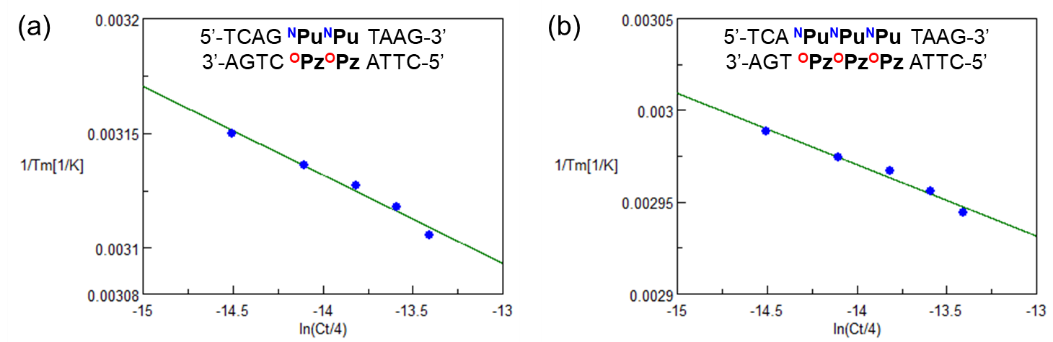


**Figure S29**. Representative Van’t Hoff plots obtained from the UV melting experiments of the DNA duplexes featuring (a) two and (b) three consecutive ^N^Pu-^O^Pz pairs. Conditions: 1-3 μM of each ODN, 150 mM NaCl, 10 mM sodium phosphate buffer.

**Table S6.** Thermodynamic parameters for the DNA duplexes containing consecutive ^N^Pu-^O^Pz pairs.^a^


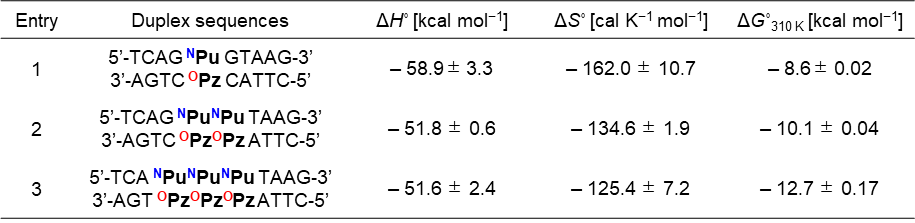


^a^Each thermodynamic parameter was determined from the average of three measurements.

**14. CD spectra of the duplexes containing consecutive alkynylated purine-pyridazine pairs**


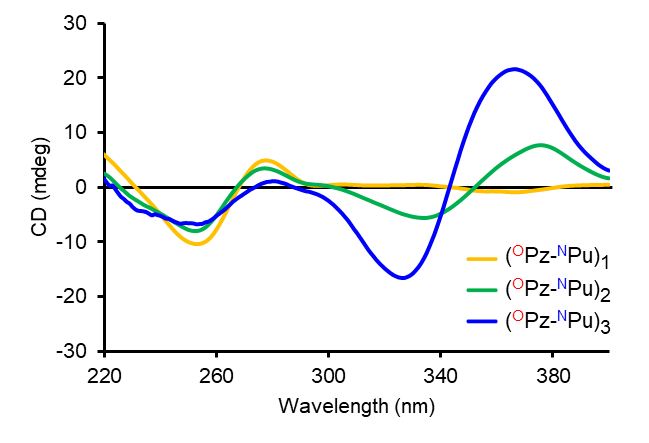


**Figure S30**. CD spectra of the DNA duplexes featuring consecutive ^O^Pz- ^N^Pu pairs. Conditions: 2 μM of each ODN, 150 mM NaCl, 10 mM sodium phosphate buffer, pH 7.0 at 20 ˚C.

**15. NMR spectra of the synthesized compounds**

**Compound S1**

^1^H NMR (CDCl_3_, 400 MHz)


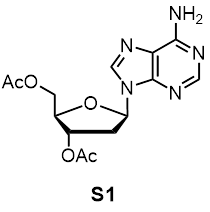

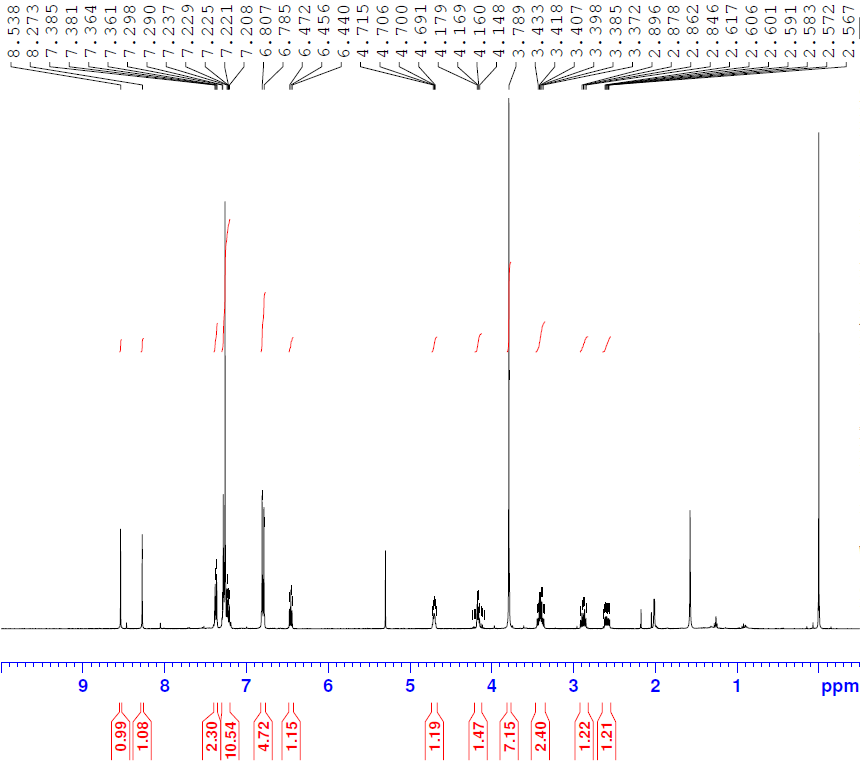


^13^C NMR (CDCl_3_, 151 MHz)


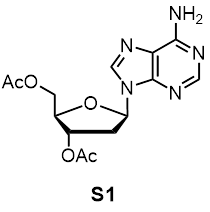

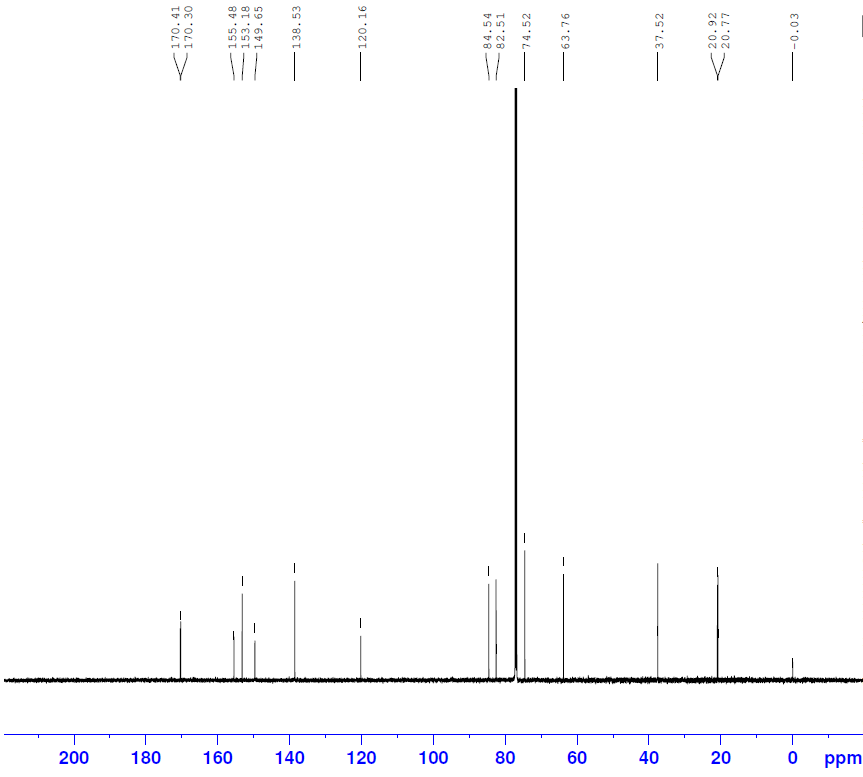


**Compound S2**

^1^H NMR (CDCl_3_, 400 MHz)


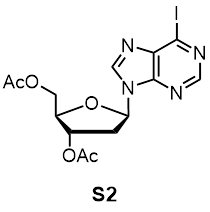

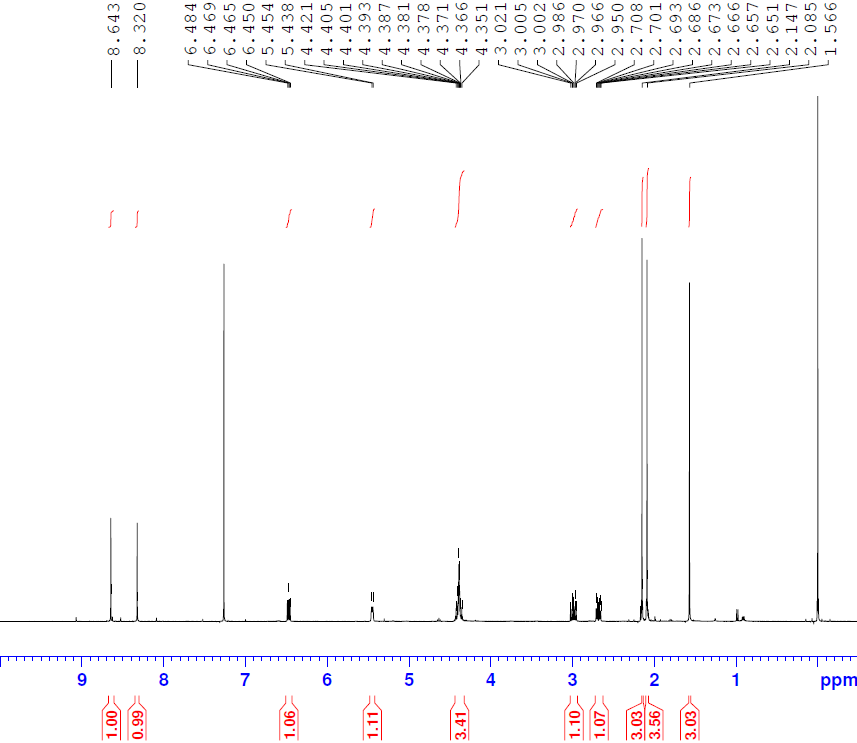


^13^C NMR (CDCl_3_, 151 MHz)


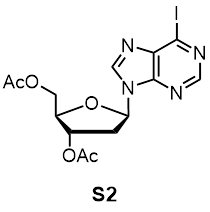

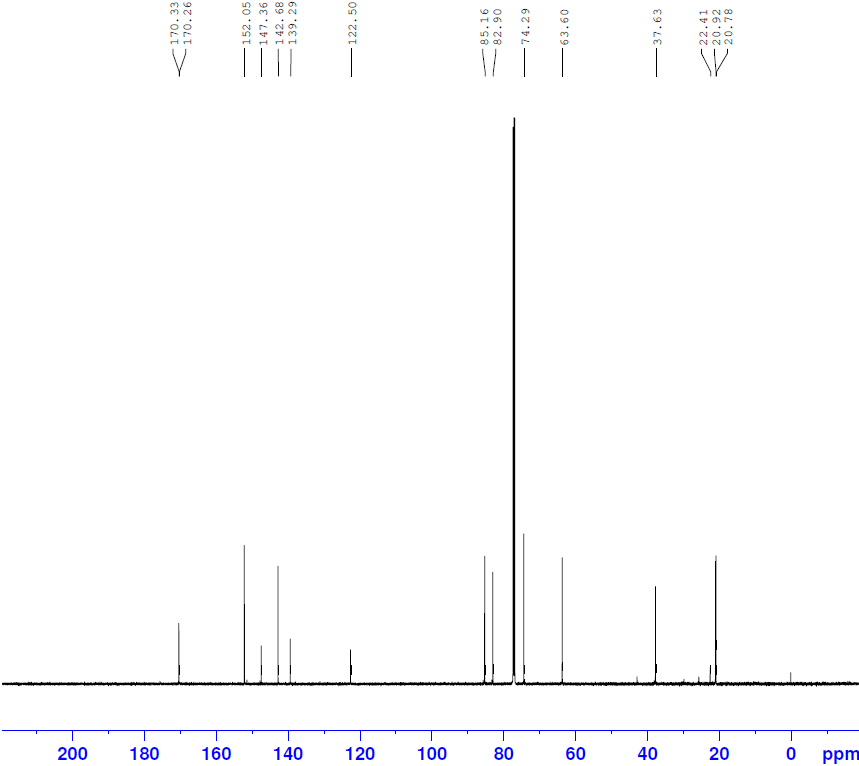


**Compound S3**

^1^H NMR (DMSO-*d*_6_, 400 MHz)


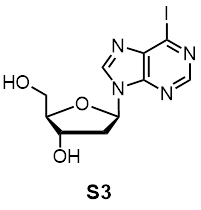

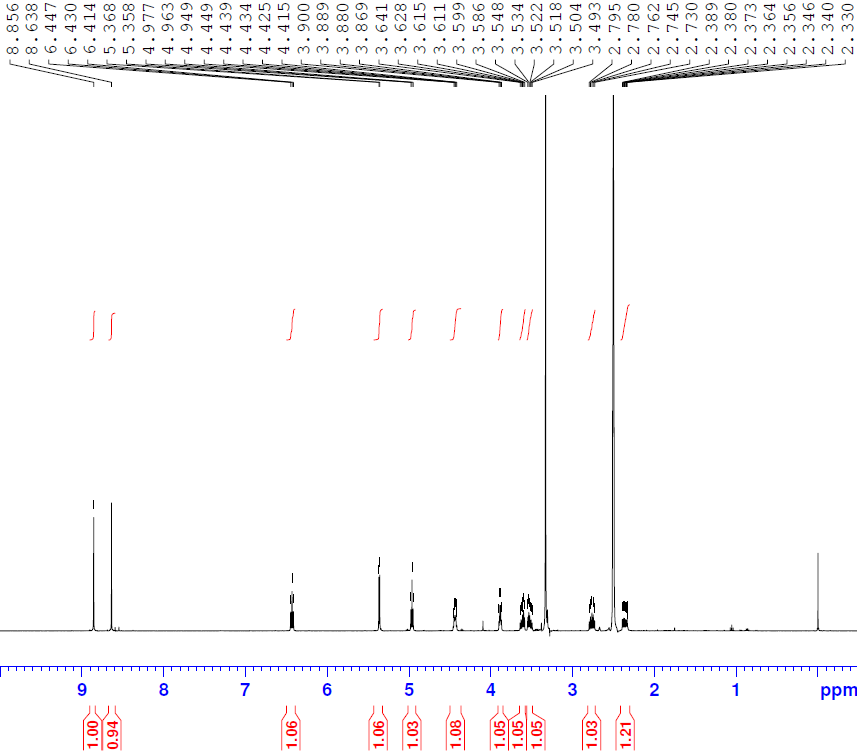


^13^C NMR (DMSO-*d*_6_, 151 MHz)


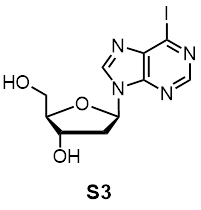

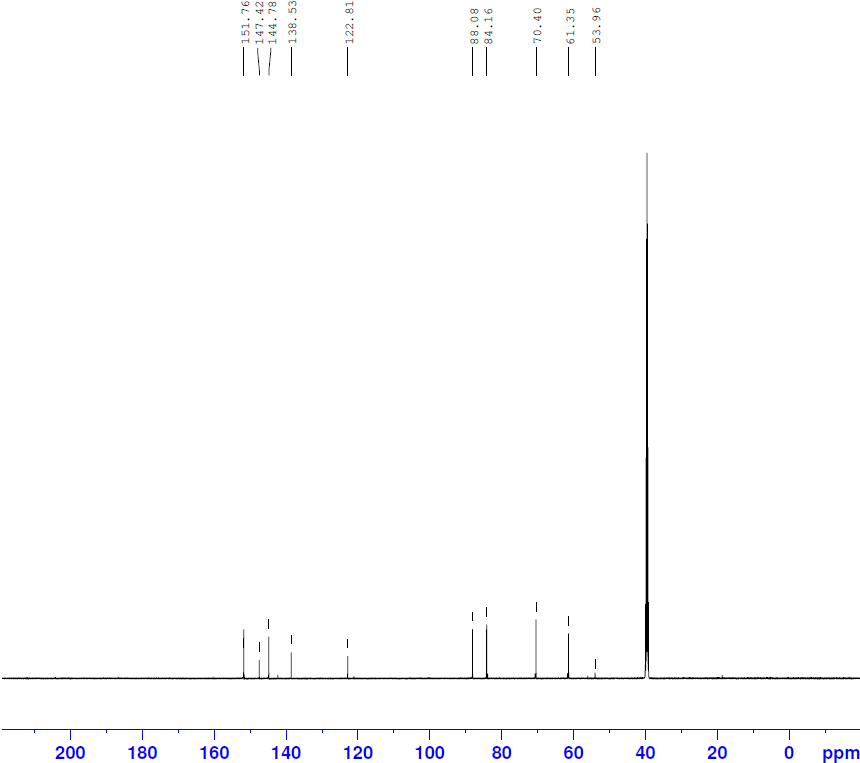


**Compound 1**

^1^H NMR (CDCl_3_, 400 MHz)


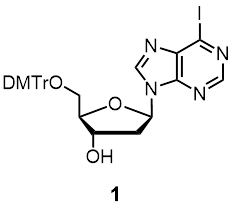

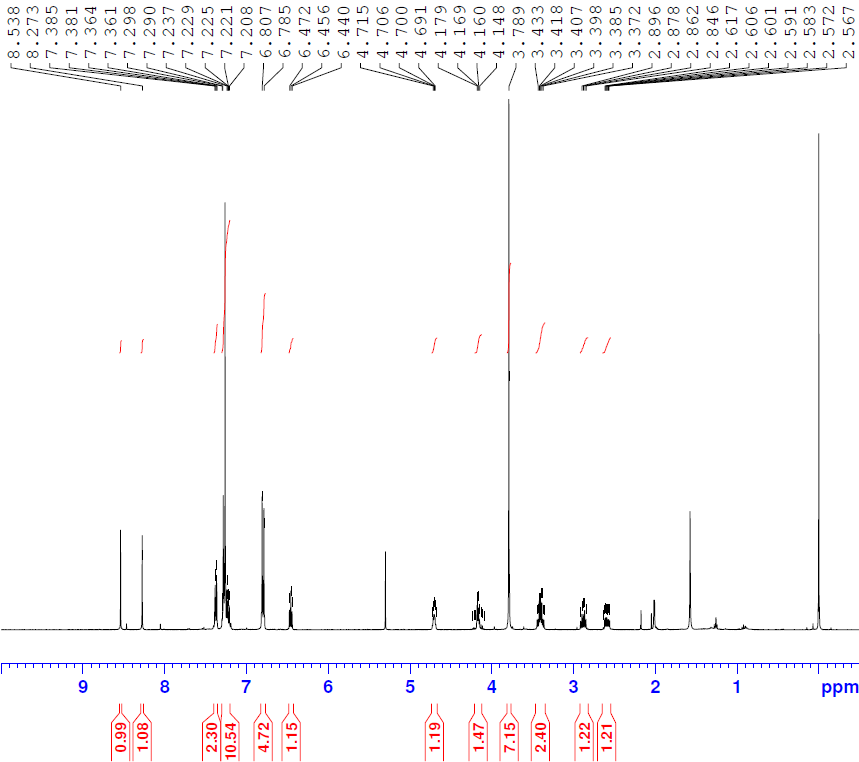


^13^C NMR (CDCl_3_, 151 MHz)


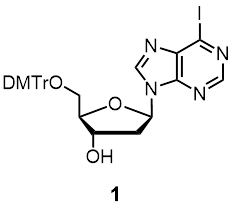

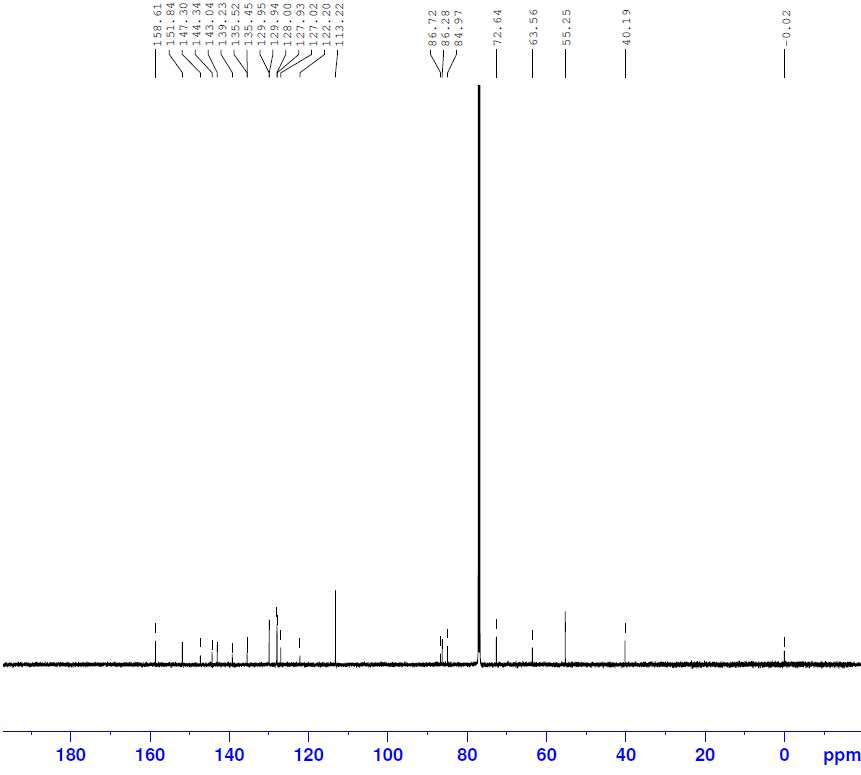


**Compound S4**

^1^H NMR (CDCl_3_, 400 MHz)


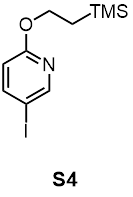

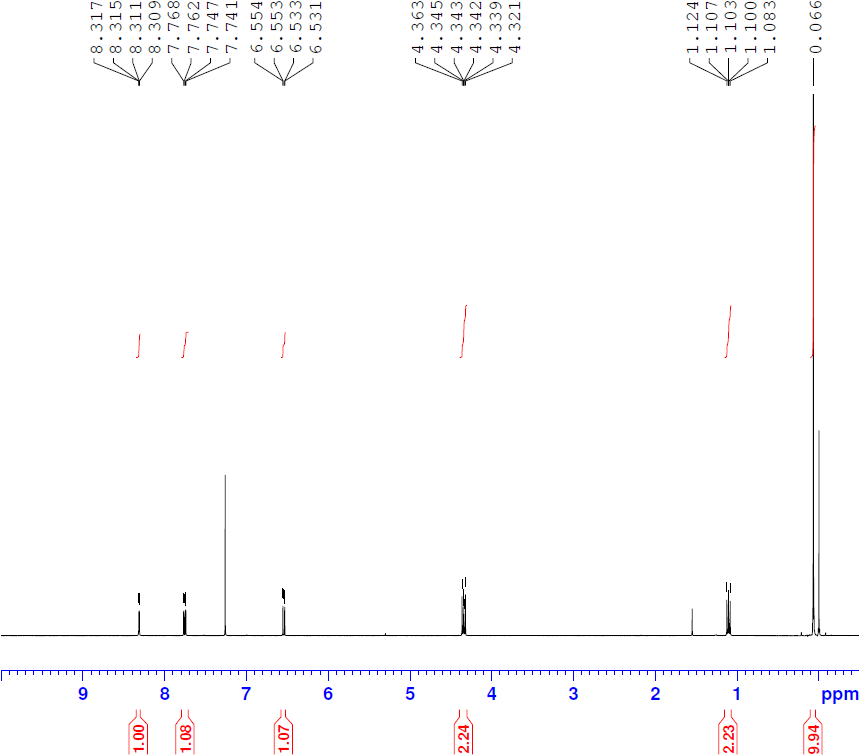


^13^C NMR (CDCl_3_, 151 MHz)

**Compound S5**

^1^H NMR (CDCl_3_, 400 MHz)

^13^C NMR (CDCl_3_, 151 MHz)

**Compound 2**

^1^H NMR (CDCl_3_, 400 MHz)

^13^C NMR (CDCl_3_, 151 MHz)

**Compound 3**

^1^H NMR (CDCl_3_, 400 MHz)

^13^C NMR (CDCl_3_, 151 MHz)

**Compound 4**

^31^P NMR (CDCl_3_, 162 MHz)

**Compound 5**

^1^H NMR (CDCl_3_, 400 MHz)

^13^C NMR (CDCl_3_, 151 MHz)

**Compound 6**

^1^H NMR (CDCl_3_, 400 MHz)

^13^C NMR (CDCl_3_, 151 MHz)

**Compound 7**

^1^H NMR (CDCl_3_, 400 MHz)

^13^C NMR (CDCl_3_, 151 MHz)

**Compound 8**

^31^P NMR (CDCl_3_, 162 MHz)

**Compound S6**

^1^H NMR (CDCl_3_, 400 MHz)

^13^C NMR (CDCl_3_, 151 MHz)

**Compound 9**

^1^H NMR (CDCl_3_, 400 MHz)

^13^C NMR (CDCl_3_, 151 MHz)

**Compound S7**

^1^H NMR (CDCl_3_, 400 MHz)

^13^C NMR (CDCl_3_, 151 MHz)

**Compound S8**

^1^H NMR (CDCl_3_, 400 MHz)

^13^C NMR (CDCl_3_, 151 MHz)

**Compound 10**

^1^H NMR (CDCl_3_, 400 MHz)

^13^C NMR (CDCl_3_, 151 MHz)

**Compound 11**

^1^H NMR (CD_3_OD, 400 MHz)

^13^C NMR (CD_3_OD , 151 MHz)

**Compound 12**

^1^H NMR (CDCl_3_, 400 MHz)

^13^C NMR (CDCl_3_, 151 MHz)

**Compound 13**

^1^H NMR (CDCl_3_, 400 MHz)

^13^C NMR (CDCl_3_, 151 MHz)

**Compound 14**

^1^H NMR (CDCl_3_, 400 MHz)

^13^C NMR (CDCl_3_, 151 MHz)

**Compound S9**

^1^H NMR (CDCl_3_, 400 MHz)

^13^C NMR (CDCl_3_, 151 MHz)

**Compound 15**

^1^H NMR (CDCl_3_, 400 MHz)

^13^C NMR (CDCl_3_, 151 MHz)

**Compound 16**

^1^H NMR (CDCl_3_, 400 MHz)

^13^C NMR (CDCl_3_, 151 MHz)

**Compound 17**

^31^P NMR (CDCl_3_, 162 MHz)

**Compound 19**

^1^H NMR (CDCl_3_, 400 MHz)

^13^C NMR (CDCl_3_, 151 MHz)

**Compound 20**

^1^H NMR (CDCl_3_, 400 MHz)

^13^C NMR (CDCl_3_, 151 MHz)

**Compound 21**

^31^P NMR (CDCl_3_, 162 MHz)

**Compound S10**

^1^H NMR (CD_3_OD, 400 MHz)

^13^C NMR (CDCl_3_, 151 MHz)

**Compound S11**

^1^H NMR (CD_3_OD, 400 MHz)

^13^C NMR (CD_3_OD, 151 MHz)

**Compound S12**

^1^H NMR (CD_3_OD, 400 MHz)

^13^C NMR (CD_3_OD, 151 MHz)

**Compound S13**

^1^H NMR (CDCl_3_, 400 MHz)

^13^C NMR (CDCl_3_, 151 MHz)

**Compound S14**

^1^H NMR (CD_3_OD, 400 MHz)

^13^C NMR (CD_3_OD, 151 MHz)

**Compound S15**

^1^H NMR (CDCl_3_, 400 MHz)

^13^C NMR (CD_3_OD, 151 MHz)

**Compound S16**

^1^H NMR (CD_3_OD, 400 MHz)

^13^C NMR (CD_3_OD, 151 MHz)

**16. References**

1. J. B. Foresman, and D. J. Fox, Gaussian, Inc., Wallingford CT, **2016**.
2. W. Shu and S. L. Buchwald, *Chem. Sci.*, **2011**, *2*, 2321-2325; T. Kinzel, Y. Zhang, S. L. Buchwald, *J. Am. Chem. Soc*., **2010**, *132*, 14073-14075.
3. S. R. Walker, M. L. Czyz, J. C. Morris, *Org. Lett.*, **2014**, *16*, 708-711.
4. X. J. Lu and W. K. Olson, *Nucleic Acids Res.*, **2003**, *31*, 5108-5121.
